# Targeted protein degradation reveals BET bromodomains as the cellular target of Hedgehog pathway inhibitor-1

**DOI:** 10.1038/s41467-023-39657-1

**Published:** 2023-07-01

**Authors:** Meropi Bagka, Hyeonyi Choi, Margaux Héritier, Hanna Schwaemmle, Quentin T. L. Pasquer, Simon M. G. Braun, Leonardo Scapozza, Yibo Wu, Sascha Hoogendoorn

**Affiliations:** 1 Department of Organic Chemistry, Faculty of Sciences, University of Geneva, Geneva, Switzerland; 2 School of Pharmaceutical Sciences, University of Geneva, Geneva, Switzerland; 3 Institute of Pharmaceutical Sciences of Western Switzerland, University of Geneva, Geneva, Switzerland; 4 Department of Genetic Medicine and Development, Faculty of Medicine, University of Geneva, Geneva, Switzerland; 5 Chemical Biology Mass Spectrometry Platform (CHEMBIOMS), Faculty of Sciences, University of Geneva, Geneva, Switzerland

**Keywords:** Target identification, Morphogen signalling, Proteolysis, Proteomics

## Abstract

Target deconvolution of small molecule hits from phenotypic screens presents a major challenge. Many screens have been conducted to find inhibitors for the Hedgehog signaling pathway – a developmental pathway with many implications in health and disease – yielding many hits but only few identified cellular targets. We here present a strategy for target identification based on Proteolysis-Targeting Chimeras (PROTACs), combined with label-free quantitative proteomics. We develop a PROTAC based on Hedgehog Pathway Inhibitor-1 (HPI-1), a phenotypic screen hit with unknown cellular target. Using this Hedgehog Pathway PROTAC (HPP) we identify and validate BET bromodomains as the cellular targets of HPI-1. Furthermore, we find that HPP-9 is a long-acting Hedgehog pathway inhibitor through prolonged BET bromodomain degradation. Collectively, we provide a powerful PROTAC-based approach for target deconvolution, that answers the longstanding question of the cellular target of HPI-1 and yields a PROTAC that acts on the Hedgehog pathway.

## Abstract (web summary)

Understanding the cellular target of hit compounds from phenotypic screens presents a major challenge yet is essential in the development of chemical probes. Here, the authors reveal the target of Hedgehog Pathway Inhibitor-1, by converting it to a bifunctional degrader, to be BET bromodomains.

## Introduction

The Hedgehog (Hh) pathway is a complex cellular signaling cascade that regulates embryonic developmental processes, such as patterning, as well as stem cell maintenance and tissue homeostasis^1^. Dysregulation of physiological levels of Hedgehog signal transduction results in developmental disorders and the onset and progression of various cancers, most notably basal cell carcinoma and medulloblastoma^2,3^. Pathway activation under normal conditions is initiated by the binding of one of the Hedgehog proteins (IHH, DHH, and SHH) to the receptor patched (PTCH1)^4,5^. The binding of HH to PTCH1 releases the latter’s inhibitory effect on smoothened (SMO)^6^. Further activation steps include the trafficking of GLI2/3 transcription factors bound to suppressor of fused (SUFU) through, and accumulation at the tip of, the primary cilium^7–9^. Processing of the GLI transcription factors into their transcriptionally active form then results in the transcription of Hedgehog target genes, amongst which the positive regulator *Gli1* and, in a negative feedback loop, *Ptch1*^10,11^. At present, the only clinically approved drugs to combat Hh pathway-driven cancers are those directed at SMO (vismodegib, sonidegib). Cancers driven by downstream pathway activation are inherently insensitive to those drugs and acquired resistance of initially responsive tumors is common^12–14^. Strategies to inhibit the Hedgehog pathway beyond smoothened are scarce, with only a handful of reported molecules with well-defined cellular targets and mechanism-of-action, including the ciliobrevins^15^, arsenic trioxide^16^, physalin H^17^, and Glabrescione B^18^. The majority of reported molecules have been identified through phenotypic screens for the Hedgehog pathway, and it has proven highly challenging to unravel the cellular target and molecular mechanisms of these hit compounds, severely limiting their use as chemical probes or therapeutic leads^19–25^.

Exemplary of this is Hedgehog Pathway Inhibitor (HPI-1), a dihydropyridine molecule discovered by Hyman et al. as a robust downstream Hh signaling inhibitor with anti-cancer properties^20,26,27^. Often referred to as a GLI inhibitor^28,29^, its cellular target has remained elusive for many years. Here, we present a target-identification methodology based on targeted protein degradation coupled with label-free quantitative proteomics. Using a Hedgehog Pathway Proteolysis-Targeting Chimera (PROTAC) (HPP), a bifunctional molecule consisting of HPI-1 coupled to a cereblon (CRBN) ligand, we elucidate BET bromodomains as the cellular targets of HPI-1. Moreover, we show that degradation of BET bromodomains through HPP-9 results in extended modulation of the Hedgehog pathway, suggesting alternative pharmacological strategies for this important developmental pathway.

## Results

### Design and synthesis of Hedgehog Pathway PROTACS

Proteolysis-targeting chimeras (PROTACs) are bifunctional molecules that induce proteasome-mediated degradation of a protein of interest (POI) through the formation of a ternary complex between the POI and an E3 ligase^30,31^. PROTACs have found widespread use as target validation tools, and as promising therapeutic leads because of their unique mechanism of action.

**Fig. 1:**
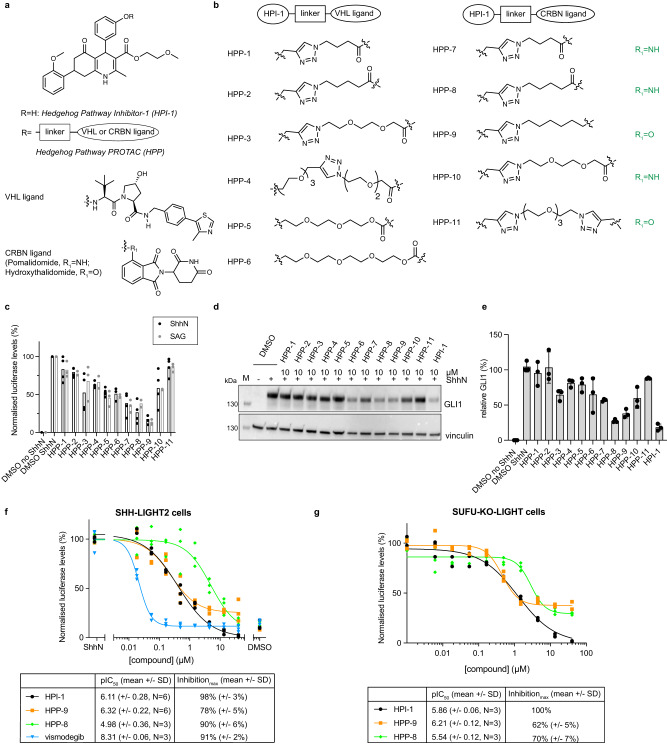
Design, synthesis, and biological evaluation of Hedgehog pathway PROTACs. **a** Structure of HPI-1, VHL ligand, and CRBN ligand and schematic design of the PROTACs. **b** HPP-1 to HPP-11 varied in the linker used to connect the E3 ligase ligand (VHL or CRBN ligand) to HPI-1. **c**–**e** The HPPs were evaluated in a luciferase assay in SHH-LIGHT2 cells stimulated with ShhN or SAG (**c**, mean  of *N* = 2 (+SAG for HPP-4, −6, −8), *N* = 3 (+SAG, +ShhN for HPP-4, −6), *N* = 4 (+ShhN) independent experiments) and by western blot for GLI1 levels for their inhibitory potential at 10 μM (**d**, **e**). Representative immunoblot (**d**) and quantification of band intensity (**e**, mean ± SD) of three independent experiments. **f**, **g** Full dose-response curves for most potent analogs HPP-8 and HPP-9 in SHH-LIGHT2 cells stimulated with ShhN (**f**) or constitutively active SUFU-KO-LIGHT cells (**g**). Representative curves (three technical replicates) of *N* = 3 or 6 independent experiments (as indicated in the table under the graph) are shown. Source data are provided as a Source Data file.

We hypothesized that we could extend the use of PROTACs as a target deconvolution method for hits from phenotypic screens through quantitative comparative proteomics. For this, we synthesized a small library of PROTACs, based on Hedgehog Pathway Inhibitor-1 (HPI-1, Fig. 1a, b). Building on and expanding existing structure-activity relationship (SAR) studies^32^, we found the phenolic hydroxyl to be the optimal position to introduce functionality, as its modification did not change the overall inhibitory potency of the molecule and it can be modified using late-stage functionalization (Supplementary Fig. 1). To convert HPI-1 into a bifunctional degrader molecule, we explored various linkers (polyethylene glycol of different length, simple aliphatic chains), attachment chemistries (triazole, ether, amide), and E3 ligase targeting ligands (VHL peptide ligand^33^, pomalidomide^34^, and hydroxythalidomide^35^) and assessed the inhibitory potencies of the resulting Hedgehog Pathway PROTACs (HPP-1 to HPP-11) in a variety of Hedgehog pathway activity assays (Fig. 1). SHH-LIGHT2 cells (NIH-3T3 cells stably expressing a GLI-driven luciferase reporter^36^) were stimulated with either Sonic Hedgehog-containing medium (ShhN) or the small molecule Smoothened agonist (SAG)^37^ in the presence of 10 μM of HPP (Fig. 1c). We found that HPPs incorporating a CRBN ligand were more potent than the VHL peptide analogs in inhibiting pathway activation. Furthermore, HPPs with short aliphatic linkers performed better than those with PEG linkers.

To exclude direct interference of the compounds with the luciferase enzymatic activity, the results were confirmed by western blot analysis for GLI1 protein expression (Fig. 1d, e). There was a good correlation between both readouts (Supplementary Fig. 2), and subsequently, full dose-response curves were generated for the HPPs that showed the lowest residual activity at 10 μM (HPP-8 and HPP-9). In contrast to the full Hh pathway inhibition that was obtained for the parent molecule HPI-1, inhibition by the PROTACs plateaued at 10–20% in SHH-LIGHT2 cells (Fig. 1f) and at 30–40% in constitutively activated SUFU-KO-LIGHT cells^28^ (Fig. 1g), which could point to a differential mode of action of the HPPs compared to the parent molecule. HPP-9 showed comparable potency to HPI-1 in both cell lines and was selected as the most suitable candidate for further studies.

### Methylation of thalidomide blocks HPP-9 activity

**Fig. 2:**
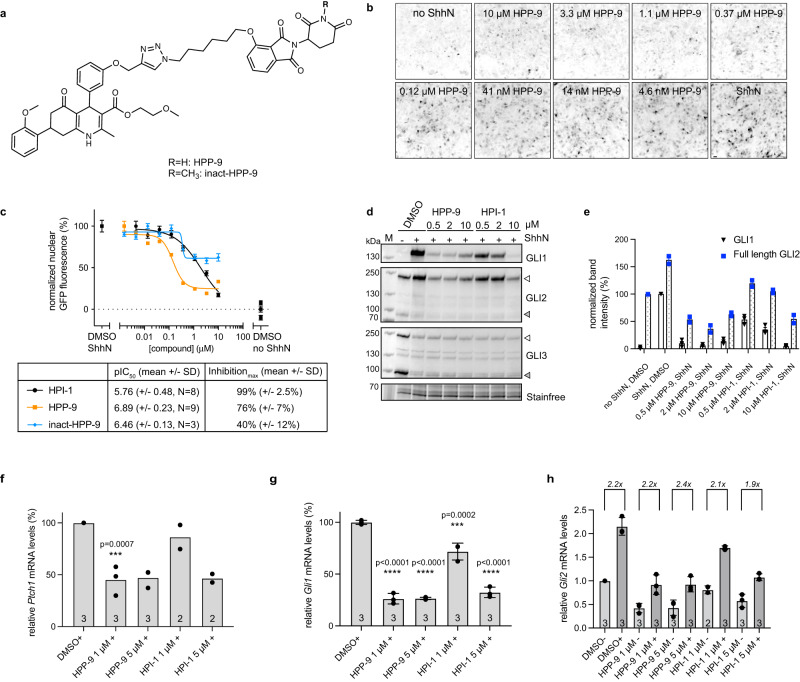
Biological evaluation of HPP-9 and its inactive analog. **a** Chemical structures of HPP-9 and the methylated analog inact-HPP-9. **b** Representative micrographs showing the dose-dependent inhibition of an Shh-driven GFP reporter by HPP-9, resulting in the dose-response curves shown in (**c**). Scalebar 30 μm. **b**, **c** Curves and images are representative of *N* independent experiments (as indicated in the table below the graph), with *n* = 9 or 18 images analyzed per experiment. **d**, **e** NIH-3T3 cells were incubated with increasing concentrations of HPP-9 or HPI-1 in the presence of ShhN and lysates were probed for GLI1, GLI2, and GLI3. **d** shows a representative immunoblot illustrating that both compounds inhibit GLI1 and GLI2 but have no effect on GLI3 processing. **e** Quantification (mean ± SEM) of GLI1 (*N* = 3) and GLI2 full-length levels (*N* = 2) of *N* independent experiments. **f**–**h** qPCR for *Ptch1* (***f***), *Gli1* (***g***), and *Gli2* (***h***) shows that HPP-9 reduces the expression of Hh pathway target genes, while also decreasing basal *Gli2* transcript levels without affecting the fold-induction upon pathway stimulation (−: no ShhN, +: with ShhN). Data shown is the mean (**f**) or mean ± SD (**g**, **h**) for *N* independent experiments as indicated in the bar. **f**, **g** One-way ANOVA with Dunnett’s test, *p* as indicated in the graph, compared to DMSO + ShhN. Source data are provided as a Source Data file.

As the target of HPI-1 is unknown, the mode of action of HPP-9 as a PROTAC could not be directly proven, and its inhibitory effect on the Hedgehog pathway could be the result of direct inhibition and/or degradation of the HPI-1 target. We sought to differentiate between these options by implementing a small structural change to the thalidomide core (methylation) such that the molecule could no longer bind to CRBN (Fig. 2a)^38^. We developed a high throughput microscopy assay using NIH-3T3 cells containing an Hh pathway-driven GFP reporter (SHH-GFP cells)^39^, to assess the inhibitory potency of this degradation-deficient HPP-9 analog (inact-HPP-9) (Fig. 2b, c). We found that inact-HPP-9 was indeed a very poor inhibitor compared to HPI-1 or HPP-9. Furthermore, in this assay, we observed a bell-shaped curve for HPP-9, which could be indicative of the presence of a Hook effect for this compound. When cells were incubated with various concentrations of HPP-9 or HPI-1 and analyzed by western blot for GLI protein expression or processing (Fig. 2d, e), we observed a similar trend. As previously reported for HPI-1^20^, no effect on GLI3 processing was found for either compound, indicating that these compounds act downstream of GLI3 activation at the level of the primary cilium. Whereas HPI-1 inhibited ShhN-induced GLI1 expression and reduced the GLI2 full-length/activator levels in a dose-dependent fashion (Fig. 2d, e), the effect of HPP-9 showed again an inverse correlation where 10 μM of the compound was less efficient than 0.5 or 2 μM. We then assessed the induction of Hh pathway target genes (*Gli1*, *Ptch1*) by qPCR (Fig. 2f, g), and in agreement with what was found on the protein level by western blot, we found a significant reduction in transcript levels^11^. As GLI2 protein levels were reduced below the basal level for HPP-9 and the highest concentration of HPI-1, we performed a qPCR for *Gli2* (Fig. 2h) and found levels to be much lower in both the minus and plus ShhN conditions^11^. Interestingly, the fold-induction upon the addition of ShhN remained constant at slightly over twofold under all conditions. Taken together, these results strongly suggested that HPP-9 acts as a PROTAC and is thus a promising lead for our proteomic target-identification studies.

### Proteomics reveals BET Bromodomains as putative HPP-9 targets

**Fig. 3:**
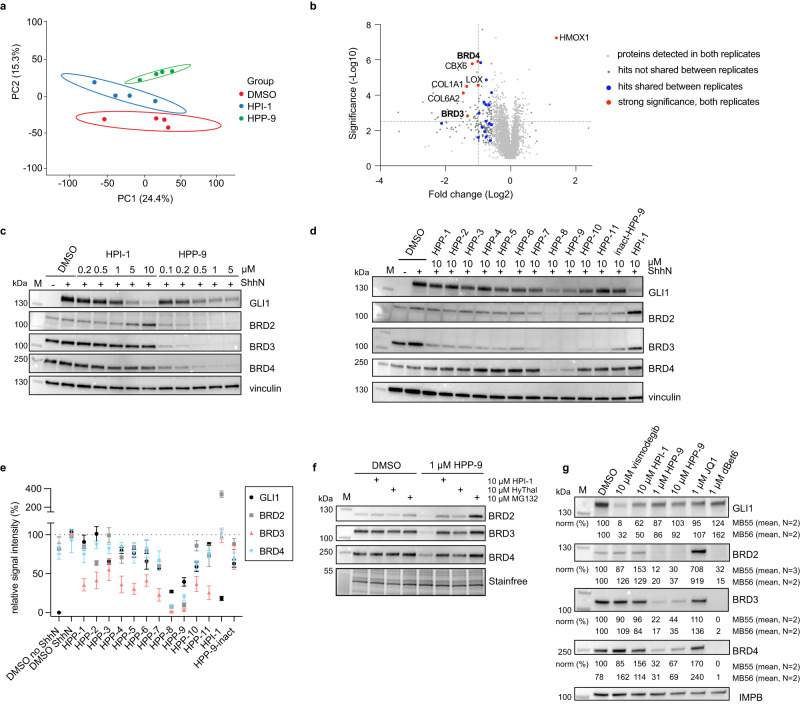
HPP-9 acts as a PROTAC for BET bromodomains. **a** Principal component analysis of 5343 quantified proteins clearly separates all sample groups. **b** Volcano plot of log2(fold change) to significance shows the potential direct targets of HPP-9. Blue circles represent all hits overlapping between replicates and red circles with text labels are the most significant hits. **c** HPP-9 dose-dependently degrades the BET bromodomain proteins, as shown by western blot analysis. Representative blot of three independent experiments. **d**, **e** All HPPs were profiled for their ability to degrade BRD2/3/4 by western blot, which revealed (**e**) a good correlation between Hedgehog pathway inhibition (Fig. 1e, replotted for clarity) and BET bromodomain degradation. Mean ± SEM of three independent experiments is plotted. **f** Representative immunoblot of a competition experiment between HPP-9 and HPI-1, hydroxythalidomide, and MG-132. Three independent experiments. **g** HPP-9 is able to degrade BET proteins in medulloblastoma cells MB55 and MB56. Representative immunoblot (MB56) of *N* = 2 independent experiments for each cell line. Normalized band signal intensities are shown underneath the blots. Source data are provided as a Source Data file.

We hypothesized that we could identify the target of HPI-1 through targeted protein degradation using HPP-9. For this, we applied label-free quantitative proteomics on cells treated with DMSO, 1 μM HPI-1, or 1 μM HPP-9 for 27 h. We applied data-independent acquisition mass spectrometry (DIA-MS) and quantified 5343 proteins across 12 samples. Expression of the 5343 proteins clearly separated the three sample groups under unsupervised clustering, with the HPI-1 group being intermediate between the DMSO and the HPP-9 group, suggesting distinguishable proteome changes in response to different treatments (Fig. 3a and Supplementary Data 1). In a second replicate of the experiment, we included 1 μM hydroxythalidomide as a control, to further refine our data. Between the two replicate experiments, we identified 3876 proteins that were detected in both datasets (Supplementary Data 1), and for our further analysis, we focused on those.

We analyzed our data for proteins that were significantly (fold change >1.5, *Q* value <0.05) changed in our HPP-9 samples, but not in any of the control samples (Fig. 3b and Supplementary Data 2). We expected that HPI-1 or hydroxythalidomide treatment results in changes in the proteome that would be mirrored by HPP-9, as they have shared biological activity. As HPP-9 is designed to act as a PROTAC, it should lead to the degradation of its protein target(s), whereas it is not expected that HPI-1 or hydroxythalidomide alone would do the same. We found 23 proteins to be shared hits between the two HPP-9 replicates, with only HMOX6 being upregulated (Fig. 3b, blue and red dots). Using more stringent significance criteria on the downregulated proteins (log2(fold change) <−1, −log 10 (*Q* value) >2.5), we arrived at six unique HPP-9 hits (Fig. 3b, red dots, labeled).

The significant depletion of two members of the bromodomain and extra-terminal domain (BET) protein family (BRD3 and BRD4) exclusively in the HPP-9 treated samples caught our attention. The role of the BET bromodomains (BRD2/3/4 and testis-specific BRDT) and well-known inhibitors thereof (JQ1^40^, iBET-151^41^) in epigenetic modulation of the Hh pathway is well-established. It has been shown that the genetic knockdown of BRD2/3/4 results in diminished pathway activation. Furthermore, BRD4 has been found to bind the *Gli1* and *Gli2* promotor regions and BET bromodomain inhibitors such as JQ1 have been shown to block BRD4 recruitment to these loci, thereby inhibiting Hh signaling^40,41^. All available data on HPI-1 as a downstream inhibitor point towards it regulating GLI transcriptional activity, making BET bromodomains likely relevant targets. A major advantage of our approach is that we could directly test this hypothesis by using our PROTAC HPP-9 and evaluating BRD protein degradation.

### HPP-9 is a potent BET bromodomain degrader

To assess the capability of HPP-9 to degrade BET bromodomains, NIH-3T3 cells were incubated with increasing concentrations of HPP-9 for 27 h in the presence of ShhN and probed for GLI1 (as a Hedgehog pathway readout), BRD2, BRD3, and BRD4. As shown in Fig. 3c, all three BRDs were almost completely degraded by our PROTAC at concentrations between 0.5 and 5 μM. In contrast, the probe was less effective at 10 μM, mirroring the Hook effect we observed before in our Hedgehog pathway assays (Fig. 2d and Supplementary Fig. 3). We then profiled our original library of HPPs for their ability to degrade BRD2/3/4 (Fig. 3d) and found a good correlation between the degradation efficiency and extend of Hedgehog pathway inhibition for all PROTACs (Figs. 1e, 3e). As expected, the inact-HPP-9 was completely incapable of degrading the BET bromodomains.

To prove that HPP-9 acts through a typical PROTAC mechanism, we performed competition assays with an excess of the proteasome inhibitor MG-132 (to prevent proteasomal degradation), the CRBN ligand hydroxythalidomide (to block all available CRBN E3 ligase), and the parent HPI-1 (to compete for binding of HPP-9 to BRDs) (Fig. 3f). In all cases, degradation was completely prevented, confirming that HPP-9 degrades BRDs through the formation of a ternary complex between BET bromodomains and CRBN, and subsequent proteasomal degradation.

We then assessed the specificity of HPP-9 and HPI-1 for Hedgehog pathway inhibition over a variety of other cellular signaling pathways. We included the smoothened inhibitor vismodegib as a control, as well as the BET inhibitor JQ1 and its PROTAC derivative dBet6^42^. None of these compounds inhibited platelet-derived growth factor-mediated phosphorylation of pERK1/2 or MAPK (Supplementary Fig. 4). No effect was found either when cells were stimulated with TNFα or Il1β to induce nuclear translocation of p65, as one of the upstream events in NF-κB signaling (Supplementary Fig. 5). We then turned to transcriptional luciferase reporter assays for Wnt signaling and TNFα-induced NF-κB signaling and found that, as reported before, HPI-1 does not inhibit Wnt signaling^20^. HPP-9 was found to have some inhibitory effect on Wnt signaling, but was an order of magnitude less potent than in Hedgehog signaling assays (Supplementary Fig. 6). Neither compound inhibited TNFα-induced NF-κB signaling (Supplementary Fig. 7).

Next, we investigated the ability of HPP-9 to degrade BET bromodomains in cancer cell lines, and its effect on GLI1 as a readout of Hedgehog pathway state. In line with what was found in fibroblast cells, HPP-9 only partially reduced GLI1 levels in A549 human lung adenocarcinoma cells, whereas HPI-1 and JQ1 were more effective^43^. A similar hook effect for HPP-9, as observed earlier (Supplementary Fig. 3), was detected for BET bromodomain degradation in these cells (Supplementary Fig. 8). Of note, treatment with 1 μM dBet6 caused significant cell death, and so this condition had to be excluded from the experiment. In mouse medulloblastoma spheroids (MB55 and MB56^44^), only vismodegib was found to significantly reduce GLI1 levels (Fig. 3g). Surprisingly, strong bromodomain degradation by dBet6 or HPP-9 was found to be ineffective in reducing GLI1 levels, and in contrast to findings in A549 or NIH-3T3 cells, BRD2 levels were increased by JQ1 and not HPI-1.

### HPI-1 is a high-affinity BET bromodomain binder

**Fig. 4:**
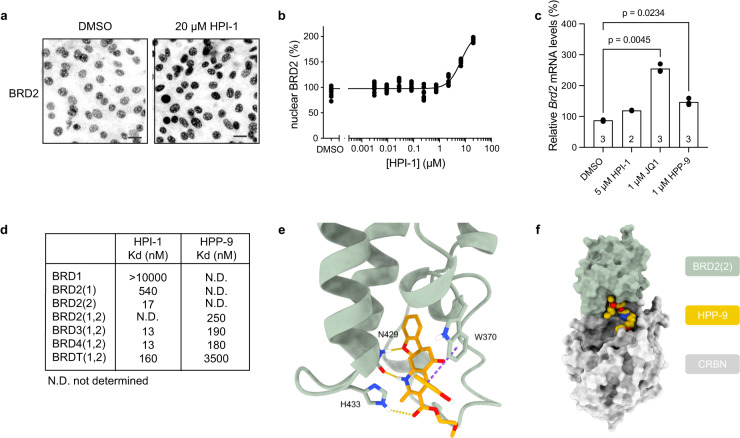
HPI-1 is a high-affinity BET bromodomain binder that increases cellular BRD2 levels. **a** Representative micrographs of SHH-GFP cells treated with DMSO or 20 μM of HPI-1 for 27 h. Scalebar 25 μm. **b** High-content microscopy dose-response curve for cells treated with increasing concentrations of HPI-1 and probed for nuclear BRD2. Representative curve from *N* = 3 independent experiments, with *n* = 9 images analyzed per condition. **c** qPCR analysis of *Brd2* mRNA levels from cells treated with 5 μM HPI-1, 1 μM HPP-9 or JQ1 for 27 h in the presence of ShhN. Data from *N* independent experiments, as indicated in the bars, performed in duplicate. One-way Brown–Forsythe and Welch ANOVA, Dunnett T3 test. **d** The affinity of HPI-1 and HPP-9 to the indicated bromodomains (BromoKd, Eurofins discovery). (1,2) indicates which of the two bromodomain subunits were included. **e** Proposed binding mode for HPI-1. The interacting residues and HPI-1 are modeled in stick, while BRD2(2) is shown in cartoon representation. The dashed yellow lines represent H-bonds while the π-π stacking is shown as a purple dashed line. **f** Possible physical model of the ternary complex. BRD2(2) (green) and CRBN (gray) are shown in a surface representation, while HPP-9 (orange) is shown as spheres. Source data are provided as a Source Data file.

Through our competition assays (Fig. 3f), we found that HPI-1 can prevent HPP-9-induced BRD2/3/4 degradation, which strongly indicates that they are acting on the same targets. As shown in Fig. 3d–f, we observed a strong increase in BRD2 protein levels by western blot when we incubated NIH-3T3 cells with increasing concentrations of HPI-1 for 27 h. This was validated by dose-response fluorescence microscopy to be around twofold more nuclear BRD2 at 20 μM of HPI-1 (Fig. 4a, b). We then wanted to confirm if this increase in protein levels is a result of enhanced *Brd2* transcription as reported for JQ1^45^. Indeed, by qPCR, we found *Brd2* to be highly expressed when treating the cells with 1 μM JQ1, and a much smaller increase was found with 5 μM of HPI-1 (Fig. 4c). However, no change in BRD2 protein could be detected when cells were treated with 1 μM JQ1 (Supplementary Fig. 9). Of note, BRD2 levels were increased in medulloblastoma cell lines when treated with JQ1 (Fig. 3g).

To further prove the direct interaction between HPI-1/HPP-9 and BET bromodomain proteins, the compounds were sent for affinity measurements (BromoKd assay, Eurofins Discovery) against BRD2/3/4 and BRDT. For HPI-1, we included the non-BET bromodomain BRD1 as this protein was also found to be downregulated by HPI-1 in one of our proteomics datasets. Since the effect on BRD2 protein levels when treating NIH-3T3 cells with HPI-1 was so large, we determined the affinity for the two different bromodomains of BRD2, BD1, and BD2, separately. As shown in Fig. 4d, HPI-1 is a high-affinity bromodomain binder with low nM K_d_ for BRD2/3/4 and about tenfold lower affinity for BRDT. HPI-1 showed a much higher affinity to BD2 of BRD2 (17 nM), than to BD1 (540 nM), but otherwise, no apparent selectivity between BRD2/3/4. No binding for BRD1 could be detected, indicating that the reduction in protein levels found by proteomics likely arose from an indirect effect. The affinity of HPP-9 was an order of magnitude lower than that of HPI-1 (~200 nM versus ~15 nM), which agrees with the low inhibitory potency of inact-HPP-9 and confirms that HPP-9 inhibits mostly through degradation and not direct inhibition.

To get insights into a potential binding mode of HPI-1, we docked the compound in the bromodomain for which high affinity has been measured unequivocally, namely the second bromodomain of BRD2 (BRD2(2)) (Fig. 4d). We used different crystal structures in both apo and holo form to explore the variety of conformations of the binding site. All binding poses with a Glide gscore better than −6 kcal/mol have been selected and clustered. This first analysis reveals three plausible binding modes. To validate the models, we used the information from the ligand-based SAR of the HPI-1 derivatives of this paper (Supplementary Fig. 1) and the analogs synthesized in the context of the HPI-1 patent^32^. The result of this validation suggests that the model where HPI-1 forms strong interactions with the amino acids forming the binding site is the one providing a three-dimensional explanation of the ligand SAR (Fig. 4e). In particular, the conserved residue N429 forms the same network of H-bonds with HPI-1 as other inhibitors^46–48^. HPI-1 interacts via a T-shaped π-π stacking with tryptophan 370 (W370). The carbonyl of the ester group binds to histidine 433 (H433) being the H-bond donor. This last interaction might explain the difference in affinity between the second and the first bromodomain of BRD2, where histidine is replaced by an aspartate, abrogating this predicted H-bond.

The SAR study performed for the PROTAC design (Supplementary Fig. 1) concluded that the substitution of an alkyl to a propynyl group in position R1 and R2 is not tolerated. The R1 group corresponds to the methoxy group. In the docking pose, the methyl from the methoxy group of R1 interacts with hydrophobic residues forming a defined subpocket (Y428, L383, V376, and Y386) (Supplementary Fig. 10). The substitution of the methyl for the bulkier propynyl group abrogates activity because of the lack of space to accommodate it. The functionalization of the nitrogen atom of R2 also leads to a loss of activity, which is explained by the loss of an H-bond and the generation of a steric hindrance because of the reduced space in this part of the binding site cavity. The remaining positions, R3, R4, and R5 are all solvent-exposed hence their substitution does not hamper binding into the binding pocket, as reflected by the relatively unchanged pIC_50_.

The SAR resulting from the data of the HPI-1 patent provided additional material for the model validation and highlighted the most important interactions (Supplementary Fig. 11)^32^. The phenyl group carrying the methoxy group (R1) appears to anchor the ligand at the bottom of the pocket thanks to hydrophobic interactions and a potential π-π stacking with Y386 for the phenyl group, and an H-bond with N429 for the methoxy group. Both features lead to high activity, while their absence decreases dramatically the activity as seen in compounds **22,** **23,** **24**, and **25**. It is worth to note that the presence of the aromatic ring allows for sparing some activity for compound **21** that lacks only the methoxy group.

Our 3D model suggests that the hydroxyl group on the phenyl ring of position R5 is not forming strong interactions. Indeed, compound **29**, lacking this group, retains the same activity. The OH-group is well tolerated both in position meta and para, as seen by compound **31**. The increased activity when the hydroxyl is placed in ortho (compound **35**) could be due to intramolecular H-bond formation that locks HPI-1 into a favorable conformation reinforcing the T-shaped π-π stacking of the phenyl moiety with W370. The replacement of the hydroxyl with a pure hydrogen bonding acceptor group like a methoxy did not significantly affect the activity (compounds **32** and **34**) with the exception of compound **33**, that has a better activity that might come from the peculiarity of methoxy groups in meta-substitution that influence the π-cloud of the phenyl moiety favoring the π-π stacking with W370. The absence of this last interaction leads to a 1 log-fold decrease in activity (compound **30**). A non-aromatic ring also decreases the activity, possibly through the loss of the possibility of forming π-π stacking (compounds **36**, **42**, and **43**). We hypothesize that the loss of binding affinity in the case of HPP-9 compared to HPI-1 is not due to the loss of a strong interaction between the hydroxyl group and the protein, but rather to subtle rearrangements of the ligand inside the pocket due to the high dynamic nature of the linker, that could destabilize the interactions.

To conclude, the number and type of the interactions found within the obtained model (Fig. 4e and Supplementary Fig. 10) are consistent with the high affinity found experimentally and recapitulate the ligand-based SAR extending it to 3D.

We then ran three independent unbiased molecular dynamic simulations of HPI-1 in complex with BRD2(2) (Supplementary Fig. 12) to assess both ligand stability along a 1 µs trajectory and the described SAR (accumulated time of 3 µs). The interactions predicted by the docking pose are recapitulated and most of them are stable along the course of the simulation (mean RMSD of 1 Å for the ligand in all replicas, Supplementary Fig. 12), a behavior compatible with a high-affinity ligand as shown with the binding experiment (Fig. 4d). The π-π stacking with W370 is intermittent: W370 switches its position back and forth between the rotamer forming the π-π stacking and another dominant rotamer that does not allow this interaction. The two H-bonds with N429 are present along the course of the simulation. The one involving the amide nitrogen occurs more frequently than the one involving the carbonyl oxygen. The methoxy group tends to rotate in order to maximize hydrophobic interactions of the methyl at the bottom of the pocket formed by V376, L383, Y386, and Y428.

In addition to the interactions found by docking, the simulation allowed us to uncover further interactions with other residues lining up the pocket. These additional interactions complement the information delivered by the docking model allowing a better understanding of the SAR. Summarizing, the binding of HPI-1 and BRD2(2) is dominated by hydrophobic interactions, and the highly aromatic environment formed by W370, F372, Y386, Y428, and H433 allows consistent π-π stacking along the trajectory. In particular, H433 interacts in the docking model via an H-bond with the carbonyl from the ester, but the simulation shows a possibility of π-π stacking with the phenyl group R5.

Finally, we sought to assess in silico the possibility of forming a physical ternary complex between BRD2(2), HPP-9, and CRBN, starting with the proposed binding mode of HPI-1. We repurposed an existing protocol developed for evaluating different linkers in PROTAC design^49^ to generate physical models. The protocol consists of three steps: the generation of BRD2(2)-CRBN complexes by protein–protein docking, the overlap of HPP-9-linker conformers on the resulting complexes, and finally, a minimization of the ternary complex. The complex that displays the best interaction score (Fig. 4f) shows that the linker of HPP-9 is long enough to allow both moieties to bind their respective protein and form a low-energy structure.

Finally, HPI-1 has been deposited in the public repository of the NCI-60 as a putative SMO inhibitor. We employed the NIH COMPARE algorithm (https://nci60.cancer.gov/publiccompare/), to find compounds that share the highest overlap in cellular activity profiles. We found that HPI-1 overlapped most significantly (Pearson’s *t*-test) with other annotated BET bromodomain inhibitors, including INCB-057643 (*R*^2^ > 0.67), JQ1 (*R*^2^ > 0.64), and I-BET151 (*R*^2^ > 0.64), providing further evidence of a shared cellular target.

### HPP-9 and dBet6 have a differential mechanism of action

**Fig. 5:**
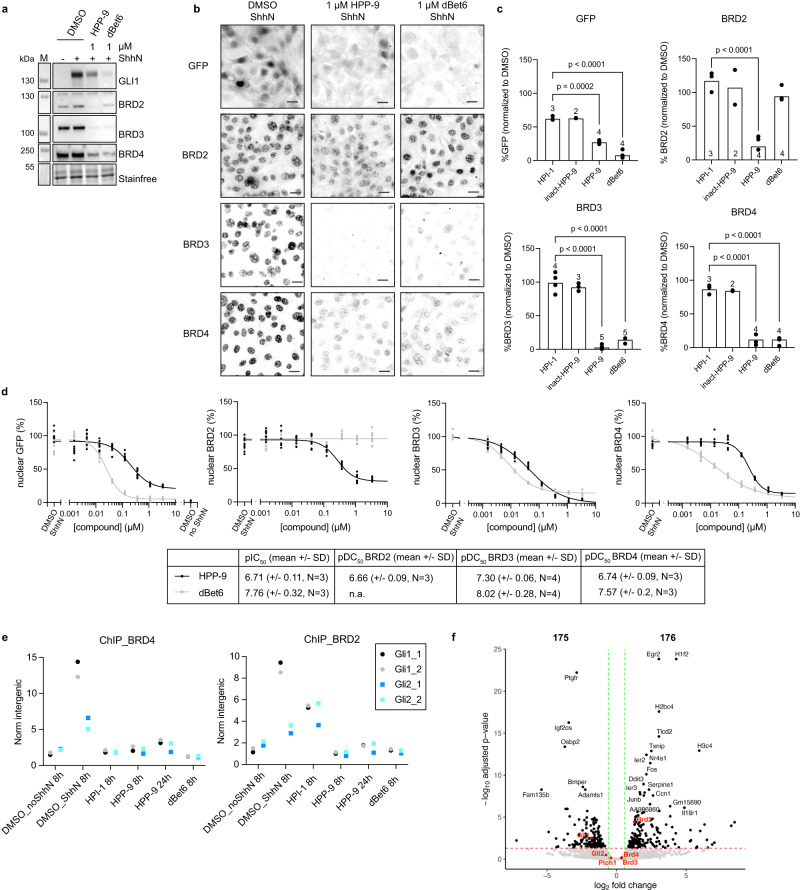
HPP-9 and dBet6 have a different mode of action. SHH-GFP cells were treated with 1 μM of HPP-9 or dBet6 for 27 h and probed for GLI1, BRD2, BRD3, and BRD4 by western blot (**a**) and analyzed by fluorescence microscopy (**b**, **c**). Representative micrographs showing the nuclear localization of GFP, BRD2, BRD3, and BRD4 are shown in (**b**), and the signal is quantified in (**c**). Scalebar 25 μm. Data shown is the mean from *N* independent experiments as indicated in or above the bars. At least seven images were analyzed per condition in each experiment. All compounds were used at 1 μM. Two-way ANOVA. **d** Full dose-response curves were measured by high-content microscopy to determine the pDC_50_ values for HPP-9 and dBet6. Each dot is an individual image (*n* is at least 7 images/experiment, *N* = 3, 4 independent experiments as indicated in the table under the graphs, n.a. not active). **e** BRD2 and BRD4 binding to *Gli1* and *Gli2* loci was measured by ChIP-qPCR using two primer pairs per locus. Representative data of two independent experiments. **f** Volcano plot showing differentially expressed genes in SHH-GFP cells upon 8 h of HPP-9 treatment (+ShhN) compared to DMSO + ShhN. Two independent experiments, *p*_adj_ < 0.05 and 1.5x fold change. Exact *p* values can be found in Supplementary Data 3 and were determined using the Wald test. The adjusted *p* values were corrected for multiple testing, using the Benjamin–Hochberg correction. Source data are provided as a Source Data file.

Various PROTACs targeting the BET bromodomains have been reported, including dBet6^42^ and MZ1^50^. Both dBet6 and MZ1 are JQ1-based PROTACs, but with different reported selectivity profiles. In Shh-LIGHT2 cells, we found MZ1 to be a Hh pathway inhibitor with a pIC_50_ of 6.8, but not a selective BRD4 degrader at relevant concentrations for inhibition (Supplementary Fig. 13). We profiled our HPP-9 against the pan-BET bromodomain degrader dBet6 and surprisingly, we found that these compounds behaved differently in both their ability to suppress Hh pathway activation and their degradation profiles (Fig. 5a–d). First, the partial inhibition of Hh signaling by HPP-9 was not found for dBet6, as it inhibited the Hh pathway slightly more potently (pIC_50_ 7.76 for dBet6 vs 6.71 for HPP-9) and fully. However, in contrast to previous reports^42,51^, no significant degradation of BRD2 was observed (Fig. 5b–d). Of note, these experiments were done under Hedgehog signaling conditions (1 μM of the compound in the presence of ShhN for 27 h), to simultaneously assess the ability of the compounds to suppress the Hh pathway-dependent GFP reporter (Fig. 5b–d) or endogenous GLI1 expression (Fig. 5a) as well as bromodomain degradation (Fig. 5a–d). Indeed, when we performed a time-course assay to assess how fast HPP-9 degrades the BRDs (Supplementary Fig. 14), we included dBet6 as a control and found that dBet6 is an effective pan-bromodomain degrader at shorter timepoints, with complete degradation as soon as 2.5 h after addition to the cells. HPP-9-mediated degradation of BRD2/3/4 reaches a plateau around 7 h, and in contrast to dBet6, remains so at longer timepoints. As JQ1-based BET bromodomain inhibitors typically reduce cell viability in a dose-dependent fashion, we treated a variety of mouse (NIH-3T3 and IMCD3) and human (HEK293 and HeLa) cell lines with increasing concentrations of HPP-9 or dBet6 for 48 h and we found HPP-9 to be much less toxic than dBet6 (Supplementary Fig. 15a, b). Similarly, we found medulloblastoma spheroid cell lines (MB55 and MB56^44^) to be highly sensitive to JQ1, dBet6, and vismodegib. HPI-1 also killed the cells, albeit with lower potency (Supplementary Fig. 15c). HPP-9 was ineffective, but was found to precipitate from the culture medium, explaining the lack of effect in this assay (Supplementary Fig. 15). In combination with the western blot results (Fig. 3g), it appears that the cellular toxicity of JQ1 and dBet6 is not correlated to the SHH-dependency of these cells, but rather a result of modulation of other BET-driven transcriptional events.

To gain more mechanistic insights into the observed differences between compounds, we performed ChIP-qPCR experiments in NIH-3T3 cells to assess BRD2/4 engagement on the *Gli1* and *Gli2* loci (Fig. 5e). As expected, we found that both HPP-9 and dBet6 strongly reduced the ShhN-induced recruitment of these proteins. HPI-1 was able to completely block BRD4 from both *Gli1* and *Gli2* loci, similar to what has been reported for JQ1^40^. However, it only partially reduced BRD2 on the *Gli1* locus, and did not change BRD2 occupancy on the *Gli2* locus. This can possibly be explained by the induced *Brd2* mRNA/BRD2 protein levels upon HPI-1 treatment (Fig. 4b, c). Next, we assessed global transcriptional changes when treating cells with DMSO, HPP-9, HPI-1, or dBet6, in the presence of ShhN (Supplementary Data 3). Here, we found striking differences between compounds (Supplementary Fig. 16), where dBet6 treatment led to massive down- or upregulation of 5482 genes^52^, including downregulation of *Gli1*, *Brd3*, and an increase of *Brd2* (Supplementary Fig. 17*)*. Whereas *Brd4* was not significantly altered based on two replicate experiments, it also showed a downward trend (Supplementary Fig. 18). HPI-1 and HPP-9 showed much more subtle changes, with 99 and 351 significantly changed genes after 8 h of treatment, and 554 after 24 h of HPP-9 treatment (Supplementary Data 3). For both HPI-1 and HPP-9, we find *Brd2* to be upregulated, but no significant changes in *Brd3/Brd4* (Fig. 5f and Supplementary Figs. 17, 18). This further strengthens previous results showing that BET bromodomains are degraded by HPP-9, and that the decrease found by proteomics is not due to cellular adaptation. Of note, the other hits found in the proteomics experiment (Fig.3b, red dots), all trend in the same direction on the gene expression level, and, therefore, likely indicate indirect effects (Supplementary Fig. 19).

### HPP-9 is a long-acting Hedgehog pathway inhibitor

**Fig. 6:**
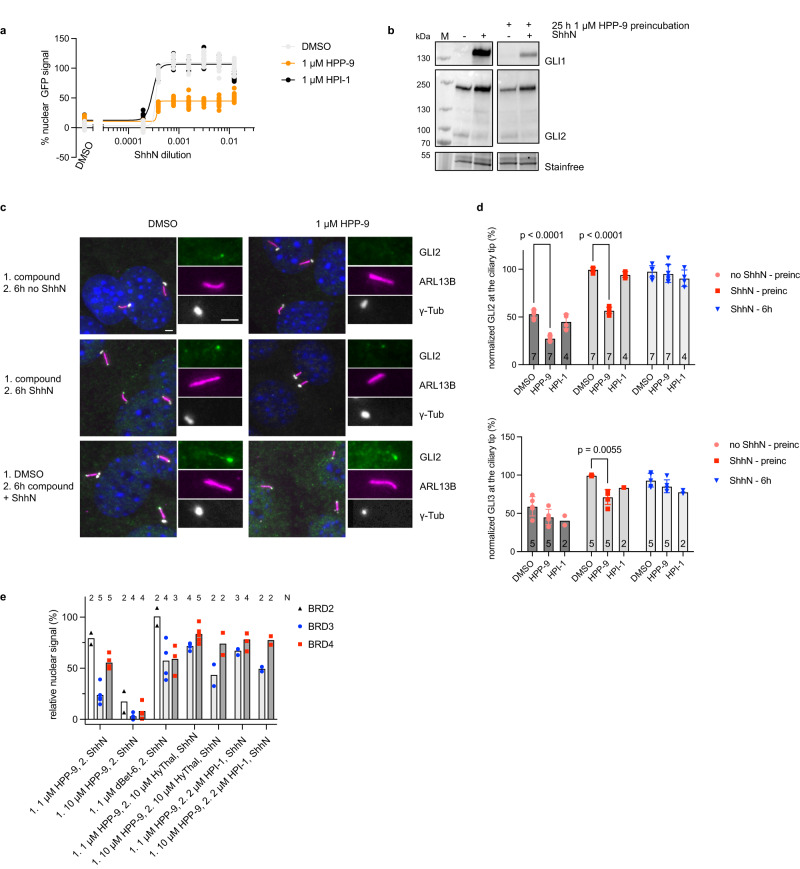
HPP-9 is a long-acting Hedgehog pathway inhibitor. **a** SHH-GFP cells were treated for 25 h with DMSO, 1 μM HPI-1, or 1 μM HPP-9, before the medium was changed to various dilutions of ShhN-conditioned medium for 27 h. Nuclear GFP levels were quantified using fluorescence microscopy. Representative curves of three independent experiments are shown, with 9–16 images analyzed per condition. **b** GLI1 and GLI2 levels of cells pre-incubated with DMSO or 1 μM HPP-9 were determined. Representative blots of three independent experiments are shown. **c**, **d** The effect of 1 μM HPP-9 or HPI-1 (pre-)incubation on GLI2 ciliary trafficking was assessed through fluorescence microscopy. Representative images for GLI2 trafficking are shown in (**c**), and all data is quantified in (**d**). *N* independent experiments as indicated in the bars, *n* = 300–500 cilia analyzed per condition. Mean ± SD is plotted, two-way ANOVA, *p* as indicated. Scalebar 2 μm. **e** SHH-GFP cells were incubated with the indicated compounds for 25 h, before the medium was changed to ShhN-conditioned medium with or without competitors for 27 h. Nuclear BRD protein levels were determined using high-content fluorescence microscopy. Data shown is from *N* independent experiments as indicated, with *n* = 9–16 images analyzed per condition in each experiment. Source data are provided as a Source Data file.

Intrigued by the differential degradation efficiency found between dBet6 and HPP-9 at later timepoints, we wondered if, rather than just a target-identification tool, HPP-9 could be an interesting Hedgehog pathway inhibitor in its own right. Clearly, HPP-9 is a partial inhibitor with suboptimal physicochemical properties for therapeutic use, but since its action is through degradation rather than inhibition, we hypothesized that it could act longer than the parent compound HPI-1. To test this hypothesis, we incubated SHH-GFP cells for 25 h with 1 μM of HPP-9 or HPI-1 and subsequently removed the compounds and induced the Hedgehog signaling pathway for 27 h (Fig. 6a, b). Strikingly, we found that the cells that were treated with HPP-9 had strongly reduced signaling output, both at the level of a GFP reporter (Fig. 6a), as well as endogenous GLI1/GLI2 levels (Fig. 6b), whereas cells pretreated with HPI-1 responded normally. One of the upstream activation events of the Hh pathway is the accumulation of GLI2 and GLI3 at the tip of the primary cilium, which is necessary for their subsequent activation into transcriptional activators^7^. As HPP-9-treated cells showed strongly reduced *Gli2* transcript levels in the absence of ShhN, as well as GLI2 full-length levels below untreated cells (Fig. 2e, h), we assessed the ability of cells to accumulate GLI proteins at the ciliary tip when (pretreated) with HPP-9 (Fig. 6c, d). For this, NIH-3T3 cells were pre-incubated for 24 h with 1 μM of HPP-9, HPI-1, or DMSO, and subsequently treated with ShhN for 6 h in the presence or absence of 1 μM HPP-9 or HPI-1. As shown in Fig. 6c, d, we found that the globally reduced GLI2 levels translate to those found at the ciliary tip when pre-incubating cells with 1 μM HPP-9. After pretreatment, cells could still accumulate GLI2/3 at the tip (1.5–2-fold), but overall levels were reduced by a factor 2 (GLI2) or 1.3 (GLI3). No differences were found at the 6 h timepoint, indicating that this is not an acute effect on ciliary trafficking but rather a consequence of transcriptional regulation of global GLI protein levels. (Pre)-incubation with 1 μM of HPI-1 did not show this effect (Fig. 6d), in line with the lack of change in *Gli2* transcript and GLI2 protein levels observed at this concentration (Fig. 2e, h).

We then determined levels of BET bromodomain proteins 27 h after the removal of HPP-9 (1 or 10 μM) or dBet6 (1 μM). As shown in Fig. 6e, pre-incubation with 10 μM HPP-9 resulted in almost complete and a much higher level of degradation compared to 1 μM HPP-9, whereas HPP-9 globally showed more degradation at this late timepoint than dBet6. As we observed such a strong Hook effect for HPP-9 compared to dBet6, we wondered if this compound could potentially be very difficult to wash out. In such a scenario, removal of the compound from the cell culture medium reduces the effective concentration to where the Hook effect is no longer in effect, rather than removing the compound altogether. We tested this by addition of either hydroxythalidomide (10 μM) or HPI-1 (2 μM) to the ShhN-containing medium after the pre-incubation with HPP-9 to compete with the formation of a ternary complex by HPP-9. Indeed, both compounds were able to partially restore the BET bromodomains suggesting that HPP-9 remains physically present in the cells after changing the medium, which results in its prolonged action.

## Discussion

Target deconvolution of small molecule hits from phenotypic screening campaigns remains a major challenge, and there is, unfortunately, no one-size fits all approach^19^. Here, we report a PROTAC-based strategy for target identification which we applied to discover the cellular targets of Hedgehog pathway inhibitor HPI-1. Since their discovery, PROTACs have found widespread use due to their unique mechanism of action, as probes for target validation and off-target discovery, and as drugs^30,31^. Recent studies furthermore describe the use of a PROTAC to discover alternative targets of a small molecule or to identify the target of a natural product^53,54^. To convert a hit into a PROTAC, it is necessary to first determine the structure-activity relationship for the parent molecule, which is often in itself part of a medicinal chemistry campaign and also a required step in the development of, for example, photo-affinity probes. Through our SAR analysis, we arrived at HPP-9, a PROTAC version of HPI-1 with similar Hedgehog pathway inhibitory activity, yet a partial rather than a full inhibitor. Using a quantitative label-free proteomics strategy, we were able to identify the BET bromodomains as potential target candidates. A major advantage of our approach is that we could directly verify the cellular target engagement using HPP-9. Indeed, HPP-9 is a potent BRD2/3/4 degrader, whose action can be competed with HPI-1, indicating that it robustly reports on the target of the parent molecule.

BET bromodomains are epigenetic modulators involved in a plethora of cellular processes^55,56^. Their involvement in the Hedgehog pathway has been partially deciphered through the use of the small molecule pan-BET bromodomain inhibitor JQ1, which decreases the binding of BRD4 to the *Gli1* and *Gli2* promoters, thereby regulating GLI activity^40^. In a genome-wide CRISPR knockout screen for modulators of ciliary Hedgehog signaling *Brd2* was found as a strong hit, whereas knockout of *Brd4* resulted in a growth phenotype^57^. Our attempts at generating individual *Brd2* and *Brd4* knockout NIH-3T3 cells were unsuccessful, but we could confirm the SHH-induced binding of BRD4 and BRD2 to the *Gli1* and *Gli2* loci by ChIP-qPCR, further validating their role in regulating GLI signal transduction.

When comparing HPP-9 to the widely used JQ1-based PROTAC dBet6, we observed many differences, even though both compounds degrade BET bromodomains. First, HPP-9 is much less cytotoxic, second, Hh pathway inhibition is partial while full for dBet6, and third, the timing and duration of degradation is very different. This raises the question if there is a specific timepoint after pathway induction where the BET bromodomains are most critical and that HPP-9 action is simply too late to effect full inhibition of Hedgehog signaling. Our RNA-seq data suggests that bromodomain degradation results in much more pronounced gene expression changes than only inhibition, as shown in the comparison between HPP-9 and HPI-1, in agreement with previous reports for JQ1 versus dBet6^42^. dBet6 treatment, however, results in the most dramatic changes, amongst which the induction of apoptotic pathway genes. It is, therefore, likely that this is the direct underlying cause of the observed cytotoxicity of dBet6. Indeed, it has been shown that BET degradation through dBet6 results in the global collapse of transcription elongation^42^. We find that dBet6 also downregulates *Brd3* and *Brd4* transcript levels, while increasing *Brd2*. HPP-9 on the other hand, exclusively decreases BRD2/3/4 at the protein level.

While the in vitro binding affinity of HPP-9 and HPI-1 for BRD2, 3, and 4 is similar, it does appear that HPP-9 (and HPI-1) have a particular effect on cellular BRD2, which is cell line dependent and differs from JQ1-based probes. Notably, HPP-9 differs completely from dBet6 in its degradation capacity at longer timepoints, illustrating how molecules that act on the same target can still have a different mechanism of action. We have no definitive proof at this point, but the most likely explanation for this is that HPP-9 remains stably inside the cells and is inefficiently washed out. We speculate that dBet6 is much less stable, leading to the reappearance of BRD2, while the decrease in mRNA for *Brd3* and *Brd4* may lead to a slower return of BRD3/4. While we found that in NIH-3T3 cells JQ1 increased *Brd2* transcript levels much more than HPI-1, the latter did increase BRD2 protein, whereas JQ1 did not. In medulloblastoma spheroid cells, however, we did find a profound JQ1-induced increase of BRD2 protein. The mechanism and the functional consequences of this are currently unknown. The data provided by our study clearly point towards mechanistic differences between HPP-9/HPI-1 and dBet6/JQ1, but the underlying reasons for the observed differences remain elusive. Possibly, differences in potency, BET bromodomain selectivity, compound stability in the medium and inside the cell, as well as the onset of inhibition/degradation, contribute to the observed differences. Alternative targets or off-targets for these compounds cannot be fully excluded at this point, especially in different cell types or cellular contexts. Further research efforts will be necessary to decipher the mechanism of action in more detail and to fully understand the contributions of the various BET bromodomains on Hh signal transduction, cell viability, and the effect of compounds on these.

No PROTACs have been reported to date that act on the Hh pathway, most likely because most molecules target the transmembrane protein smoothened. We demonstrated that BET bromodomain degradation through HPP-9 results in prolonged Hh pathway inhibition, in line with what has been shown for some other PROTACs^58^, but with less cytotoxicity and Hh pathway-independent off-targets as dBet6. This suggests that HPPs could form the basis for alternative therapeutic modalities targeting Hedgehog pathway-driven cancers. This would, however, require significant optimization of the physicochemical properties of the here reported molecules, as we found HPP-9 to be precipitating from spheroid medium and sensitive to serum concentration. Therefore, HPP-9 in its current form is limited in its use as a research tool for in-cell experiments, which prevented us from assessing if this therapeutic potential would hold true in an in vivo setting. Since the targets of HPI-1 and HPP-9 are downstream of SMO, it would be especially interesting to evaluate their potency in overcoming resistance observed for SMO inhibitors. Potentially, polymeric nanoparticle encapsulation of HPP-9 could be pursued, as previously reported for HPI-1^27^. While we were focused on the effect of HPP-9 on Hedgehog signal transduction, there may be other interesting applications of this molecule (and the parent HPI-1), especially when targeting BRD2. For example, a recent report has shown that inhibition of BRD2 can block SARS-CoV-2 infection^59^.

In summary, we here show that PROTACs provide a valid methodology for the target deconvolution of small molecules and are a useful addition to the chemical biology toolbox to decipher the target(s) of phenotypic hits. We conclude that HPI-1 inhibits the Hh pathway through the displacement of the BET bromodomains from DNA, opening biological applications for this molecule beyond Hh pathway inhibition. Finally, targeted degradation of BET bromodomain proteins by HPP-9 is long-lasting, providing the unique opportunity of prolonged Hh pathway inhibition. We anticipate that this strategy is widely applicable, with the strong advantage that once the PROTAC is synthesized, it functions directly as a target validation tool, and could provide a promising pharmacological entity in itself.

## Methods

### Chemical synthesis

Synthetic routes and structural characterization data for HPI-1 analogs and HPPs are described in the Supplementary Methods.

### Cell lines

SHH-LIGHT2^36^, SHH-GFP^39^, HEK239T-EcR-ShhN cells, A549, Wnt-LIGHT^20^, and SUFU-KO-LIGHT^28^ cells were provided by James Chen (Stanford University). SHH-LIGHT2 cells were maintained in DMEM (high glucose, Glutamax) containing 10% calf serum (CS), 1% sodium pyruvate, 100 U/mL penicillin, 100 μg/mL streptomycin, 150 μg/mL zeocin, and 400 μg/mL G418. SHH-GFP cells were maintained in DMEM containing 10% CS, 1% sodium pyruvate, 100 U/mL penicillin, 100 μg/mL streptomycin, and 150 μg/mL zeocin. SUFU-KO-LIGHT and Wnt-LIGHT were maintained in DMEM containing 10% fetal bovine serum (FBS), 1% sodium pyruvate, 100 U/mL penicillin, 100 μg/mL streptomycin, and 150 μg/mL zeocin. NIH-3T3 cells were purchased from ATCC (CRL-1658) and maintained in DMEM containing 10% CS, 1% sodium pyruvate, 100 U/mL penicillin, and 100 μg/mL streptomycin. HEK293T cells (ATCC, CRL-3216), A549, Wnt3a-producing L cells (ATCC, CRL-2647), and HEK239T-EcR-ShhN cells were maintained in DMEM containing 10% FBS, 100 U/mL penicillin, and 100 μg/mL streptomycin. IMCD3-FlpIn cells were a gift from David Mick (University of Saarland) and were maintained in DMEM/F12, 10% FBS, 1% glutamine, 100 U/mL penicillin, and 100 μg/mL streptomycin. HeLa cells were a gift from Aurelien Roux (University of Geneva) and maintained in DMEM containing 10% FBS, 100 U/mL penicillin, and 100 μg/mL streptomycin. MB55 and MB56^44^ were a gift from Rosalind Segal, Harvard Medical School, and were cultured as spheroids in 1:1 DMEM:F12, B27 supplement (Gibco), 100 U/mL penicillin, and 100 μg/mL streptomycin. All cells were cultured at 37 °C with 5% CO_2_ and regularly checked for the absence of mycoplasma.

#### Generation of NF-κB-LIGHT cells

An NIH-3T3 NF-κB-luciferase reporter (NF-κB-LIGHT) cell line was generated by stable integration of pGL4.32 plasmid (Promega) after transient transfection using FuGENE® 4 K Transfection Reagent (Promega). NIH-3T3 cells were seeded in a six-well plate to reach 50% confluency by the next day. Cells were transfected with a master mix diluted in OptiMEM (Gibo) composed of 2.5 µg of pGL4.32 plasmid and 7.5 µl of FuGENE® 4 K Transfection Reagent, according to the manufacturer’s protocol. Hygromycin B (Invitrogen) selection (135 µg/mL) was started 48 h after transfection for 14 days. Afterward, the cells were maintained in DMEM containing 10% fetal bovine serum, 60 µg/mL Hygromycin B, 1% sodium pyruvate, 100 U/mL penicillin, and 100 μg/mL streptomycin.

### ShhN and Wnt production and tittering

To produce ShhN, HEK239T-EcR-ShhN cells were grown to 80% confluence, after which the medium was changed to 2% FBS DMEM. To produce Wnt, Wnt3a expressing L cells were grown to 70% confluence, after which the medium was changed to 10% FBS DMEM. The conditioned medium was collected after 48 h and filtered through a 0.22 µm filter device (Corning). The titer of ShhN and Wnt was determined using SHH-LIGHT2 and Wnt-LIGHT luciferase reporter cells, respectively (see Luciferase reporter assays), and a concentration approximately twofold over the minimum dilution needed for full luciferase induction was used for further experiments.

#### Luciferase reporter assays

SUFU-KO-LIGHT cells were seeded in a 96-well plate (30,000 cells/well). The next day, the medium was removed and starvation medium (DMEM w/o phenol red, 0.5% FBS) with probes in different concentrations or an equivalent amount of DMSO vehicle was added and the cells incubated for 16–18 h. SHH-LIGHT2 cells were seeded in a 96-well plate (35,000 cells/well). The next day, the medium was removed and ShhN-containing starvation medium (DMEM w/o phenol red, 0.5% CS) with compounds in the indicated concentrations or an equivalent amount of DMSO vehicle was added and the cells incubated for 28 h. Wnt-LIGHT cells were seeded in a 96-well plate (10,000 cells/well). The next day at 40% confluency, the medium was removed and Wnt-containing starvation medium (DMEM w/o phenol red, 0.5% FBS) with compounds in the indicated concentrations or an equivalent amount of DMSO vehicle was added and the cells incubated for 28 h. NF-κB-LIGHT cells were seeded in a 96-well plate (20,000 cells/well). The next day at 80% confluency, the medium was removed and TNFα-containing starvation medium (DMEM w/o phenol red, 0.5% FBS, 20 ng/mL TNFα) with compounds in the indicated concentrations or an equivalent amount of DMSO vehicle was added and the cells incubated for 20–24 h. After compound incubation for the indicated times, the medium was removed and the cells were lysed for 30 min at rt (12.2 mM Tris pH 7.4, 4% glycerol, 0.5% Triton X-100, 0.5 mg/mL bovine serum albumin (BSA), 1 mM EGTA, 1 mM DTT), and the luminescence was quantified using homemade firefly luciferase reagent (0.025 M di-glycine, 0.015 M K_x_PO_4_ pH = 8, 4 mM EGTA pH = 8, 0.5 mM DTT, 0.015 M MgSO_4_, 2 mM ATP, 25 mM Coenzyme A, and 0.9 μΜ luciferin) on a multi-mode microplate reader GloMax (Promega). Data were normalized to the DMSO with or without stimulation controls and curves were fitted and analyzed to determine the IC_50_ using Prism 9, GraphPad Software, La Jolla, CA.

### Cell viability assays

NIH-3T3, HEK293T, HeLa, and IMCD3 cells were seeded in a 96-well plate (3000 cells/well). After 5 h, the cells were treated with the indicated compound concentrations or DMSO vehicle and incubated for 48 h. MB55 and MB56 cells were seeded in a low-adherent 96-well plate (10,000 cells/well). After 24 h, the cells were treated with the indicated compound concentrations or DMSO vehicle and incubated for 168 h. Cell viability was determined using Celltiter-Blue (Promega) and fluorescence was measured using multi-mode microplate reader GloMax (Promega) using excitation and emission wavelengths of 570 and 600 nm, respectively, or using Celltiter-Glo 3D (Promega) and measuring luminescence (for spheroids). The results were analyzed using nonlinear regression (Prism 9, GraphPad Software, La Jolla, CA).

#### Sample preparation for proteomic analysis

SHH-GFP cells were seeded in 3.5 cm dishes (500,000 cells/dish) and grown to confluency. The next day cells were treated with HPI-1, HPP-9, or hydroxythalidomide (1 μM final) or an equivalent amount of DMSO vehicle (0.1%) in serum starvation medium (DMEM w/o phenol red, 0.5% CS) for 27 h (5 dishes/group). Cells were washed with PBS, trypsinized, and collected by centrifugation (500 × *g*, 7 min). After centrifugation, the cell pellets were washed with PBS (1x), centrifuged (500 × *g*, 7 min), flash frozen with liquid nitrogen, and stored at −80 °C until further processing. Samples were prepared for liquid chromatography/mass spectrometry (LC/MS) using the phase-transfer surfactant method, with minor modifications. First, proteins were extracted from islets and solubilized using a buffer containing 12 mM sodium deoxycholate, 12 mM sodium *N*-dodecanoylsarcosinate, and 100 mM Tris pH 9.0, with EDTA-free Protease Inhibitor Cocktail (Roche, Switzerland). Samples were sonicated for 4 min using a Bandelin Sonorex ultrasonic bath (FAUST) with 20-s on/20-s off cycles. Cell debris was removed after centrifugation at 18,000 × *g* for 20 min at 4 °C. Protein concentrations were adjusted to a uniform concentration for a set of samples (0.5–1.0 μg/μL), and between 5 and 20 μg protein was used for digestion. Cysteine–cysteine disulfide bonds were reduced with 5 mM TCEP at 37 °C for 30 min. Free thiol groups were alkylated with 20 mM iodoacetamide in the dark at room temperature for 30 min. Alkylation reactions were quenched with 75 mM cysteine at room temperature for 10 min. Samples were diluted with 3.1 volumes of 50 mM ammonium bicarbonate. Lysyl endopeptidase (Wako, Japan) and trypsin (Promega, USA) were added at a 100:1 ratio of sample protein:enzyme (w/w) and samples were digested for 16 h at 37 °C. Afterward, 1.77 volumes of ethyl acetate were added, and samples were acidified with trifluoroacetic acid (TFA), which was added to 0.46% (v/v). Following centrifugation at 12,000 × *g* for 5 min at room temperature, samples were separated into two phases. The upper organic phase containing sodium deoxycholate was removed, and the lower aqueous phase containing digested tryptic peptides was dried using a centrifugal vacuum concentrator. Digested peptides were dissolved in 300 μL of 0.1% (v/v) TFA in 3% acetonitrile (v/v). Samples were sonicated for 1 min, centrifuged at 15,000 × *g* for 15 min, and desalted using MonoSpin C18 columns (GL Sciences Inc., Japan). Peptides were eluted from C18 columns using 0.1% TFA in 50% acetonitrile and dried in a vacuum concentrator. Tryptic peptides were dissolved in 0.1% (v/v) formic acid in 2% (v/v) acetonitrile for MS analysis.

#### MS measurements

Samples were measured using an Easy Nano LC - Orbitrap Fusion System (Thermo Fisher Scientific, USA), equipped with a PST The Nimbus ion source (Phoenix s&t). The same amount of peptide was injected for each sample, typically 300–600 ng in a volume of 2 to 5 μL. Peptides were separated on a 3-μm particle, 75-μm inner diameter, 12-cm filling length homemade C18 column. A flow rate of 300 nL/min was used with a 2-h gradient (2–25% solvent B in 122 min, 25–45% solvent B in 4 min, and 45–75% solvent B in 4 min. The gradient was followed with two rounds of washing steps, in each step, the gradient switched to 98% solvent B and kept there for another 5 min, and switched to 2% solvent B in 1 min, and kept there for another 2 min. In the second round of washing, an extra 12 min of 2% solvent B was kept for system equilibration. Solvent A was 0.1% (v/v) formic acid in LC/MS grade water and solvent B was 0.1% (v/v) formic acid in 100% (v/v) acetonitrile. The ion source settings from Tune were used for the mass spectrometer ion source properties.

For data-dependent acquisition (DDA), full MS spectra were acquired from 375 to 1500 m/z at a resolution of 120,000. MS2 spectra were acquired in the ion trap with a rapid scan rate. The default charge state for the MS2 was set to 2. The charge states 2–7 were included for MS2. HCD fragmentation was set to a fixed collision energy of 35%, and dynamic exclusion was set to 30 s. For both full MS and MS2, the AGC target was set to standard with a maximum injection time (IT) set to auto.

For data-independent acquisition (DIA), data were acquired with 1 full MS and 38 overlapping isolation windows constructed covering the precursor mass range of 350–1200 m/z. For full MS, Orbitrap resolution was set to 120,000. The AGC target was set to custom and maximum IT was set to 60 ms. DIA segments were acquired at 30,000 resolution with an AGC target custom and a dynamic maximum IT. HCD fragmentation was set to a normalized collision energy of 27%.

#### Protein identification and quantification

Raw files from DDA measurements were searched against the Uniprot mouse database (20220223_133530_uniprot_Mus + musculus_[10090]_reviewed.bgsfasta) using SpectroMine software (SpectroMine 3, Biognosys, Switzerland) with default settings (minimum peptide length: seven amino acids). Ideal mass tolerances are calculated by SpectroMine to generate the library, so there is no fixed value. Digestion enzyme specificity was set to Trypsin/P. The modification included carbamidomethylation of cysteine as a fixed modification, and oxidation of methionine and acetyl (protein N-terminus) as variable modifications. Up to two missed cleavages were allowed. A decoy database was included to calculate the FDR. Search results were filtered with FDR 0.01 at both peptide and protein levels. Filtered output was used to generate a sample-specific spectral library using Spectronaut software (Spectronaut 15, Biognosys, Switzerland). Raw files from DIA measurements were used for quantitative data extraction with the generated spectral library or using the directDIA workflow. FDR was estimated with the mProphet approach and set to 0.01 at both peptide precursor level and protein level. For two-group comparison, differential abundance testing was performed with an unpaired *t*-test. *Q*-values were the multiple testing corrected *p* values.

#### Docking of HPI-1

The crystal structure of the second bromodomain of BRD2 was downloaded from the Protein Data Bank (PDB code 7OE8^47^). The protein was then prepared with Maestro release 2021-1 (Schrödinger, LLC, New York, NY, 2021). Briefly, hydrogens were added, the H-bond assignment was optimized at pH 7.0 with PROPKA^60^ and a final minimization with the OPLS4^61^ force field was realized. Finally, all water molecules were discarded, and a grid was generated with the Grid Generation utility, centered on the co-crystallized ligand.

The ligand HPI-1 was parameterized with the LigPrep utility with the OPLS4 force field and docked with Glide within Maestro using default settings, that is, standard precision and flexible ligand sampling. The two-dimension interaction diagram was generated by Maestro, and all pictures were rendered with ChimeraX^62^. The docking solution structure has been deposited in the dedicated Zenodo repository with the identifier 10.5281/zenodo.7041023.

#### Generation of ternary complexes

The protocol was adapted from ref. ^49^. Briefly, 5000 binary complexes of the second bromodomain of BRD2 (PDB 7OE8^47^) and cereblon E3 ligase (PDB 5FQD^63^ where thalidomide from PDB 4CI12^64^ replaced lenalidomide) were generated with the Rosetta software package^65^ using a local docking protocol. In both proteins, the ligands (HPI-1 for BRD2 and thalidomide for cereblon) were kept and considered rigidly as part of their respective protein. The first 500 binary complexes that displayed the highest interface score provided by Rosetta were kept for the next step. The linker of the PROTAC and a few atoms from the ligands (stubs) were extracted from HPP-9 and OMEGA 4.1.0.0 (OpenEye Scientific Software, Santa Fe, NM, USA) was used to generate low-energy conformers. We randomly picked 1000 conformers for the next step. Finally, all the linker conformers were overlapped on the binary complexes and ternary complexes were formed when the RMSD between the ligands and the stubs of the linker was less than 0.4 Å. All complexes underwent a minimization and the lowest-energy ternary complex was taken as a physical example of a BRD2-CRBN-PROTAC ternary complex. The picture was rendered with ChimeraX^62^.

#### Unbiased molecular dynamic simulation

The docking solution of HPI-1 in BRD2 was used as the starting structure for the simulation. The ligand HPI-1 was parameterized with the “PARAMETERIZE” module from PlayMolecule^66^, with the ANI-2x mode of calculation. The protein was protonated at pH 7.0 with PROPKA^60^ and parameterized with the DES-amber force field^67^. This force field was published in 2020 and is an improvement of the Amber protein force field, achieving high accuracy in single-chain protein and protein–protein complexes. A total of 8196 TIP4P-D^68^ water solvated a dodecahedral box with a minimum of 10 Å between the protein and the border of the box. Two sodium ions were added to neutralize the system. The resulting system contains 34681 atoms. All subsequent steps were performed with GROMACS 2021.2^69^. A first minimization was performed with the steepest descent minimization until the maximum force reached <500 kJ/mol/nm. We then carried out three independent replicas starting from the same minimized structure. The equilibration consisted of a 2 ns simulation at constant volume (NVT) followed by 4 ns at constant pressure (NPT). The final, unbiased simulation was done for 1 µs in the NPT ensemble using the V-rescale^70^ thermostat and the C-rescale barostat^71^ (accumulated simulation time: 3 µs). We chose not to use any enhanced sampling method because ligand stability is accessible at the microsecond timescale as we do not expect major conformational change when the ligand is already docked. The simulations were visualized with PyMOL^72^ and ChimeraX^62^, the interactions were quantified with the ProLIF 1.1.0 library^73^ and the RMSD was generated with the “rms” module of GROMACS on the whole production run. The production run from each replica started from a different equilibrated structure, but they all show the same ligand–protein interactions pattern and a stable RMSD over time. For a matter of simplicity, we show only the ligand interactions and the RMSD over time for the first replica (replica 0). The parameters of the ligand, the protein, as well as the starting structures, output structures, settings, and trajectories can be found in the dedicated Zenodo repository with the identifier 10.5281/zenodo.7041023.

#### Fluorescence microscopy—Hh pathway-driven GFP reporter

Cells were seeded in 96-well microscopy plates (Ibidi). For dose-response curves, 35,000 cells/well were seeded, to reach confluency the next day. Then, the cells were treated with the indicated compound concentrations (final concentration of DMSO 0.1%) in serum starvation medium (DMEM w/o phenol red, 0.5% CS) containing an appropriate dilution of ShhN-conditioned medium to give full pathway activation. Control wells contained medium with or without ShhN-containing medium plus the same amount of DMSO vehicle. After 24–30 h of stimulation, the medium was removed, and cells were fixed according to the protocol below. For pre-incubation experiments, cells were seeded at 20,000 cells/well, and treated with the indicated compound concentrations (final concentration of DMSO 0.1%) in serum starvation medium (phenol red-free DMEM with 0.5% CS) for 25 h. The compound was removed, and the cells were stimulated with varying dilutions of ShhN-conditioned medium for 27 h, before removing the medium and fixing the cells. Fixation was done with 4% PFA for 7 min at room temperature, followed by PBS washes (3x) and staining with Hoechst (Life Technologies, 1 h, 1:10,000 in PBS). Cells were imaged using a 20x water immersion objective on a high-content confocal microscope (Molecular Devices™ ImageXpress Micro XL).

#### Immunofluorescence imaging

For BRD2/3/4 immunostaining, fixed cells from the fluorescence microscopy experiment were permeabilized with 0.5% Triton X-100 for 5 min at room temperature, washed with 0.1% Triton X-100 in PBS (PBS-T, 3x), and blocked using 1% BSA in PBS-T (blocking solution) for 1 h at room temperature. Cells were incubated with the primary antibody in blocking solution (1:500 rabbit anti-BRD2 (Bethyl Laboratories, A302-583A, lot 6), 1:500 mouse anti-BRD3 (Santa Cruz Biotechnology Inc., 2088C3a, lot H2521) 1:1000 rabbit anti-BRD4 (Bethyl Laboratories, A301-985A, lot 8) overnight at 4 °C, washed 5 × 5 min with PBS-T, and incubated with appropriate secondary antibodies in blocking solution (1:500 Jackson ImmunoResearch) for 1 h at room temperature. Cells were washed 5 × 5 min with PBS-T and once with PBS before being imaged using a 20x water immersion objective on a high-content confocal microscope (Molecular Devices™ ImageXpress Micro XL). For GLI2/GLI3 trafficking studies, cells were grown to confluency, serum-starved for 20 h in the presence of 1 μM compound or DMSO vehicle, followed by 6 h incubation in the presence or absence of the appropriate amount of ShhN-conditioned medium and 1 μM compound or DMSO as indicated. Cells were sequentially fixed with 4% PFA for 10 min at room temperature and ice-cold (−20 °C) methanol for 5 min, before being washed with PBS (3x), and blocked in blocking solution for 1 h at room temperature. Cells were incubated with primary antibody in blocking solution (1:500 mouse anti-gamma tubulin (Sigma Aldrich, GTU-88, T6557), 1:3000 mouse anti-ARL13B (Biolegend, clone N295B/66, lot B323369), 1:500 goat anti-mouse GLI2 (R&D systems, AF3635, lot XUA0320091), 1:500 goat anti-mouse GLI3 (R&D systems, AF3690)) overnight at 4 °C, washed 5 × 5 min with PBS-T, and incubated with appropriate secondary antibodies in blocking solution (1:500 Jackson ImmunoResearch) for 1 h at room temperature. Cells were washed 5 × 5 min with PBS-T and once with PBS before being imaged using a 60x water immersion objective on a high-content confocal microscope (Molecular Devices™ ImageXpress Micro XL). For p65 translocation studies, NIH-3T3 cells were grown to confluency and treated for 30 min with the indicated compounds or DMSO vehicle in the presence of 2.5 ng/mL Il1β or 10 ng/mL TNFα. Cells were fixed with 4% PFA for 12 min, permeabilized with 0.5% Triton X-100 for 5 min at room temperature, washed with 0.1% Triton X-100 in PBS (PBS-T, 3x), and blocked using 1% BSA in PBS-T (blocking solution) for 1 h at room temperature. Cells were incubated with the primary antibody in blocking solution (1:1000 mouse anti-p65, clone L8F6, Cell Signaling Technologies, #6956, lot 9) overnight at 4 °C, washed 5 × 5 min with PBS-T, and incubated with appropriate secondary antibody in blocking solution (1:500 Jackson ImmunoResearch) for 1 h at room temperature. Cells were washed 5 × 5 min with PBS-T and once with PBS before being imaged using a 20x water immersion objective on a high-content confocal microscope (Molecular Devices™ ImageXpress Micro XL).

#### Quantification of fluorescence microscopy images

Image analysis was performed using MetaXpress software (version 6.5.3.247, Molecular Devices, LLC), and a custom Matlab (R2020b, Mathworks) script. Local background subtraction was performed on all images before analysis. For the nuclear signal, the Hoechst stain was used to mask the nuclei, and the signal intensity within the mask was determined for the other channels. Data were plotted and curves fitted using GraphPad Prism 9. Data were normalized to DMSO controls (with and without ShhN). To determine GLI levels at the tip of the primary cilium, the ARL13B channel was used to create a ciliary mask. The ciliary mask was then used to identify and measure the ciliary signals in the other channels. The γ-tubulin signal (as a centriole marker) was used to orient all cilia from base to tip. Each cilium was divided into 10 bins, and the tip fluorescence for GLI2 and GLI3 was defined as the summed fluorescence in the final five bins of each cilium, regardless of length.

#### Western blotting

For signaling assays, SHH-GFP cells were seeded in a 24-well plate and grown until confluency. The growth medium was then replaced with serum starvation medium with or without ShhN-conditioned medium and compounds at the appropriate dilution. After 28 h, cells were lysed in SDS sample buffer (50 mM Tris HCl pH 6.8, 8% v/v glycerol, 2% w/v SDS, 100 mM DTT, and 0.1 mg/mL bromophenol blue), boiled, and sonicated.

For competition assays, SHH-GFP cells were seeded in a 24-well plate and grown until confluency. The growth medium was then replaced with a serum starvation medium containing DMSO or 1 μM HPP-9 plus the indicated concentrations of competitors for 7 h. Cells were washed with PBS, lysed in SDS sample buffer, boiled, and sonicated.

For time-course assays, all wells were changed to serum starvation at the time compound treatment was started for the longest timepoint, and the medium was changed to compound-containing medium at the indicated times, before lysing all wells at the same time. For GLI1/BRD2/3/4 assessment, A549 cells were seeded at 130,000 cells/well in a 24-well plate. MB55 and MB56 cells were seeded in low attachment 24-well plates (200,000 cells/well). The next day, cells were treated with the indicated compounds in serum starvation medium (A549) or growth medium (MB55/MB56) for 30 h. A549 were washed with PBS and lysed in SDS sample buffer, whereas medulloblastoma spheroids were collected, centrifuged (5 min, 700 × *g*), washed with PBS, centrifuged (5 min, 700 × *g*), and lysed in SDS sample buffer.

For phosphorylation studies, SHH-GFP cells were seeded in 24-well plates (130,000 cells/well). The next day, the cells were serum-starved overnight. Then, cells were treated with the indicated compounds for 2.5 h, before being subjected to PDGF (10 ng/mL) or medium control. To assess MZ1 selectivity, SHH-GFP cells were grown to confluency, serum-starved overnight, and treated with different concentrations of MZ1 for 3.5 h.

Samples were loaded onto a 4–15% Criterion TGX Stainfree gel (Bio-Rad), and run for 45 min, 200 V in Tris/Glycine/SDS buffer (Bio-Rad). Gels were irradiated (1 min), and the stain-free imaged before being transferred onto a PVDF membrane using a Transblot Turbo system (Bio-Rad). Membranes were blocked in 5% milk in 0.1% Tween-20 in Tris-buffered saline (TBST) or 1:1 Seablock:PBS (phospho-antibodies) for 1 h at room temperature, and subsequently incubated with the indicated primary antibody in blocking buffer for 16 h at 4 °C. Primary antibodies used: Goat anti-mouse Gli3 (R&D systems, AF3690), 1:200; Mouse anti-Gli1 (Cell signaling, 2643 S, lot 12), 1:1000; Goat anti-mouse Gli2 (R&D systems, AF3635, lot XUA0320091), 1:1000; Mouse anti-vinculin (Proteintech, 66305-1, clone 2B5A7, lot 10016677), 1:5000; Mouse anti-BRD3 (Santa Cruz, sc-81202, lot H2521), 1:200; Rabbit anti-BRD4 (Bethyl laboratories, A301-985A-M, lot 8), 1:1000; Rabbit anti-BRD2 (Bethyl laboratories, A302-583A-T, lot 6), 1:1000; Phospho-ERK1/2 (Proteintech, 28733-1-AP), 1:1000; AKT-phospho-S473 (Proteintech, 66444-1-IG, clone 1C10B8), 1:1000; Karyopherin-beta HRP conjugated (Importin beta (IMPB), Santa Cruz, sc-137016, H7-HRP lot E1519), 1:1000. Membranes were washed (3 × 10 min TBST), incubated with HRP-conjugated secondary antibody, washed again, developed using Supersignal West Atto Maximum Sensitivity Substrate (Thermo Fisher), and imaged on a Fusion FX geldoc (Vilber). Membranes were stripped using Restore Western Blot stripping buffer (Thermo Fisher) and re-probed as described above. Band intensities were determined using Fiji Image J2 (National Institute of Health) on background subtracted images and normalized to total protein loaded using an appropriate housekeeping protein or stain-free total protein.

#### Real-time quantitative PCR

SHH-GFP cells were seeded in a 12-well plate and grown until confluency. The growth medium was then replaced with serum starvation medium with or without ShhN-conditioned medium and compounds or DMSO vehicle at the appropriate dilution. After 24 h, cells were washed with ice-cold PBS and RNA was extracted using the Reliaprep RNA Cell miniprep system (Promega). The obtained RNA was reverse transcribed using the First strand DNA synthesis kit, random primers (Promega). The cDNA was diluted and used with TaqMan Fast Advanced Master Mix (Applied Biosciences) and the appropriate Taqman primers (Life Technologies) in a CFX qPCR thermocycler (Bio-Rad). Cycle numbers were normalized to the housekeeping gene B2m or Gapdh. Taqman primers used: Mm00494654_m1 mGli1; Mm01293117_m1 mGli2; Mm00436026_m1 mPtch1; Mm00437762_m1 mB2m; Mm99999915_g1 Gapdh; Mm01271171_g1 mBrd2

#### RNA-seq and data analysis

SHH-GFP cells were grown to confluency, serum-starved overnight, and then treated with DMSO alone or DMSO, 10 μM HPI-1, 1 μM HPP-9, or 1 μM dBet6 for 8 h (and 24 h exclusively for the HPP-9 sample) in the presence of ShhN in starvation medium (*N* = 2). Cells were washed in PBS and dissociated with trypsin. RNA was isolated from cell pellets using purification columns (RNeasy mini kit, Qiagen). The sequencing libraries were made from 1 μg of RNA using the Collibri 3′ mRNA kit for Illumina sequencing (Invitrogen) and sequenced on an Illumina NovaSeq at 100 bp PE reads. RNA-seq reads were processed using the following script: https://github.com/NLykoskoufis/BraunLabPipeline. In brief, reads were trimmed (cutadapt) and aligned to the mouse genome (mm10) using STAR. Gene count matrixes were obtained from the aligned 3′ reads using featureCounts. Differential gene expression analysis between conditions was made using DESeq2^74^, with significance cut-offs set at fold changes of >1.5 in either direction and adj_*p*val <0.05.

#### Chromatin Immunoprecipitation

SHH-GFP cells were grown to confluency, serum-starved overnight, and then treated with DMSO alone or DMSO, 10 μM HPI-1, 1 μM HPP-9, or 1 μM dBet6 for 8 h (and 24 h exclusively for the HPP-9 sample) in the presence of ShhN in starvation medium (*N* = 2). ChIP experiments were performed as previously described in ref. ^75^. In brief, 30 million SHH-GFP cells were trypsinized for 5 min, washed with PBS, and fixed for 12 min by addition of formaldehyde to a final concentration of 1%. Crosslinking was then quenched with 0.125 M glycine and cells were incubated on ice for 5 min. Crosslinked cells were spun at 800×*g* for 5 min. Nuclei were prepared with 10 mL cell lysis buffer (50 mM HEPES pH 8.0; 140 mM NaCl; 1 mM EDTA; 10% glycerol; 0.5% NP40; 0.25% Triton X-100), then washed in 10 mL rinse buffer (10 mM Tris pH 8.0; 1 mM EDTA; 0.5 mM EGTA; 200 mM NaCl). The chromatin pellets were resuspended in shearing buffer (0.1% SDS, 1 mM EDTA pH 8.0, and 10 mM Tris pH 8.0) and sonicated for four cycles of 30 s using a Bioruptor Pico sonicator (Diagenode). Sonicated chromatin was spun at 10,000×*g* for 5 min, and the supernatant was collected. For ChIP inputs, 25 µL of sheared chromatin was stored at 4 °C. The IP reactions were set up as follows: the sonicated chromatin from 15 million cells was diluted with 0.25 volume of 5×IP buffer (250 mM HEPES, 1.5 M NaCl, 5 mM EDTA pH 8.0, 5% Triton X-100, 0.5% DOC, and 0.5% SDS) and incubated for 12–16 h at 4 °C with 25 μL protein G Dynabeads (Life Technologies) and 5 μg of antibody. The beads were then washed with 1 mL 1×IP buffer, with 1 mL DOC buffer (10 mM Tris pH 8; 0.25 M LiCl; 0.5% NP40; 0.5% DOC; 1 mM EDTA), then eluted in de-crosslinking buffer (0.5% SDS, Proteinase K, TE Buffer) and incubated 2 h at 55 °C then overnight at 65 °C. DNA was purified using MinElute clean-up columns (Qiagen) and resuspended in 25 μL EB solution. Antibodies: Brd2 (A302-583A, Bethyl, lot 6) and Brd4 (A301-985A, Bethyl, lot 8). For ChIP-qPCRs, 1 μL of ChIP DNA was used for each reaction prepared in PowerUp SYBR Green Master Mix (Thermo) with indicated primers and run on a QuantStudio1 thermocycler (Applied Biosystems). For data analysis, ChIP enrichments (bound/input) were normalized to intergenic control primers.

### Reporting summary

Further information on research design is available in the Nature Portfolio Reporting Summary linked to this article.

## Supplementary information

Supplementary Information

Description of Additional Supplementary Files

Supplementary Data 1

Supplementary Data 2

Supplementary Data 3

Reporting Summary

## Data Availability

The raw experimental data that support the findings of this study—including the starting structures, output structures, settings, and trajectories of the MD simulations and the structural model resulting from in silico docking—are available in Zenodo with the identifier 10.5281/zenodo.7041023. The mass spectrometry proteomics data have been deposited to the ProteomeXchange Consortium via the PRIDE partner repository with the dataset identifier PXD036539 and PXD040859. The sequencing data generated and analyzed in this study are available in the Gene Expression Omnibus repository with accession GSE228015. Details on chemical synthesis, analytical spectra, and uncropped western blots are available in the Supplementary Information. The crystal structure used for the docking study was obtained from the PDB with accession code 7OE8 (C-terminal bromodomain of human BRD2). Source data are provided with this paper.

## Source data

Source Data

## Code availability

Custom code for cilium analysis in MATLAB (R2020b, Mathworks) is provided in Zenodo with the identifier 10.5281/zenodo.7041023.
