# Supplementary material for: Targeted protein degradation reveals BET bromodomains as the cellular target of Hedgehog pathway inhibitor-1
Source: Nat Commun. 2023 Jul 1;14:3893. doi: 10.1038/s41467-023-39657-1 (PMC10314895; doi:10.1038/s41467-023-39657-1)
Supplement: Supplementary file 1 — Supplementary Information [file 41467_2023_39657_MOESM1_ESM.pdf]

Supplementary Information

Targeted protein degradation reveals BET bromodomains as the cellular target of Hedgehog Pathway Inhibitor-1

Meropi Bagka[1], Hyeonyi Choi[1], Margaux Héritier[2,3], Hanna Schwaemmle[4], Quentin T. L. Pasquer[1], Simon M. G. Braun[4], Leonardo Scapozza[2,3], Yibo Wu[5], Sascha Hoogendoorn[1*]

Table of Contents

1. Supplementary Figures
2. Supplementary Methods
   - 2.1 General
   - 2.2 Abbreviations
   - 2.3 Synthesis
3. NMR spectra
4. LC-MS spectra
5. Uncropped western blot membranes
6. Supplementary References

1. Supplementary Figures

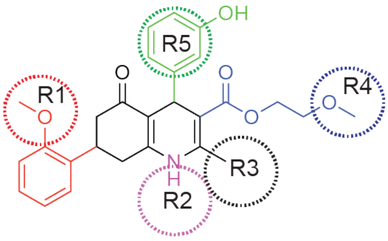

entry	R1	R2	R3	R4	R5	SUFU-KO-LIGHT pIC$_{50}$ (+/- SD)	SHH-LIGHT2 pIC$_{50}$ (+/- SD)
HPI-1	o-OCH_3	H	CH_3	OCH_3	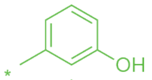	5.9 +/- 0.08 (N=4)	6.0 +/- 0.08 (N=2)
HPI-1-A	o-OH	H	CH_3	OCH_3	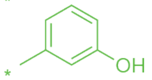	N.A. (N=4)	4.9 +/- 0.24 (N=2)
HPI-1-B	o-O 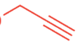	H	CH_3	OCH_3	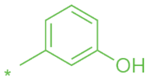	4.4 (N=1)	N.D.
HPI-1-C	o-OCH_3	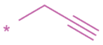	CH_3	OCH_3	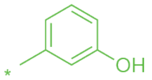	4.8 +/- 0.31 (N=3)	5.5 (N=1)
HPI-1-D	o-OCH_3	H	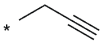	OCH_3	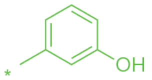	6.0 +/- 0.42 (N=2)	6.2 +/- 0.15 (N=2)
HPI-1-E	o-OCH_3	H	CH_3	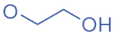	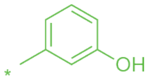	5.8 +/- 0.15 (N=2)	5.6 +/- 0.16 (N=2)
HPI-1-F	o-OCH_3	H	CH_3	OCH_3	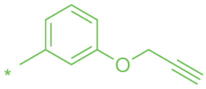	5.7 +/- 0.09 (N=2)	5.9 +/- 0.25 (N=2)

Supplementary Fig. 1. **SAR analysis for HPI-1 analogs**. N.D.: not determined, N.A. not active.

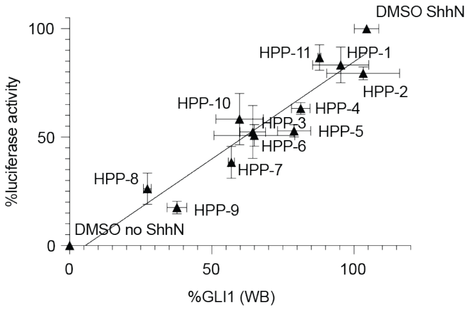

Supplementary Fig. 2. **Comparison between reporter and endogenous readout**. Readout by luciferase assay and GLI1 western blot for the HPP-9 show good correlation (related to data shown in Fig. 1c, d). Mean +/- SD of 4 independent experiments is shown.

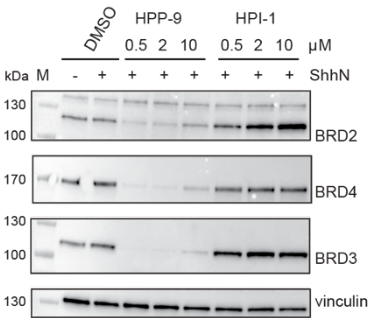

Supplementary Fig. 3. **HPP-9 shows a Hook effect for BET bromodomain degradation.** SHH-GFP cells were treated with various concentrations of HPP-9 or HPI-1 for 27 h and probed for BRD2, BRD3 and BRD4 by western blot. Representative immunoblot of two independent experiments. Related to Fig. 3c.

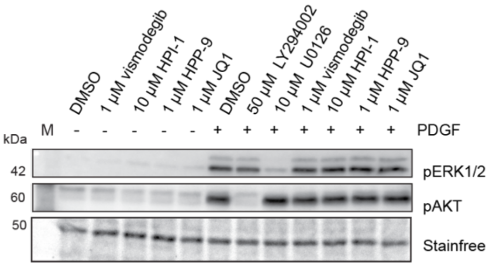

Supplementary Fig. 4. **HPP-9 and HPI-1 do not inhibit PDGF-mediated phosphorylation of pAKT or pERK1/2.** NIH-3T3 cells were treated with PDGF (10 ng/mL) and probed for pERK1/2 and pAKT. Representative immunoblot of two independent experiments.

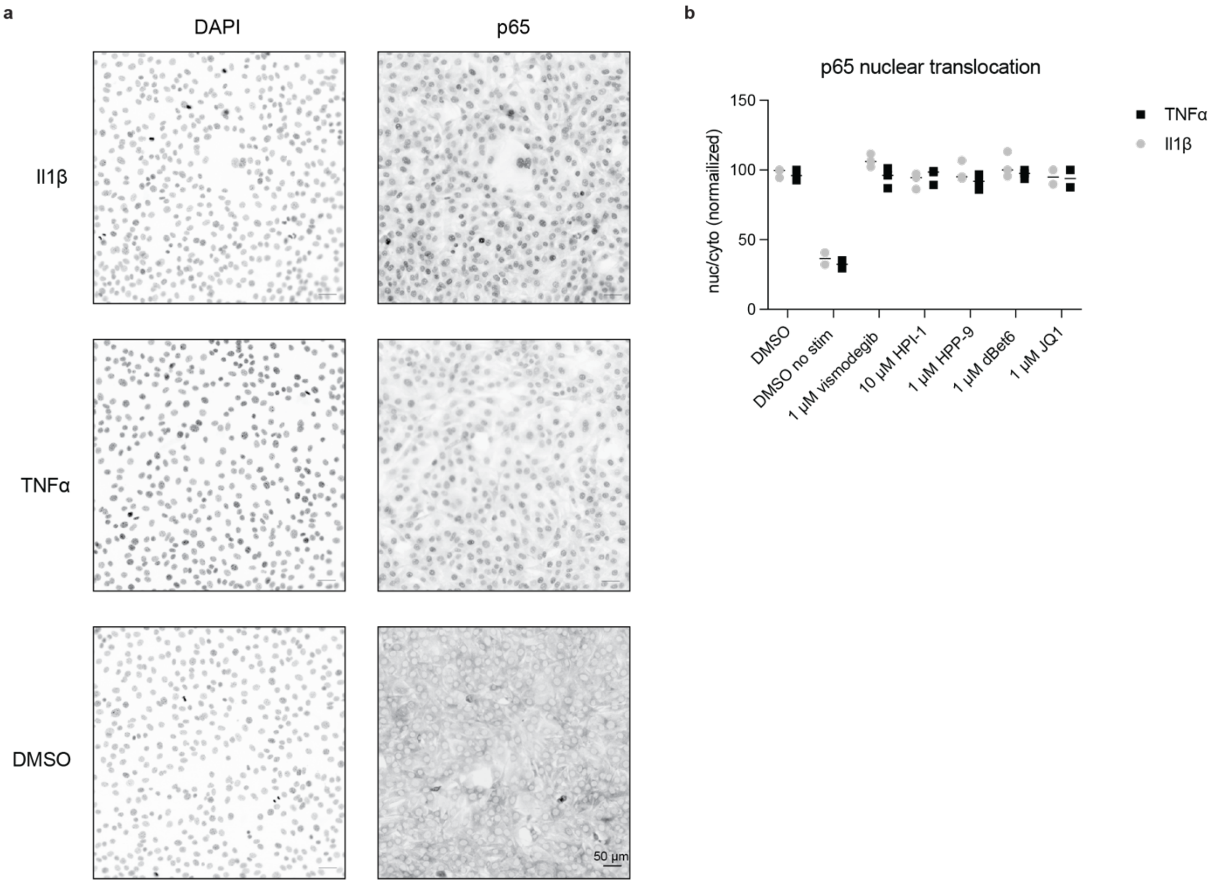

Supplementary Fig. 5. **HPP-9 does not affect p65 nuclear translocation.** NIH-3T3 cells were treated with 2.5 ng/mL Il1β or 10 ng/mL TNFα in the presence or absence of compounds for 30 minutes before being fixed and stained for p65. Representative images are shown in a) and all data for the compounds is quantified in b) N=3 independent experiments, n=16 images analyzed/condition. Line indicates mean.

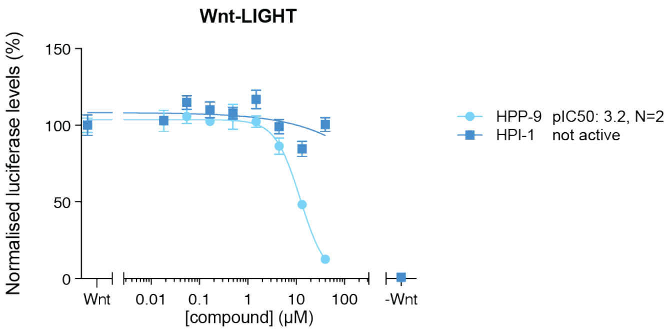

Supplementary Fig. 6. **Inhibitory activity of HPP-9 and HPI-1 in a Wnt-luciferase reporter assay.** Representative curves are shown of two independent experiments, performed in triplicate. Mean +/- SD is plotted.

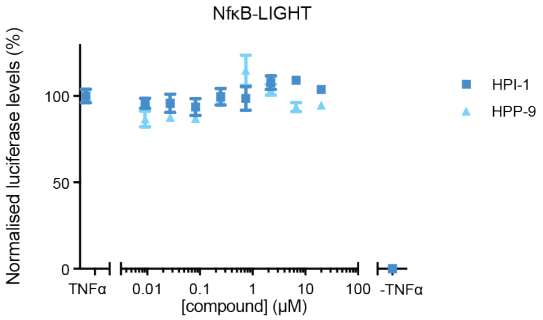

Supplementary Fig. 7. **HPP-9 and HPI-1 do not inhibit NF-kB signaling in a luciferase reporter assay.** A representative curve is shown of 5 independent experiments, performed in triplicate. Mean +/- SEM is plotted.

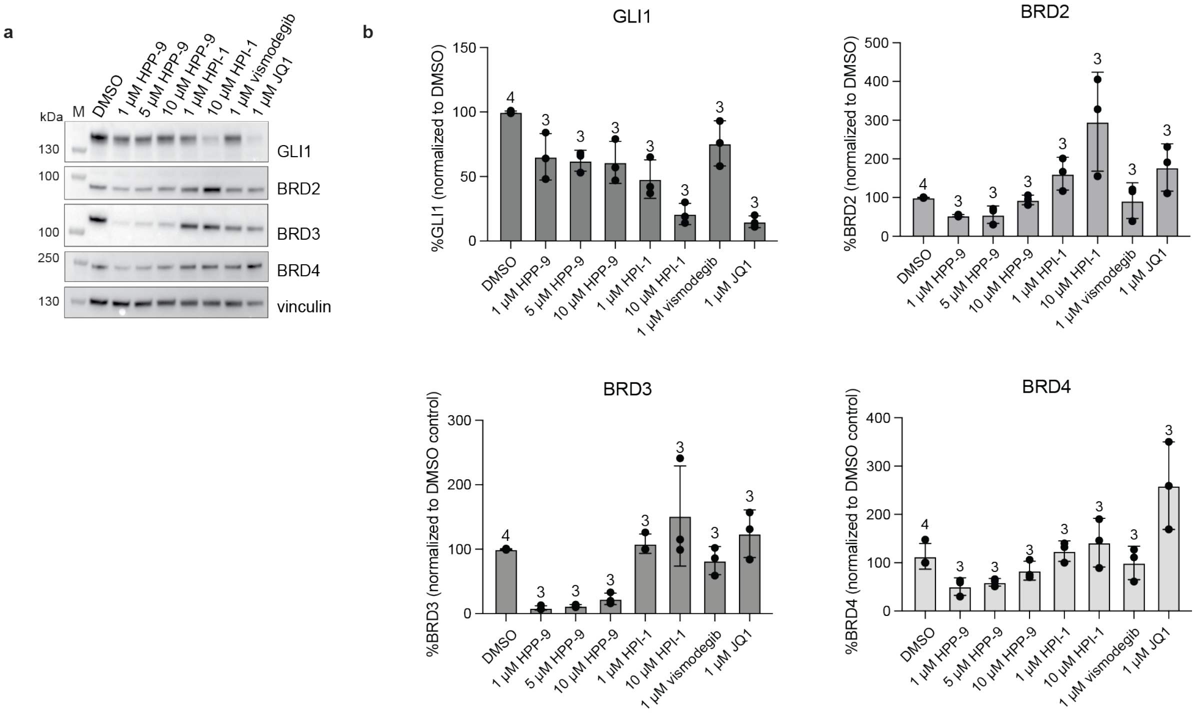

Supplementary Fig. 8. **HPP-9 degrades BET bromodomains in A549 lung adenocarcinoma cells.** Cells were incubated with the indicated compounds for 30 h, before being lysed and resolved on SDS page and probed for the indicated proteins. a) representative immunoblot, b) quantification from N independent experiments as indicated. Mean +/- SD is plotted.

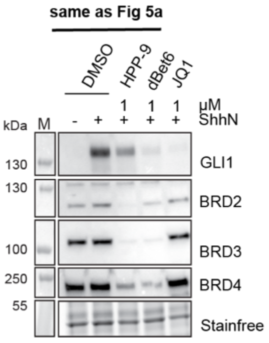

Supplementary Fig. 9. **JQ1 does not increase BRD2 protein levels in NIH-3T3 cells.** SHH-GFP cells were treated with the indicated compounds for 27 h and probed for GLI1, BRD2/3/4. This is the same blot as shown in Fig. 5a, with the inclusion of the JQ1 lane.

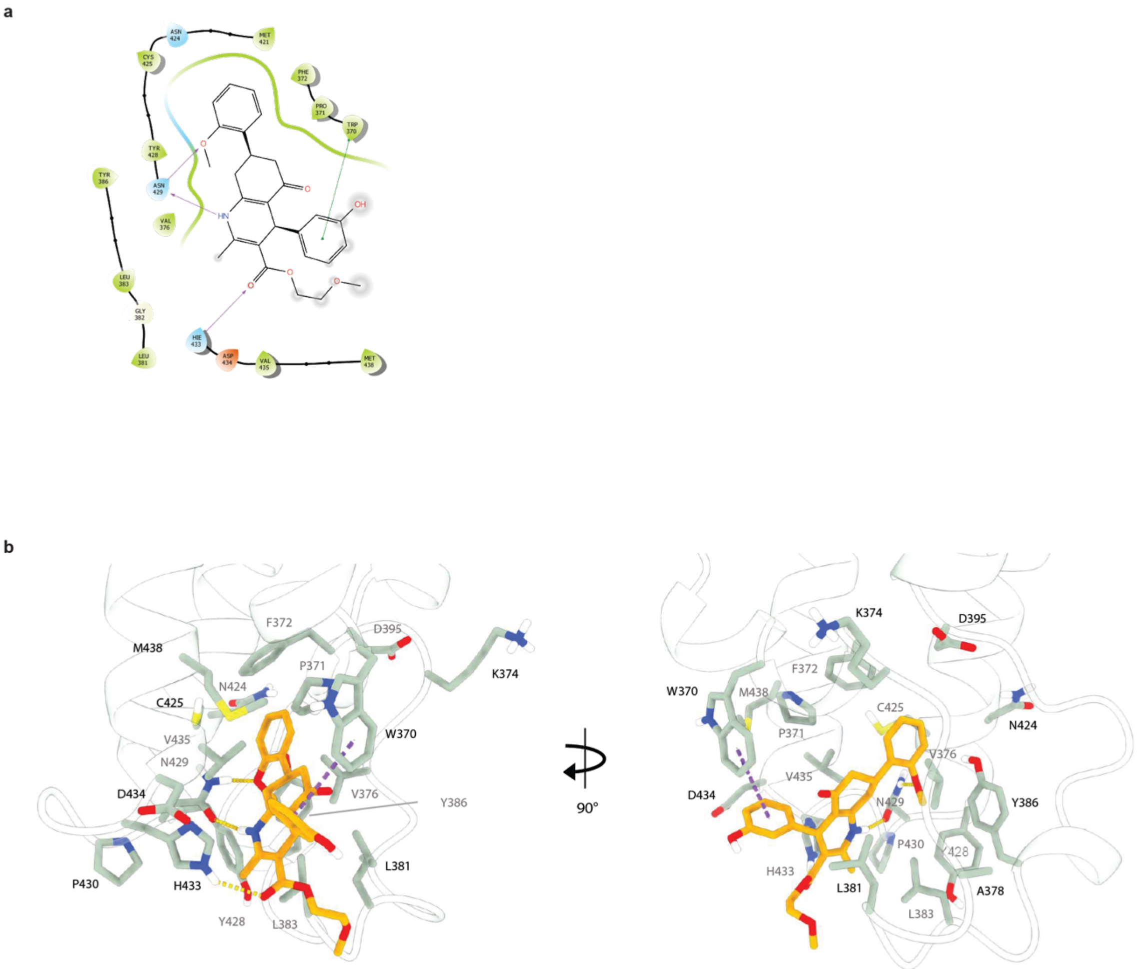

Supplementary Fig. 10. **Proposed model for HPI-1 binding to BRD2(2).** a) 2D diagram of the proposed binding mode for HPI-1, showing the interactions with the amino acids forming the binding site. H-bonds are shown in purple lines while the green line represent π- π stacking. The grey circles show the solvent exposure and the residues in green the hydrophobic environment. The diagram was generated by Maestro (Schrödinger Release 2021-**1,** Schrödinger, LLC, New York, NY, 2021.) b) 3D interactions of

the proposed binding mode with all residues lining the binding pocket in licorice. The images were obtained with UCSF ChimeraX.

HPI-1 (8)

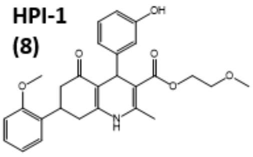

1.3 1.5

19

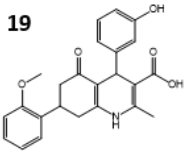

>20 >20

20

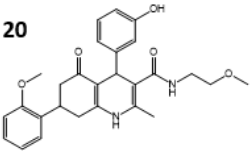

13 7.6

21

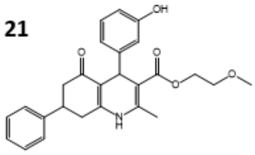

3.2 NA

22

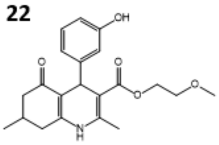

>20 NA

23

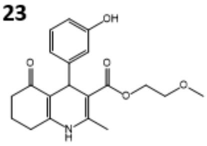

>20 NA

24

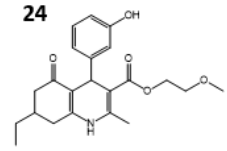

>20 >20

25

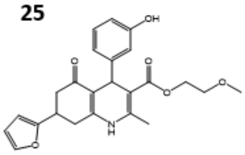

>20 >20

26

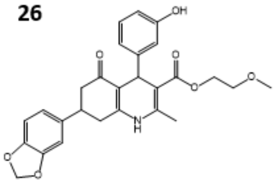

4.8 5.3

27

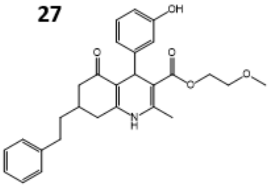

7.2 6.1

28

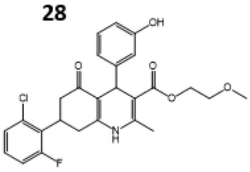

8.1 9.4

29

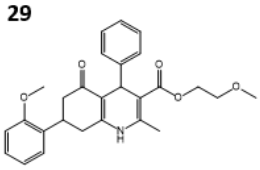

0.98 NA

30

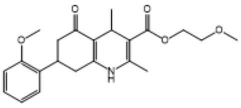

13 NA

31

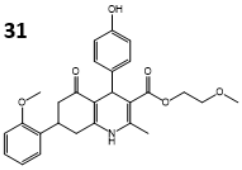

1.5 1.7

32

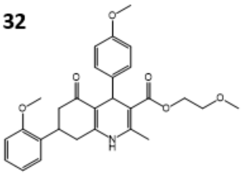

2.3 3.2

33

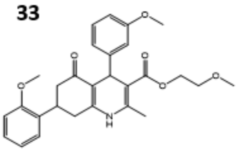

0.67 0.71

34

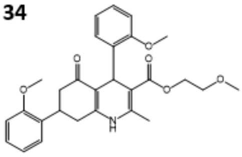

1.1 1.2

35

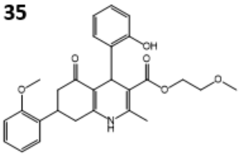

0.76 2.8

36

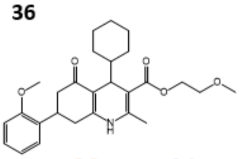

3.0 2.1

37

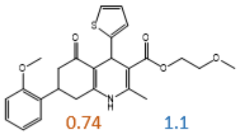

0.74 1.1

38

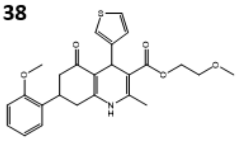

1.2 1.1

39

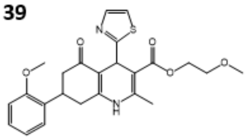

2.9 2.9

40

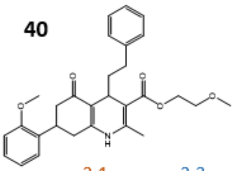

2.1 2.3

41

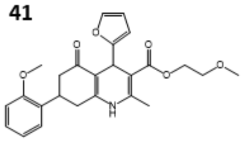

1.3 2.0

42

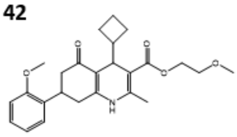

6.3 7.8

43

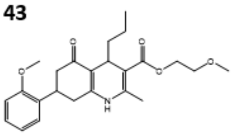

3.3 3.9

44

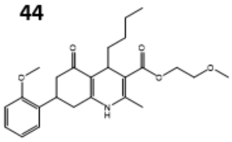

3.8 7.9

45

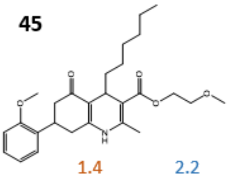

1.4 2.2

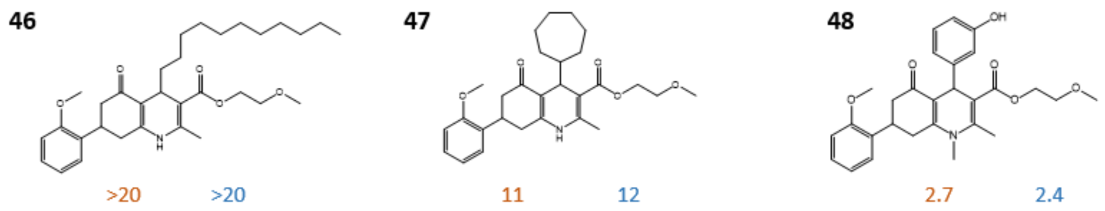

Supplementary Fig. 11. Structures of HPI-1 analogs described by the patent of Chen et al. [1] and their IC_{50} (µM) on Shh-LIGHT2 cells stimulated with ShhN (in red) or SAG (in blue).

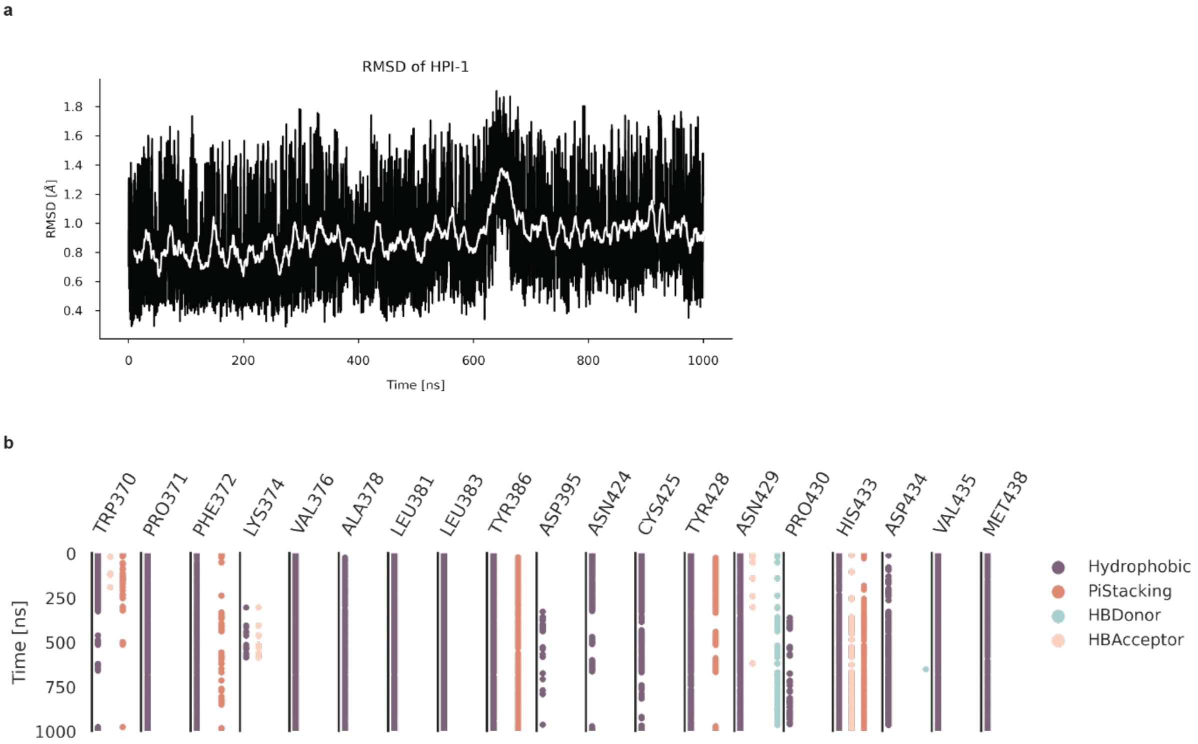

Supplementary Fig. 12. **RMSD of HPI-1 binding to BRD2(2).** a) Ligand stability assessed by its RMSD over the trajectory. The fluctuations are shown in black and the rolling average over 10 ns is in white b) HPI-1 interactions with the binding site residues along the MD trajectory.

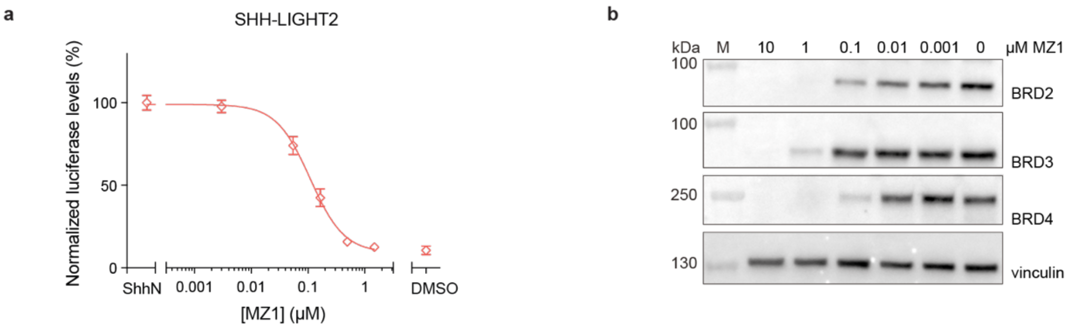

Supplementary Fig. 13. **MZ1 is a potent Hedgehog pathway inhibitor with limited selectivity in NIH-3T3 cells.** a) Representative dose-response curve in SHH-LIGHT2 cells. Three independent experiments. Mean +/- SD is plotted. b) SHH-GFP cells were treated with the indicated concentrations of MZ1 for 3.5 h, before being lysed and probed for BRD2/3/4.

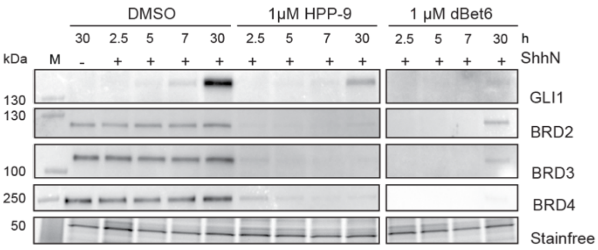

Supplementary Fig. 14. **dBet6 and HPP-9 degrade BET bromodomains at different speed.** SHH-GFP cells were treated with 1 µM of HPP-9 or dBet6 for the indicated times and lysates were probed for GLI1, BRD2, BRD3 and BRD4 by WB. Representative blot of two independent experiments is shown.

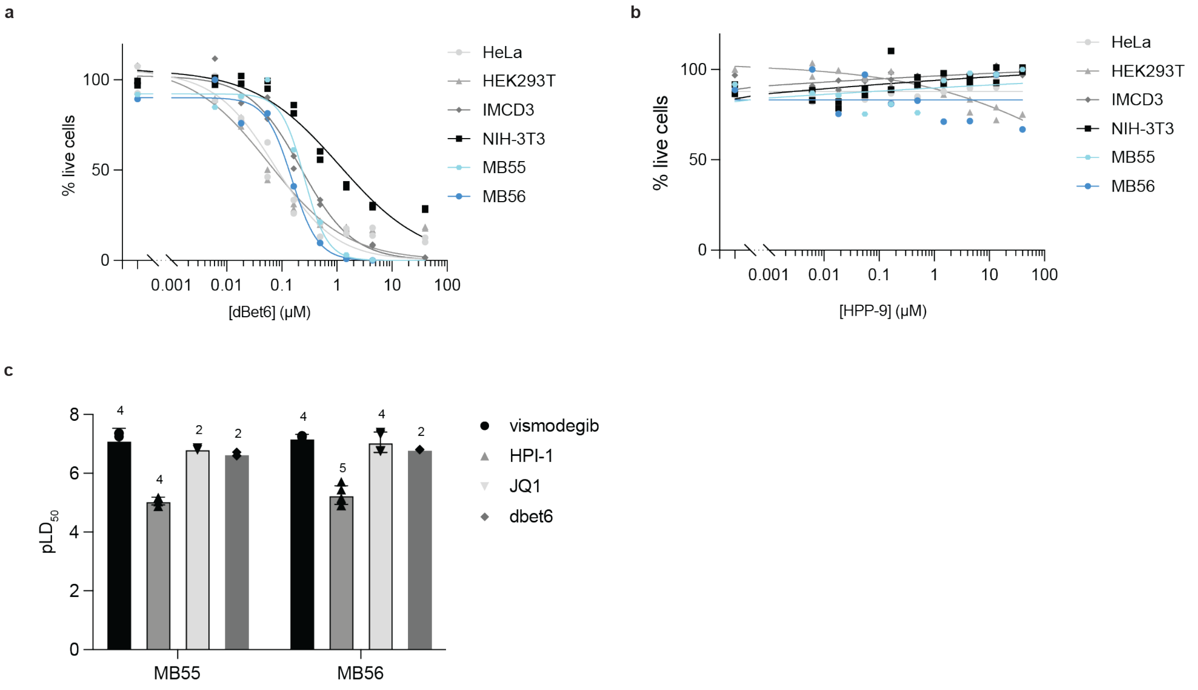

Supplementary Fig. 15. **dBet6 shows much higher toxicity than HPP-9.** The indicated cell lines were incubated with varying concentrations of a) dBet6 or b) HPP-9 and cell viability was measured 48 h later, or, for MB55/MB56 168 h later. Representative curves of 2 independent experiments are shown, performed in duplicate. Individual values are plotted as symbols. c) pLD_{50} values for the indicated compounds in MB55 and MB56 spheroid cell viability assays. N independent experiments as indicated above the bars. Mean +/- SD is plotted when N>3.

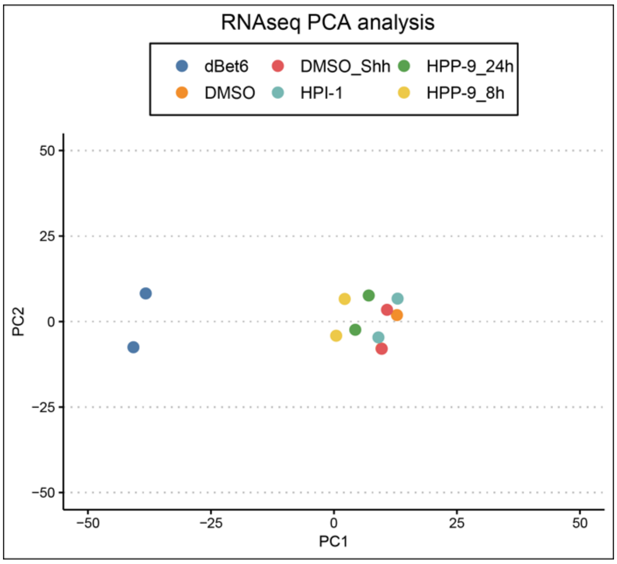

Supplementary Fig. 16. **Principal component analysis of RNAseq experiments**. Principal component analysis of replicate RNA seq experiments shows good agreement between replicates and highlights major changes in gene expression induced by dBet6 treatment.

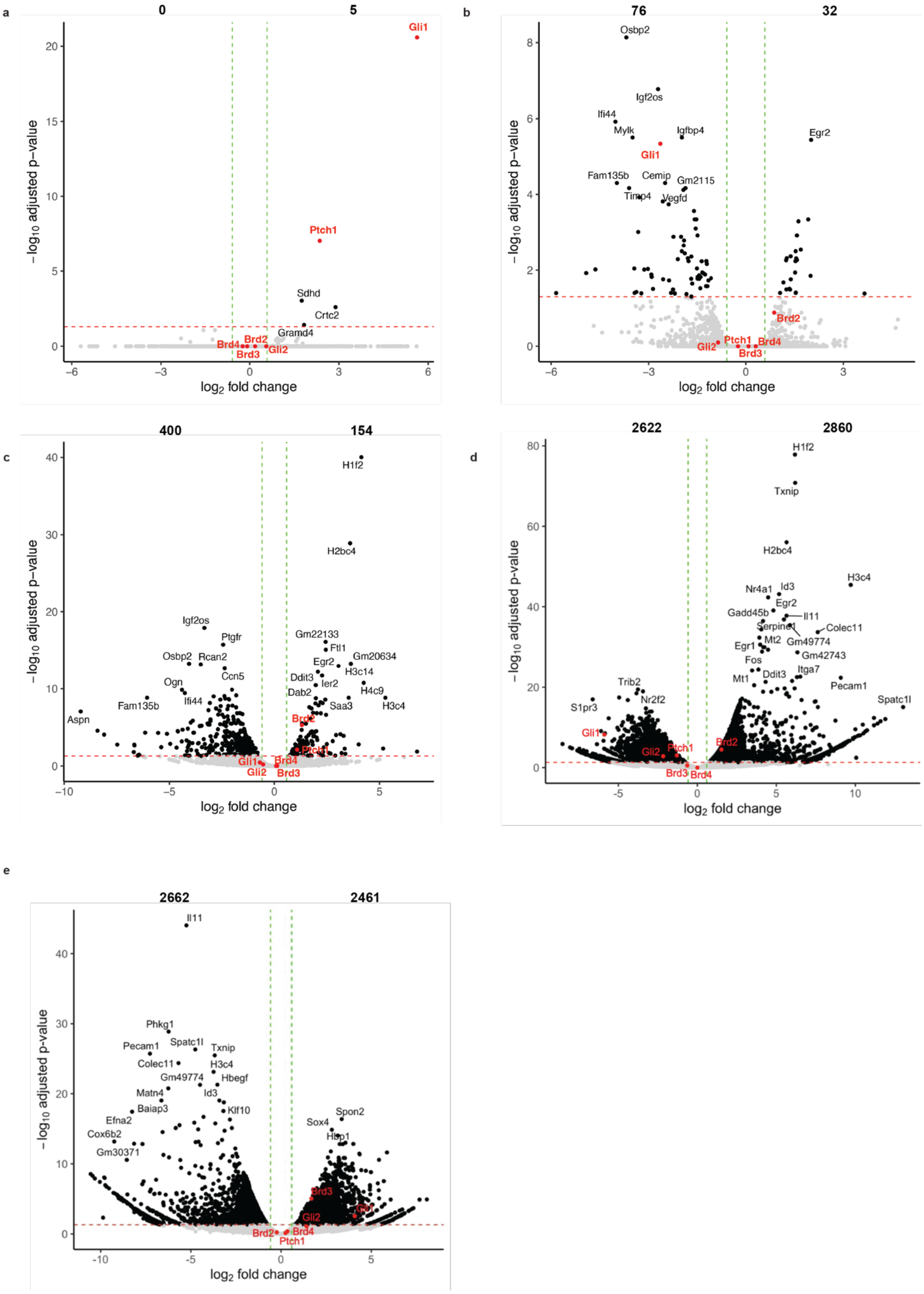

Supplementary Fig. 17. **RNAseq analysis reveals large differences between compound treatments.** Volcano plots showing differentially expressed genes in SHH-GFP cells treated with DMSO alone or

DMSO, 10 µM HPI-1, 1 µM HPP-9 or 1 µM dBet6 for 8 hours (and 24 h exclusively for the HPP-9 sample) in the presence of ShhN. Two independent experiments, padj < 0.05 and 1.5x fold change. a) DMSO vs DMSO + ShhN; b) DMSO + ShhN vs HPI-1 + ShhN; c) DMSO + ShhN vs HPP-9-24h + ShhN; d) DMSO + ShhN vs dBet6 + ShhN; e) dBet6 + ShhN vs HPP-9-8h + ShhN. Related to Fig. 5f. Exact p values can be found in Supplementary Data 3 and were determined using the default settings of DeSeq2, which uses the Wald test and for the adjusted p (padj) values, that correct for multiple testing, the Benjamin-Hochberg correction was performed.

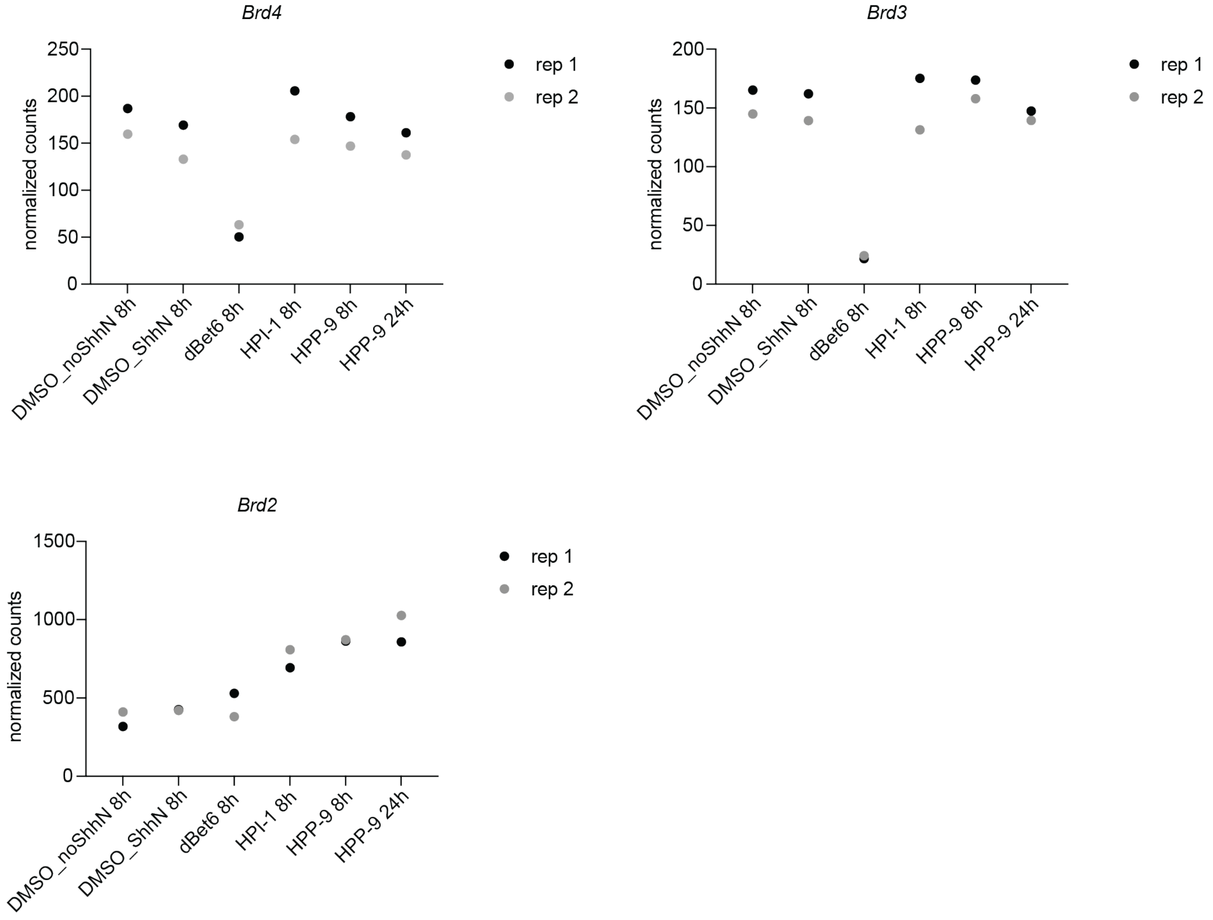

Supplementary Fig. 18. **Effects of compounds on *Brd2/3/4* transcript levels.** RNAseq analysis of SHH-GFP cells that were treated with DMSO, 1 µM dBet6, 1 µM HPP-9 or 10 µM HPI-1 in the presence of ShhN for 8 h (and 24 h for HPP-9). Normalized counts (cpm) for two independent experiments are shown.

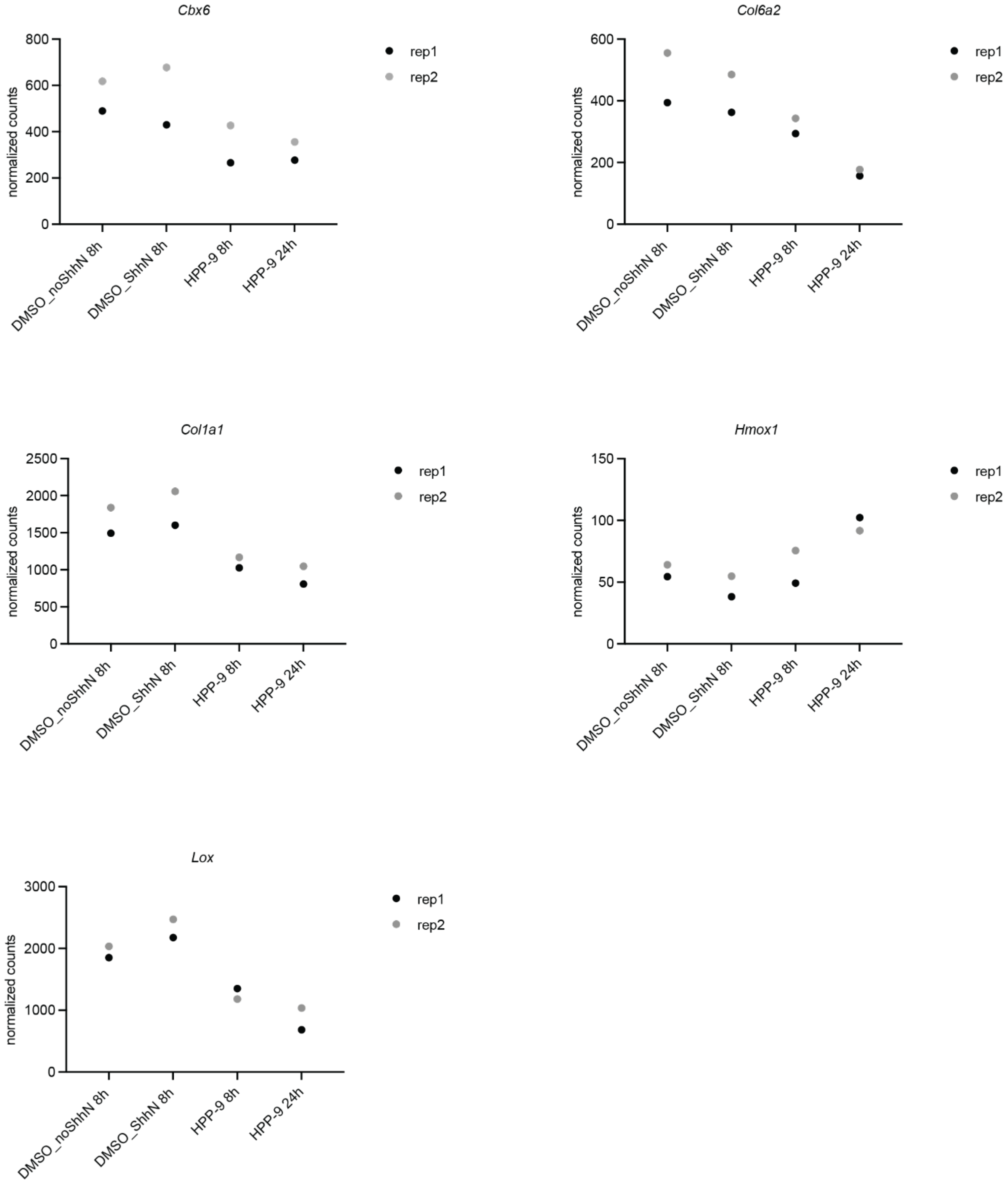

Supplementary Fig. 19. **Effect of HPP-9 on transcript levels of proteomic hits.** RNAseq analysis of SHH-GFP cells that were treated with DMSO or 1 µM HPP-9 in the presence of ShhN for 8 h or 24 h. Normalized counts (cpm) for two independent experiments are shown.

2. Supplementary Methods

2.1 General

Briefly, reagents for synthesis were purchased from Fluka, Sigma-Aldrich, TCI and Acros. Salts of the best grade available from Fluka or Sigma-Aldrich were used as received. Column chromatography was carried out on silica gel 60 (SilicaFlash P60, 40-63 µm). Analytical (TLC) and preparative thin layer chromatography (PTLC) were performed on silica gel 60 (Merck, 0.2 mm) and silica gel GF (SiliCycle, 1 or 0.25 mm), respectively. Reverse phase flash chromatography was performed on a Biotage® Isolera Spektra using pre-packed 12 g Biotage® SNAP Ultra C18 cartridge. LCMS were recorded using a Thermo Scientific Accela HPLC equipped with a Thermo C18 Hypersil GOLD column (50 ´ 2.1 mm, 1.9 µm particles size) coupled with a LCQ Fleet three-dimensional ion trap mass spectrometer (ESI, Thermo Scientific) with a linear elution gradient from 95% H_2O / 5% CH_3CN + 0.1% TFA to 10% H_2O / 90% CH_3CN + 0.1% TFA in 4.0 minutes at a flow rate of 0.75 mL/min (B5), 70% H_2O / 30% CH_3CN + 0.1% TFA to 10% H_2O / 90% CH_3CN + 0.1% TFA in 4.0 minutes at a flow rate of 0.75 mL/min (B30) or 40% H_2O / 60% CH_3CN + 0.1% TFA to 10% H_2O / 90% CH_3CN + 0.1% TFA in 4.0 minutes at a flow rate of 0.75 mL/min (B60). All ^{1}H and ^{13}C NMR spectra were recorded (as indicated) on a Bruker 300 MHz, 400 MHz, or 500 MHz spectrometer at room temperature (25 °C) and are reported as chemical shifts (δ) in ppm relative to TMS (δ = 0). Spin multiplicities are reported as a singlet (s), doublet (d), triplet (t), quartet (q), and quintet (p) with coupling constants (J) given in Hz, or multiplet (m). Broad peaks are marked as br. ^{1}H and ^{13}C resonances were assigned with the aid of additional information from 1D and 2D NMR spectra (H,H-COSY, DEPT 135, HSQC and HMBC). Diastereomeric pair ratios were determined by integration of at least two pairs of signals corresponding to the two diastereomeric pairs present in the sample (inset). Reverse phase HPLC purification was performed using an Agilent Technologies 1260 infinity HPLC equipped with a BSE1ICO-2520 Scorpius-C18e-HP column, 100 Å, 5 µm, 21.2 × 250 mm. Samples were analyzed at the Chemical Biology Mass Spectrometry core facility (ChemBioMS, University of Geneva) by chip-based nanospray infusion using a TriVersa NanoMate (Advion Interchim Scientific, Harlow, UK) hyphenated to a high resolution Q Exactive Plus hybrid quadrupole-orbitrap mass spectrometer (ThermoFisher Scientific, Reinach, Switzerland). Analyte was dissolved in 50% CH_3OH / 50% CH_3CN (10 mM final concentration) and 5 µL were infused at a chip voltage of 1.3kV with 0.3 bar N2 backpressure. The inlet capillary temperature was 100°C and S-lens RF level was 40. Full scan HRMS acquisition was performed over *m/z* 300-2200 at 140k resolution with maximum injection time of 50ms and AGC parameter of 1e6. Acquisition software was ChipSoft v.8.3.1 (Advion) for controlling the TriVersa NanoMate and Xcalibur v.4.4 (Thermo) for the mass spectrometer. MS data was processed with FreeStyle software v.1.7 (Thermo). All mass data are reported as mass-per-charge ratio m/z (intensity in %, [assignment]).

2.2 Abbreviations.

$BMIMBF_4$: 1-Butyl-3-methyl-imidazolium-tetrafluoroborate; DIAD: Diisopropyl azodicarboxylate; DIPEA: N,N-Diisopropylethylamine; DMF: Dimethylformamide; DMSO: Dimethyl sulfoxide; HATU: Hexafluorophosphate azabenzotriazole tetramethyl uronium; HBTU: 3 [Bis (dimethylamino) methyliumyl]-3 *H* -benzotriazole-1-oxide hexafluorophosphate; n-Buli: n-Butylithium; PPh_3: Triphenylphosphine; rt: Room temperature; TBAF: Tetra-n-butylammonium fluoride; TBSMSCl: Tert-Butyldimethylsilyl chloride TBTA: Tris((1- benzyl-1H-1,2,3-triazol-4-yl)methyl)amine; TEA: Triethylamine; TFA: Trifluoroacetic acid; THF: Tetrahydrofuran; TsCl: 4-Toluenesulfonyl chloride.

2.3 Synthesis

Multicomponent Hantzsch reaction

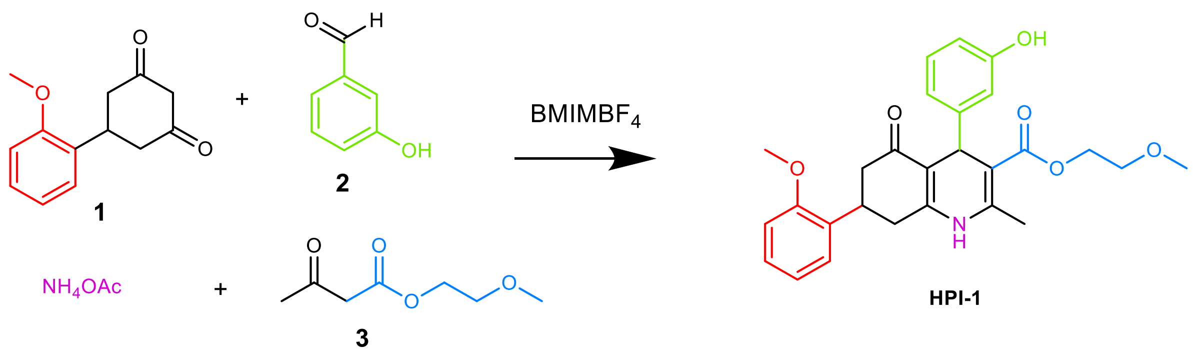

Supplementary Fig. 20. Synthesis of **HPI-1** through Hantzsch reaction.

Hantzsch reaction building blocks

Compound 1 was prepared following previously reported procedures[2]

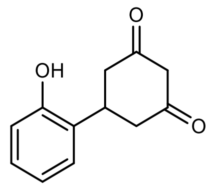

4

Compound 4. In a tri-necked flask the dione **1** (500 mg, 2.3 mmol, 1 eq.) was added and then the system was put under N_2. Next, it was dissolved in anhydrous CH_2Cl_2 (32 ml) and cooled to 0 °C. A 1.0 M BBr_3 solution in CH_2Cl_2 (6.9 ml, 6.9 mmol, 3 eq.) was added dropwise and slowly and the resulting mixture stirred for two days at rt. The reaction mixture was quenched with cold water and the forming

precipitate was filtered and dried in a Büchner funnel to give **4** as a white powder (130 mg, 28%). ^{1}H NMR (400 MHz, CD_2Cl_2): δ 6.97-7.09 (m, 2H), 6.68-6.81 (m, 2H), 3.59-3.65 (m, 1H), 3.27 (s, 2H), 2.62-2.72 (m, 2H), 2.44-2.54 (m, 2H) ppm.

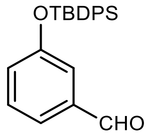

5

Compound 5. Tert-butyldiphenylsilyl chloride (0.56 mL, 2.4 mmol) was added dropwise to a stirred solution of 3-hydroxybenzaldehyde (300 mg, 2 mmol) and imidazole (300 mg, 4.4 mmol) in DMF (1 mL) at room temperature. The reaction mixture was stirred for 10 h. Subsequently, water (2 mL) was added and the resulting mixture was extracted with diethyl ether (5 mL) three times. Combined organic layers were dried over anhydrous $MgSO_4$ and concentrated under high vacuum. The crude product was purified by column chromatography on silica gel (Pentane/EtOAc, 10:1, R_f=0.45) to afford the aldehyde **5** as a colorless oil (690 mg, 96% yield). ^{1}H NMR (400 MHz, $CDCl_3$): δ 9.82 (s, 1H), 7.69-7.73 (d, 4H), 7.40-7.47 (m, 7H), 7.35-7.39 (m, 1H), 7.19-7.30 (m, 1H), 6.93-6.97 (dd, 1H), 1.12 (s, 9H) ppm.

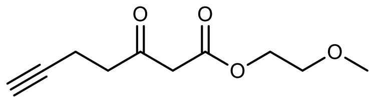

6

Compound 6. A heterogeneous mixture of NaH (60% suspension in mineral oil) (420 mg, 10.5 mmol, 2.1 eq) in dry THF (8 mL) at 0 °C was slowly added to 2-methoxyethyl acetoacetate (0.8 mL, 5 mmol, 1.0 eq). After 30 min the yellow heterogeneous mixture was added to the solution of n-BuLi (1.6 M in hexane) (3.75 mL, 6 mmol, 1.2 eq) at 0 °C and the resulting deep red-orange heterogeneous mixture was allowed to stir for an additional 30 min before it was cooled to -78 °C. Propargyl bromide (0.7 mL, 6.5 mmol, 1.3 eq) was then added slowly to the reaction mixture. The reaction continued to stir for 2 h before it was quenched by slow addition of water. The mixture was extracted with EtOAc and the combined organic phases were washed with sat. aq. NaCl and dried over anh. Na_2SO_4, concentrated under reduced pressure and purified by silica gel column chromatography (Petroleum ether/EtOAc 2.5:1, R_f=0.38) to yield the desired product as a pale-yellow oil (400 mg, 40 %). ^{1}H NMR (400 MHz, $CDCl_3$) δ 4.30 (dd, J = 5.5, 3.7 Hz, 2H), 3.61 (dd, J = 5.6, 3.9 Hz, 2H), 3.52 (s, 2H), 3.38 (s, 3H), 2.82 (t, J = 7.5 Hz, 2H), 2.47 (td, J = 7.4, 2.5 Hz, 2H), 1.96 (q, J = 2.7, 2.2 Hz, 1H).

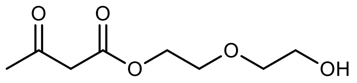

7

Compound 7. A solution of diethylene glycol (743 mg, 7 mmol) and 2,2,6-trimethyl-4H-l,3-dioxin-4-one (1 g,7 mmol) in 1.4 mL of xylene was placed in a 50-mL Erlenmeyer flask. The flask was immersed in an oil bath that had been preheated to 150 °C, and the solution was vigorously stirred. The evolution of acetone became apparent within several minutes, heating was continued for a total of 30 min. The reaction was cooled, the xylene was removed in vacuo. Purification by silica gel column chromatography (CH_2Cl_2/MeOH 100:1, R_f =0.44) to afford compound **7** as a yellow oil. ^1H NMR (400 MHz, $CDCl_3$) δ 4.35 – 4.30 (m, 2H), 3.76 – 3.70 (m, 4H), 3.62 – 3.58 (m, 2H), 3.49 (d, *J* = 3.4 Hz, 2H), 2.27 (s, 3H), 1.91 (s, 1H).

HPI-1 (General procedure for Hantzsch reaction)[2]**. HPI-1.** To a dry round-bottom flask was added **1** (0.100 g, 0.460 mmol), **2** (56 mg, 0.46 mmol), **3** (73 mg, 0.46 mmol), ammonium acetate (35 mg, 0.46 mmol), and the ionic liquid $BMIMBF_4$ (10 µL). The mixture was stirred for 15 min at 90 °C, after which it was cooled to rt and directly purified by silica gel column chromatography (Pentane/EtOAc 3:7, R_f = 0.40) to give **HPI-1** as a pale-yellow solid (140 mg, 66%). ^1H NMR (400 MHz, $CDCl_3$): δ 7.22 − 6.61 (m, 8H), 6.21 (br, 1H, NH), 5.14 (s, 1H), 4.25 − 4.13 (m, 2H), 3.79 (s, 3H), 3.64 − 3.57 (m, 1H), 3.56 − 3.53 (m, 2H), 3.32 (s, 3H), 2.77 − 2.54 (m, 4H), 2.37 (s, 3H). ^{13}C NMR (101 MHz, $CDCl_3$): δ 196.2, 167.3, 157.1, 155.7, 150.4, 148.3, 143.9, 130.2, 129.1, 128.1, 127.1, 120.7, 120.1, 115.3, 113.3, 112.6, 110.7, 105.9, 70.5, 62.8, 58.7, 55.2, 42.2, 36.2, 33.2, 33.0, 19.5. LC-MS (ES^+): m/z 464.09 $[M+H]^+$.

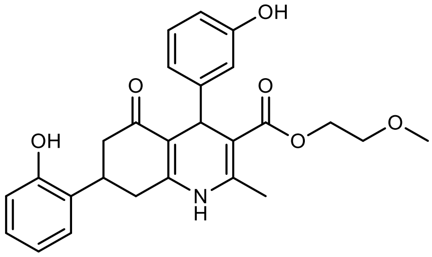

HPI-1-A

HPI-1-A was synthesized following the general procedure for Hantzsch reaction, engaging **2** (33 mg, 0.22 mmol), **3** (35 mg, 0.22 mmol), **4** (45 mg, 0.22 mmol), ammonium acetate (17 mg, 0.22 mmol), and the ionic liquid $BMIMBF_4$ (5 µL). The resulting crude was purified by column chromatography (Pentane/EtOAc 1:1, R_f =0.3) to yield **HPI-1-A** as a yellow powder (33 mg, 33%). ^1H NMR (400 MHz, MeOD and $CDCl_3$): δ 6.92-6.38 (m,8H), 5.14 (s, 1H), 4.18 (m, 2H), 3.80 (s, 3H), 3.55(m, 2H), 3.32 (s, 3H), 2.56-2.47 (m, 4H), 2.16 (s,2H) ppm. LC-MS (ES^+): m/z 450.08 $[M+H]^+$.

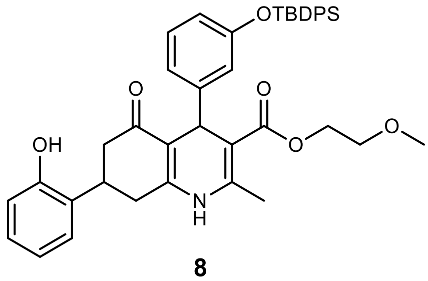

8

Compound 8 was synthesised following the general procedure for Hantzsch reaction, engaging **5** (80 mg, 0.22 mmol), **3** (35 mg, 0.22 mmol), **4** (45 mg, 0.22 mmol), ammonium acetate (17 mg, 0.22 mmol), and the ionic liquid BMIMBF$_4$ (5 µL). The resulting crude was purified by column chromatography (Pentane/EtOAc 1:1, R_f =0.45) to yield **8** as a yellow powder (30 mg, 20%). LC-MS (ES$^+$): m/z 688.17 [M+H]$^+$.

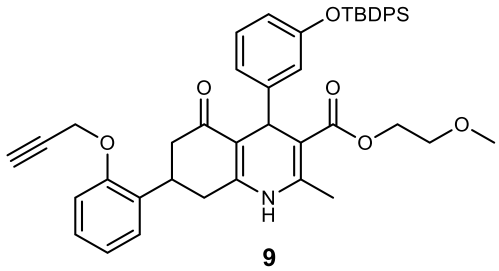

9

Compound 9. Compound **8** (30 mg, 0.044 mmol) was dissolved in 5 ml of acetone and 1.4 eq of anhydrous potassium carbonate (8.5 mg, 0.061 mmol) was added and refluxed for 45 min. Then 2 eq of propargyl bromide (0.009 mL, 0.088 mmol) were added to the mixture and refluxed for 16 h. The reaction was monitored by LC-MS until the consumption of the starting material. The solvent was evaporated in vacuo and the residue redissolved in EtOAc and washed with water 3 times and once with brine to yield **9** as a brown oil (28 mg, 89%). LC-MS (ES$^+$): m/z 726.22 [M+H]$^+$.

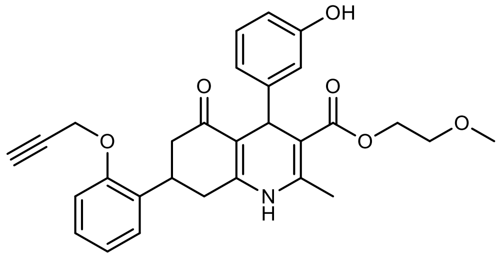

HPI-1-B

HPI-1-B. **9** (28 mg, 0.039 mmol, 1.0 eq) was dissolved in THF (0.5 mL). To this solution was added 1.0 M TBAF (0.077 mL, 0.077 mmol in THF, 2.0 eq) and the mixture was stirred at 40 °C overnight under an argon atmosphere. The resulting solution was concentrated in vacuo and the resulting oil was taken up in EtOAc. The solution was washed with water (3x), washed with saturated NH_4Cl (5x) to remove the salts, dried with $MgSO_4$, and concentrated in vacuo. The resulting crude was purified by column chromatography (CH_2Cl_2/MeOH 15:1, R_f =0.34) to give **HPI-1-B** as a yellow oil (10 mg, 53%). ^{1}H NMR (400 MHz, $CDCl_3$) δ 7.25 – 7.15 (m, 1H), 7.11 (dd, *J* = 7.7, 1.7 Hz, 1H), 7.07 – 6.92 (m, 3H), 6.91 – 6.79 (m, 2H), 6.67 (s, 1H), 6.60 (ddd, *J* = 8.1, 2.6, 1.0 Hz, 1H), 6.53 – 6.48 (m, 1H), 5.11 (d, *J* = 15.6 Hz, 1H), 4.67 (q, *J* = 2.1, 1.5 Hz, 2H), 4.26 – 4.08 (m, 2H), 3.54 (td, *J* = 4.8, 3.2 Hz, 3H), 3.30 (d, *J* = 3.6 Hz, 3H), 2.76 – 2.42 (m, 5H), 2.29 (d, *J* = 12.2 Hz, 3H), 1.79 (s, 1H). LC-MS (ES$^+$): m/z 488.09 [M+H]$^+$.

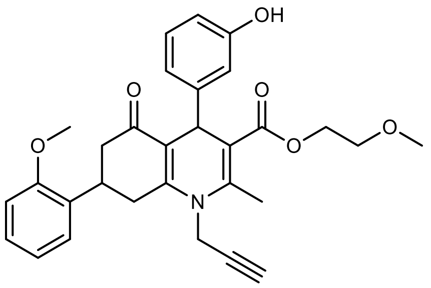

HPI-1-C

HPI-1-C. A solution of **1** (460 mg, 2 mmol), **2** (226 mg, 2 mmol), **3** (320 mg, 2 mmol) and propargyl-amine hydrochloride (184 mg, 2 mmol) in pyridine (2 mL) was refluxed for 24 hours. Then the reaction mixture was poured in cold water and extracted 3 times with EtOAc, dried with $MgSO_4$, filtered, and concentrated under reduced pressure. Finally, it was purified by column chromato-graphy (Pentane/EtOAc 6:4, R_f= 0.48) to give **HPI-1-C** as a yellow powder (230 mg, 15%). ^{1}H NMR (400 MHz, CDCl3): δ 7.26-6.59 (m, 8H), 5.17 (s, 1H), 4.35-4.18 (m, 4H), 3.80 (s, 3H), 3.60-3.65 (m, 1H), 3.55 (m, 2H), 3.32 (s, 3H), 3.06-3.10 (m, 1H 2.89-2.65 (m, 3H), 2.64 (s, 3H), 2.42 (s,1H) ppm. LC-MS (ES$^+$): m/z 502.02 [M+H]$^+$.

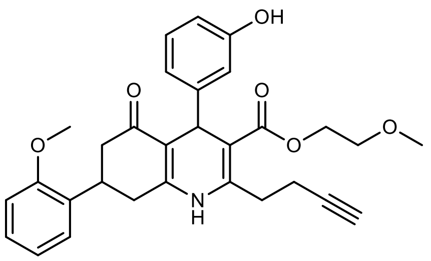

HPI-1-D

HPI-1-D was synthesised following the general procedure for Hantzsch reaction, engaging **1** (65 mg, 0.3 mmol), **2** (37 mg, 0.3 mmol), **6** (60 mg, 0.22 mmol), ammonium acetate (23 mg, 0.3 mmol), and

the ionic liquid $BMIMBF_4$ (7 µL). The resulting crude was purified by column chromatography (Pentane/EtOAc 4:7, R_f =0.41) to yield **HPI-1-D** as a yellow oil (62 mg, 41%). ^{1}H NMR (400 MHz, $CDCl_3$) δ 7.21 (d, *J* = 0.9 Hz, 1H), 7.08 (d, *J* = 16.4 Hz, 1H), 6.99 (dd, *J* = 2.6, 1.5 Hz, 1H), 6.96 – 6.87 (m, 2H), 6.84 (ddd, *J* = 8.0, 6.9, 1.1 Hz, 2H), 6.61 (ddd, *J* = 8.1, 2.6, 1.0 Hz, 1H), 5.12 (d, *J* = 18.1 Hz, 1H), 4.25 – 4.06 (m, 2H), 3.77 (d, *J* = 5.7 Hz, 3H), 3.65 – 3.56 (m, 1H), 3.53 (t, *J* = 4.8 Hz, 2H), 3.30 (d, *J* = 3.9 Hz, 3H), 3.14 – 3.02 (m, 1H), 2.80 – 2.46 (m, 7H), 2.04 (q, *J* = 2.9 Hz, 1H). LC-MS (ES$^+$): m/z 502.299 $[M+H]^+$.

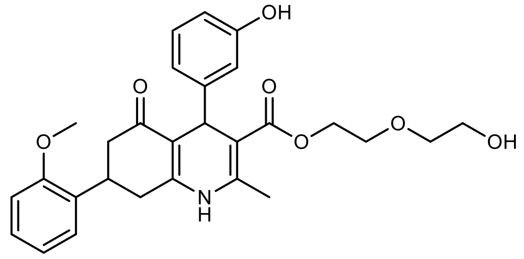

HPI-1-E

HPI-1-E was synthesised following the general procedure for Hantzsch reaction, engaging **1** (65 mg, 0.3 mmol), **2** (37 mg, 0.3 mmol), **7** (57 mg, 0.22 mmol), ammonium acetate (23 mg, 0.3 mmol), and the ionic liquid $BMIMBF_4$ (7 µL). The resulting crude was purified by column chromatography (CH_2Cl_2/ MeOH 100:6, R_f =0.52) and then reverse phase chromatography (BGB Scorpius C18 4.5 g, H_2O + 0.1% TFA/CH_3CN + 0.1% TFA 70:30 to 20:80) to yield **HPI-1-D** as a pale yellow powder (32 mg, 22%). ^{1}H NMR (500 MHz, MeOD) δ 7.25 – 7.12 (m, 2H), 7.05 – 6.90 (m, 3H), 6.79 (d, *J* = 1.8 Hz, 2H), 6.59 – 6.50 (m, 1H), 5.01 (d, *J* = 24.1 Hz, 1H), 4.24 – 4.10 (m, 2H), 3.80 (d, *J* = 8.0 Hz, 3H), 3.66 – 3.62 (m, 3H) , 3.60 – 3.40 (m, 3H), 2.86 – 2.39 (m, 4H), 2.36 (d, *J* = 8.1 Hz, 3H). LC-MS (ES$^+$): m/z 494.03 $[M+H]^+$.

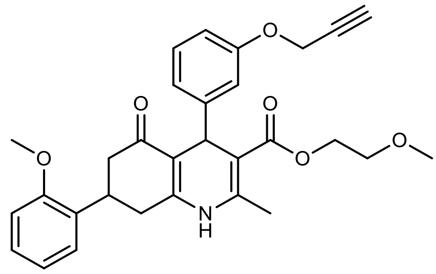

HPI-1-F

HPI-1-F. To a solution of **HPI-1** (424 mg, 0.91 mmol) in acetone (74 mL) was added anhydrous K_2CO_3 (177 mg, 1.28 mmol) followed by propargyl bromide (80% w/v in toluene, 0.16 mL, 1.83 mmol). The reaction mixture was refluxed overnight, after which it was cooled and concentrated *in vacuo*. The resulting residue was re-dissolved in EtOAc, washed with H_2O, dried over $MgSO_4$, and concentrated. The resulting crude was purified by silica gel column chromatography (pentane/EtOAc 3:2, R_f = 0.32) and reverse phase chromatography (BGB Scorpius C18 4.5 g, H_2O + 0.1% TFA/CH_3CN + 0.1% TFA 70:3 0 to 20:80) to give **HPI-1-F** as a pale-yellow solid (270 mg, 59%).^{1}H NMR (400 MHz, $CDCl_3$): δ 7.25 –

6.71 (m, 8H), 6.07 (br, 1H, NH), 5.16 (s, 1H), 4.66 – 4.58 (m, 2H), 4.20 – 4.14 (m, 2H), 3.78 (s, 3H), 3.66 – 3.58 (m, 1H), 3.52 (t, J = 5.1 Hz, 2H), 3.31 (s, 3H), 2.77 – 2.54 (m, 4H), 2.53 (t, J = 2.4 Hz, 1H), 2.38 (s, 3H). ^{13}C NMR (101 MHz, CDCl$_3$): δ 195.6, 167.2, 157.6, 157.1, 149.5, 148.7, 143.9, 130.4, 128.8, 128.0, 127.0, 121.7, 120.7, 114.9, 112.8, 112.0, 110.7, 105.7, 78.8, 75.3, 70.5, 62.9, 58.9, 55.7, 55.1, 42.4, 36 .4, 33.2, 33.0, 19.5. LC-MS (ES$^+$): m/z 502.08 [M+H]$^+$.

VHL ligand was prepared following previous reported procedures[3,4]

Pomalidomide was prepared following previous reported procedures[5]

Hydroxythalidomide and **compound 10** were prepared following previous reported procedures[6].

Linker-1 was prepared following previous reported procedures[7].

Linker-2 was prepared following previous reported procedures [8].

Linker-4 was prepared following previous reported procedures[9].

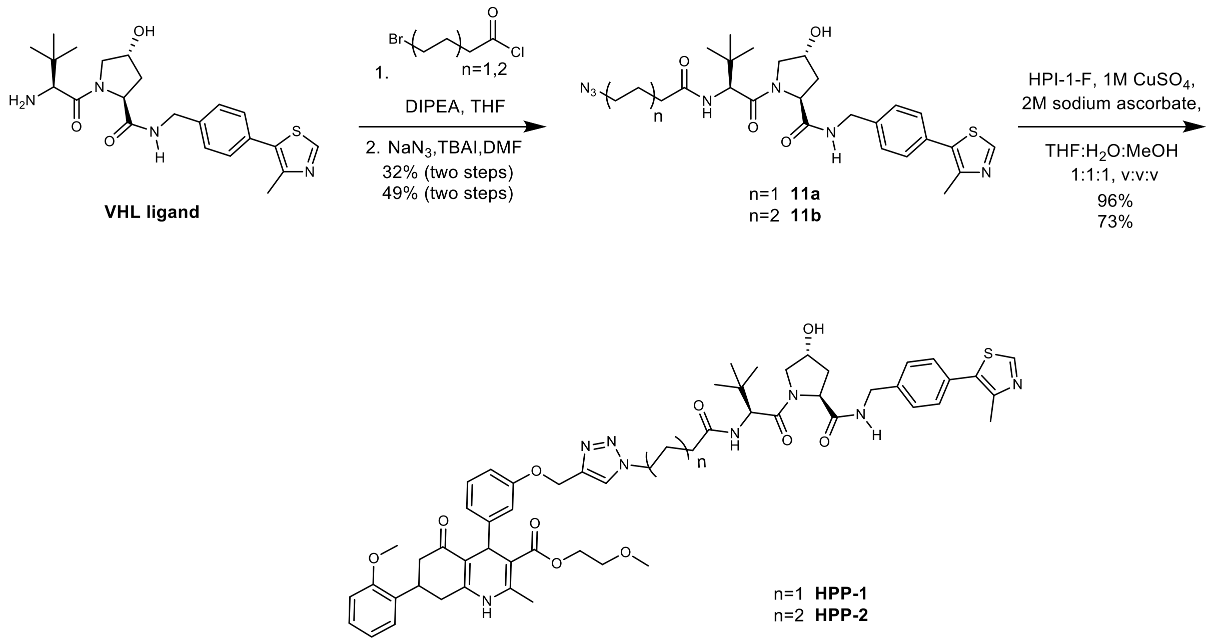

Supplementary Fig. 21. Synthesis of **HPP-1** and **HPP-2**.

Compound 11a. To a solution of **VHL ligand** (10 mg, 0.23 mmol) in anhydrous THF (1.0 mL) was added DIPEA (80 µL, 0.46 mmol) and stirred for 10 min at 0 °C. 4-bromobutanoyl chloride (54 µL, 0.46 mmol) was added slowly and stirred for 2 hours at 0 °C. Upon completion, CH_2Cl_2/MeOH (9:1) solution was added to the reaction mixture. The organic layer was washed with H_2O, dried over $MgSO_4$, and concentrated to give the crude as a brown solid; R_f= 0.52 (CH_2Cl_2/MeOH 10:1). LC-MS (ES^+): m/z 578.81 $[M+H]^+$. The resulting crude was re-dissolved in anhydrous DMF (0.3 mL). NaN_3 (34 mg, 0.52 mmol) and TBAI (3 mg, 5 mol %) were added and the mixture was stirred at 80 ℃ for 16 h. After cooling to rt, the reaction mixture was extracted with CH_2Cl_2 (×3). The organic layer was dried over $MgSO_4$ and concentrated. The resulting crude was purified by PTLC (SiO_2, CH_2Cl_2/MeOH 15:1, eluted once) to give **11a** as an off-white solid (40 mg, 32% two steps).; R_f= 0.32 (CH_2Cl_2/MeOH 15:1). ^{1}H NMR (400 MHz, $CDCl_3$): δ 8.71 (s, 1H), 7.39 – 7.33 (m, 4H), 6.11 (d, *J* = 9.0 Hz, 1H), 4.73 (t, *J* = 7.6 Hz, 1H), 4.61 – 4.54 (m, 2H), 4.47 (s, 1H), 4.33 (d, *J* = 15.7 Hz, 1H), 4.08 (d, *J* = 10.9 Hz, 1H), 3.60 (d, *J* = 11.3 Hz, 1H), 3.33 (t, *J* = 6.5 Hz, 2H), 2.62 – 2.56 (m, 1H), 2.53 (s, 3H), 2.31 (t, *J* = 6.8 Hz, 2H), 2.15 – 2.09 (m, 1H), 1.89 (p, *J* = 6.9 Hz, 2H), 0.93 (s, 9H). LC-MS (ES^+): m/z 541.85 $[M+H]^+$.

Compound 11b. VHL ligand (0.16 g, 0.37 mmol) was reacted with 6-bromohexanoyl chloride (113 µL, 0.74 mmol) according to the procedure described above. The crude product was purified by PTLC (SiO_2, CH_2Cl_2/MeOH 15:1, eluted once) to give **11b** as a white solid (104 mg, 49% two steps).; R_f= 0.25 (CH_2Cl_2/MeOH 15:1). ^{1}H NMR (400 MHz, $CDCl_3$): δ 8.71 (s, 1H), 7.39 – 7.33 (m, 4H), 6.03 (s, 1H), 4.73 (t, *J* = 8.0 Hz, 1H), 4.61 – 4.53 (m, 2H), 4.48 (s, 1H), 4.33 (d, *J* = 14.9 Hz, 1H), 4.12 (d, *J* = 11.3, 1H), 3.60 (d, *J* = 10.9 Hz, 1H, OH), 3.26 (t, *J* = 6.9 Hz, 2H), 2.62 – 2.56 (m, 1H), 2.53 (s, 3H), 2.21 (t, *J* = 7.3 Hz, 2H), 2.15 – 2.10 (m, 1H), 1.64 – 1.57 (m, 4H), 1.39 – 1.37 (m, 2H), 0.93 (s, 3H).LC-MS (ES^+): m/z 569.86 $[M+H]^+$.

HPP-1. To a solution of **11a** (26 mg, 0.05 mmol) and **HPI-1-F** (24 mg, 0.05 mmol) in degassed THF/H_2O/MeOH (2.4 mL, 1:1:2) were added degassed aqueous solutions of 2 M sodium ascorbate (48 µL, 0.10 mmol) and 1 M $CuSO_4$ (10 µL, 0.01 mmol, 20 mol%). The mixture was stirred at rt overnight, after which it was concentrated under reduced pressure. CH_2Cl_2/MeOH (9:1) and H_2O were added to the reaction mixture and after partitioning of the layers, the aqueous layer was extracted with CH_2Cl_2/MeOH (9:1). The organic layer was dried over $MgSO_4$ and concentrated. The resulting crude was purified by PTLC (SiO_2, CH_2Cl_2/MeOH 12:1, eluted once) to give **HPP-1** as a white solid (48 mg, 96%). 7.97 mg of **HPP-1** were further purified by reverse phase HPLC. Semi-preparative HPLC runs were carried out with a gradient from 5% to 85% acetonitrile/water system (0.1% TFA) for 55 min and a flow of 3 mL/min, monitored by a PDA detector at 254 nm and 360 nm. The fraction was collected and lyophilized to afford 7 mg of the product; R_f= 0.28 (CH_2Cl_2/MeOH 12:1). ^{1}H NMR (500 MHz, CD_3OD) δ 8.85 (s, 1H), 8.06 (s, 1H), 7.49 – 7.35 (m, 4H), 7.27 – 6.65 (m, 8H), 5.19 – 4.99 (m, 3H), 4.61 – 4.46

(m, 4H), 4.43 (m, 2H), 4.34 (dd, *J* = 15.4, 4.6 Hz, 1H), 4.14 (m, 2H), 3.91 (dd, *J* = 11.2, 1.8 Hz, 1H), 3.84 – 3.75 (m, 1H), 3.80 (s, 3H), 3.60 – 3.47 (m, 3H), 3.28 (s, 3H), 2.85 – 2.54 (m, 3H), 2.48 – 2.39 (m, 1H), 2.46 (s, 3H), 2.35 (s, 3H), 2.29 (m, 2H), 2.25 – 2.16 (m, 3H), 2.12 – 2.03 (m, 1H), 1.03 (s, 9H). ^{13}C NMR (126 MHz, CD_3OD) δ 197.2, 173.1, 172.9, 170.9, 167.8, 158.1, 157.1, 152.7, 151.4, 149.1, 145.3, 143.8, 138.8, 130.4, 132.0, 130.1, 128.9 , 128.7, 127.6, 126.7, 124.1, 120.8, 120.6, 120.4, 120.2, 114.4, 112.0, 111.3, 110.4, 110.2, 104.7, 70.2, 69.7, 62.6, 60.9, 59.4, 57.9, 57.7, 56.5, 54.4, 49.2, 42.3, 37.5, 36.6, 36.3, 35.0, 33.1, 32.0, 31.5, 25.6, 17.3, 14.4. LC-MS (ES$^+$): m/z 522.44 $[M+2H]^{2+}$ and m/z 1043.14 $[M+H]^+$. ESI-HRMS (m/z): calcd. for $[C_{56}H_{66}N_8O_{10}S + H]^+$ 1043.4695; obsd. 1043.4696.

HPP-2. To a solution of **11b** (30 mg, 0.05 mmol) and **HPI-1-F** (26 mg, 0.05 mmol) in degassed THF/H_2O/MeOH (2.6 ml, 1:1:2) were added degassed aqueous solutions of 2 M sodium ascorbate (53 µL, 0.11 mmol) and 1 M $CuSO_4$ (11 µL, 0.01 mmol, 20 mol%) and the resulting mixture was stirred overnight at rt, after which it was concentrated under reduced pressure. CH_2Cl_2/MeOH (9:1) and H_2O were added to the reaction mixture and after partitioning of the layers, the aqueous layer was extracted with CH_2Cl_2/MeOH (9:1). The organic layer was dried with $MgSO_4$ and concentrated. The resulting crude was purified by PTLC (SiO_2, CH_2Cl_2/MeOH 12:1, eluted once) to give **HPP-2** as an off-white solid (41 mg, 73%). 7.44 mg of **HPP-2** were further purified by reverse phase HPLC. Semi-preparative HPLC runs were carried out with a gradient from 5% to 85% acetonitrile/water system (0.1% TFA) for 55 min and a flow of 3 mL/min, monitored by a PDA detector at 254 nm and 360 nm. The fraction was collected and lyophilized to afford 6.6 mg of the product; R_f= 0.37 (CH_2Cl_2/MeOH 10:1). ^{1}H NMR (500 MHz, CD_3OD): δ 8.76 (s, 1H), 7.93 (s, 1H), 7.38 – 7.28 (m, 4H), 7.14 – 6.56 (m, 8H), 5.07 – 4.89 (m, 3H), 4.51 (s, 1H), 4.49 – 4.35 (m, 3H), 4.29 (t, *J* = 7.0 Hz, 2H), 4.24 (dd, *J* = 15.6, 2.4 Hz, 1H), 4.09 – 3.99 (m, 2H), 3.79 (d, *J* = 11.4 Hz, 1H), 3.74 – 3.64 (m, 1H), 3.70 (s, 3H), 3.49 – 3.37 (m, 3H), 3.18 (s, 3H), 2.75 – 2.46 (m, 3H), 2.38 – 2.29 (m, 4H), 2.25 (s, 3H), 2.21 – 2.08 (m, 3H), 2.01 – 1.93 (m, 1H), 1.81 (p, *J* = 7.3 Hz, 2H), 1.53 (p, *J* = 7.4 Hz, 2H), 1.20 (m, 2H), 0.92 (s, 9H). ^{13}C NMR (126 MHz, CD_3OD) δ 197.2, 174.2, 173.1, 170.9, 167.8, 158.0, 157.1, 152.7, 152.1, 151., 149., 148.6, 147.6, 145.3, 143.7, 138.8, 132.0, 130.4, 130.1, 128.9 (2C), 128.7, 127.7, 127.6 (2C), 126.7, 123.8, 120.8, 120.6, 120.4, 120.2, 114.4, 112.1, 111.3, 110.4, 110.2, 104.8, 70.2, 69.7, 62.6, 60.9, 59.4, 57.6, 56.6, 54.4, 49.8, 42.3, 37.5, 36.3, 34.8, 33.1, 32.0, 29.5, 25.6 (3C), 17.3, 14.4. LC-MS (ES$^+$): m/z 536.45 $[M+2H]^{2+}$ and m/z 1071.11 $[M+H]^+$. ESI-HRMS (m/z): calcd. for $[C_{58}H_{70}N_8O_{10}S + H]^+$ 1071.5008; obsd. 1071.5014.

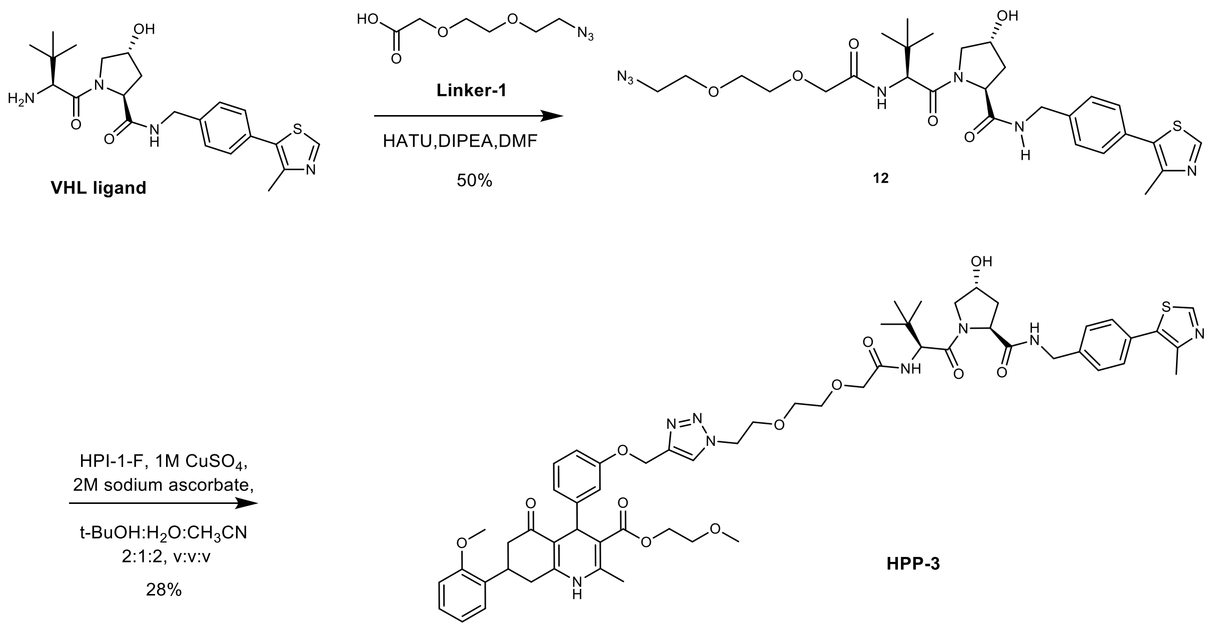

Supplementary Fig. 22. Synthesis of **HPP-3.**

Compound 12. To a solution of **VHL ligand** (68 mg, 0.16 mmol) in anhydrous DMF (3.0 mL) were added DIPEA (40 µL, 0.22 mmol). To another round-bottom flask was added **Linker-1** (33 mg, 0.17 mmol), HBTU (10 mg, 0.26 mmol) followed by DIPEA (40 µL, 0.17 mmol) in anhydrous DMF (2.0 mL). Both mixtures were stirred for 10 min separately, then combined, and stirred for another 30 min at rt. Upon completion, the reaction mixture was diluted with CH_2Cl_2, washed with sat. NH_4Cl aq. (×2). The first aqueous layer was re-extracted with CH_2Cl_2. The organic layer was dried over $MgSO_4$, concentrated. The resulting crude was purified by PTLC (SiO_2, CH_2Cl_2/MeOH 15:1, eluted once) to give **12** as a yellow solid (51 mg, 50%).; R_f= 0.35 (CH_2Cl_2/MeOH 10:1). ^{1}H NMR (400 MHz, $CDCl_3$): δ 8.76 (s, 1H), 7.83 – 7.22 (m, 4H), 4.65 (t, *J* = 7.6 Hz, 1H), 4.49 – 4.43 (m, 3H), 4.26 (d, *J* = 15.2 Hz, 1H), 4.01 (d, *J* = 11.7 Hz, 1H), 3.62 – 3.53 (m, 7H), 3.32 – 3.28 (m, 2H), 2.44 – 2.39 (m, 1H), 2.43 (s, 3H), 2.09 – 2.04 (m, 1H), 1.40 (t, *J* = 7.5 Hz, 2H), 0.88 (s, 9H). LC-MS (ES$^+$): m/z 601.84 [M+H]$^+$.

HPP-3. To a solution of **12** (21 mg, 0.035 mmol) and **HPI-1-F** (18 mg, 0.035 mmol) in degassed *t*-BuOH/CH_3CN/H_2O (1.8 mL, 2:2:1) were added degassed aqueous solutions of 2 M sodium ascorbate (87 µL, 0.2 mmol) and 1 M $CuSO_4$ (7 µL, 0.01 mmol, 20 mol%) and the mixture was stirred overnight at rt. CH_2Cl_2/MeOH (9:1) and H_2O were added to the reaction mixture and after partitioning of the layers, the aqueous layer was extracted with CH_2Cl_2/MeOH (9:1, ×2). The organic layer was dried over $MgSO_4$ and concentrated. The resulting crude was purified by reverse phase chromatography (BGB Scorpius C18 4.5 g, H_2O + 0.1% TFA/CH_3CN + 0.1% TFA 80:20 to 20:80) and silica gel column chromatography (CH_2Cl_2/MeOH 15:1) to give **HPP-3** as a yellow solid (11 mg, 28%).; R_f= 0.40 (CH_2Cl_2/MeOH 10:1). ^{1}H NMR (500 MHz, CD_3OD): δ 8.8 (s, 1H), 8.11 (s, 1H), 7.44 – 7.31 (m, 4H), 7.25 –

6.65 (m, 8H), 5.16 – 5.00 (m, 3H), 4.70 (m, 1H), 4.65 – 4.43 (m, 5H), 4.34 – 4.27 (m, 1H), 4.13 (m, 2H), 4.03 – 3.74 (m, 6H), 4.78 (s, 3H), 3.66 – 3.46 (m, 7H), 3.28 (s, 3H), 2.86 – 2.52 (m, 4H), 2.44 (s, 3H), 2.35 (s, 3H), 2.25 – 2.15 (m, 1H), 2.07 (m, 1H), 1.02 (s, 9H). ^{13}C NMR (126 MHz, CD_3OD): δ 197.1, 172.9, 170.7, 170.2, 167.8, 158.0, 157.1, 152.7, 152.1, 151.5, 149.1, 148.6, 147.6, 145.2, 138.7, 130.4, 130.1, 128.9 (2C), 128.1, 127.6 (2C), 126.6, 125.0, 120.6, 120.4, 114.5, 112.0, 111.9, 110.4, 104.7, 78.1, 70.9, 70.2, 69.9, 69.6, 69.2, 62.6, 60.8, 59.4, 57.7, 56.7, 54.4, 50.0, 42.3, 41.8, 39.9, 37.5, 36.6, 36.3, 35.8, 33.1, 32.0, 25.6(3C), 17.4, 14.5. LC-MS (ES$^+$): m/z 552.33 $[M+2H]^{2+}$ and m/z 110.13 $[M+H]^+$. ESI-HRMS (m/z): calcd. for $[C_{58}H_{70}N_8O_{12}S + H]^+$ 1103.4907; obsd. 1103.4912.

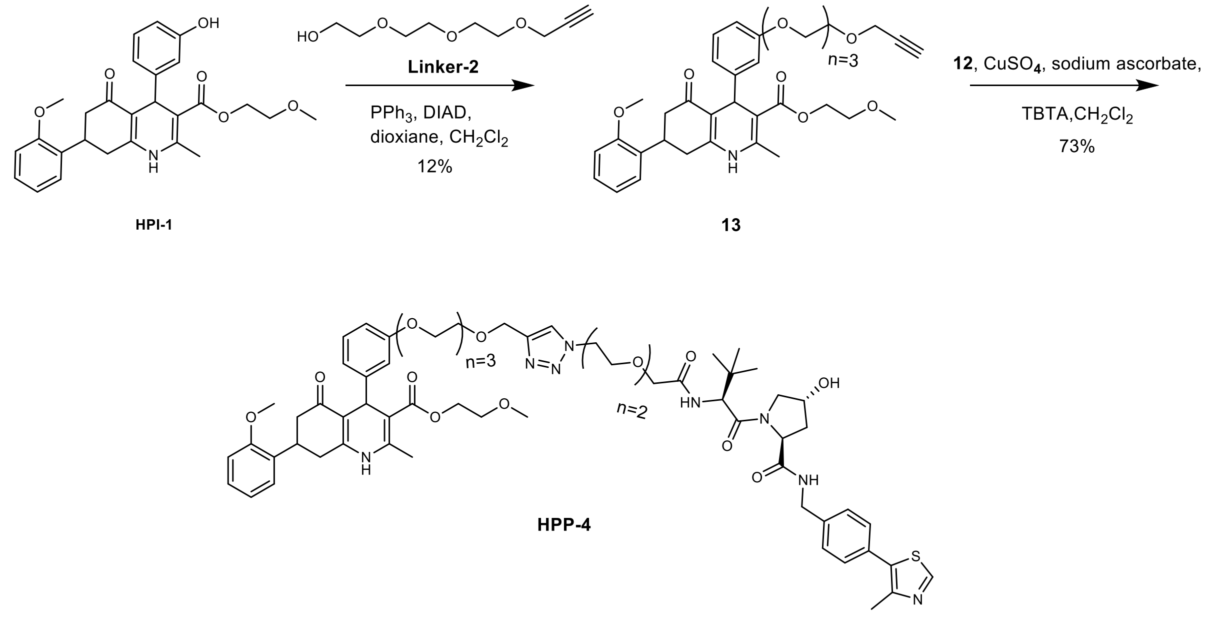

Supplementary Fig. 23. Synthesis of **HPP-4**

Compound 13. To a solution of **HPI-1** (183 mg, 0.39 mmol) in anhydrous CH_2Cl_2/dioxane (4:1, 1.0 mL) were added PPh_3 (155 mg, 0.59 mmol) and **Linker-2** (74 mg, 0.39 mmol), and the solution was ice cooled. DIAD (0.12 mL, 0.59 mmol) was added at 0 °C slowly over 15 min. The mixture was stirred at 0 °C for 30 min and then at rt overnight. The mixture was concentrated *in vacuo*. The resulting crude was purified by silica gel column chromatography (CH_2Cl_2/MeOH 30:1) and reverse phase chromatography (BGB Scorpius C18 4.5 g, H_2O + 0.1% TFA/CH_3CN + 0.1% TFA gradient from 75:35 to 35:75) to give **13** as a pale-yellow solid (30 mg, 12%).; R_f= 0.22 (CH_2Cl_2/MeOH 30:1). ^{1}H NMR (400 MHz, $CDCl_3$): δ 7.25 – 6.68 (m, 8H), 6.15 (br, 1H, NH), 5.14 (s, 1H), 4.19 (s, 2H), 4.17 (t, *J* = 4.8 Hz, 2H), 4.12 (t, *J* = 4.3 Hz, 2H), 3.83 (t, *J* = 4.7 Hz, 2H), 3.80 (s, 3H), 3.74 – 3.67 (m, 8H), 3.62 – 3.57 (m, 2H), 3.52 (t, *J* = 4.8 Hz, 2H), 3.31 (s, 3H), 2.76 – 2.56 (m, 4H), 2.41 (t, *J* = 2.3 Hz, 1H), 2.39 (s, 3H). ^{13}C NMR (101 MHz,

$CDCl_3$): δ 196.6, 167.2, 158.6, 157.2, 150.5, 148.4, 143.7, 130.2, 128.8, 128.1, 127.1, 121.1, 120.7, 114.8, 112.8, 112.0, 110.7, 106.0, 79.5, 74.7, 70.7, 70.6, 70.4, 70.4, 69.8, 69.1, 67.1, 62.9, 58.8, 58.4, 55.1, 41.9, 36.4, 33.1, 33.1, 19.5. LC-MS (ES^+): m/z 634.22 $[M+H]^+$.

HPP-4. To a solution of **13** (20 mg, 0.032 mmol), **12** (19 mg, 0.032 mmol) and TBTA (5 mg, 9 µmol) in CH_2Cl_2 (2.0 mL) was added a solution of sodium ascorbate (6 mg, 0.032 mmol) and $CuSO_4$ (5 mg, 0.032 mmol) in H_2O (0.1 mL). The mixture was vigorously stirred overnight at rt. The mixture was diluted with CH_2Cl_2, dried over $MgSO_4$ and concentrated. The resulting crude was purified by PTLC (SiO_2, CH_2Cl_2/MeOH 10:1, eluted once) to give **HPP-4** as a white solid (41 mg, 73%). 12 mg of **HPP-4** were further purified by reverse phase HPLC. Semi-preparative HPLC runs were carried out with a gradient from 5% to 95% acetonitrile/water system (0.1% TFA) for 55 min and a flow of 3 mL/min, monitored by a PDA detector at 254 nm and 360 nm. The fraction was collected and lyophilized to afford 10 mg of the product; R_f= 0.35 (CH_2Cl_2/MeOH 10:1). ^{1}H NMR (500 MHz, CD_3OD) δ 8.06 (s, 1H), 7.44 (s, 1H), 7.42 – 7.33 (m, 4H), 7.25 – 6.62 (m, 8H), 5.06 (s, 1H), 4.71 (s, 1H), 4.61 – 4.47 (m, 7H), 4.32 (d, *J* = 15.4 Hz, 1H), 4.13 (m, 2H), 4.10 – 4.05 (m, 2H), 4.02 – 3.94 (m, 2H), 3.91 – 3.72 (m, 10H), 3.69 – 3.57 (m, 12H), 3.53 (m, 2H), 2.85 – 2.54 (m, 3H), 2.49 – 2.38 (m, 4H), 2.35 (s, 3H), 2.24 (m, 1H), 2.08 (m, 1H), 1.03 (s, 9H). ^{13}C NMR (126 MHz, CD_3OD) δ 197.2, 172.9, 170.6, 170.1, 167.8, 158.6, 157.1, 152.7, 149.0, 145.2, 138.8, 130.3, 128.9 (2C), 128.6, 127.6 (2C), 126.6, 120.4, 120.2, 114.3, 111.5, 110.4, 104.8, 70.9, 70.3, 70.2, 70.2, 70.1, 69.9, 69.7, 69.6, 69.5, 69.3, 69.2, 66.9, 63.7, 62.6, 59.4, 57.7, 56.6, 54.4, 49.9, 42.3, 37.6, 36.2, 35.9, 33.1, 32.0, 25.5 (3C), 17.3, 14.5. LC-MS (ES^+): m/z 618.54 $[M+2H]^{2+}$ and m/z 1235.16 $[M+H]^+$. ESI-HRMS (m/z): calcd. for $[C_{64}H_{82}N_8O_{10}S + H]^+$ 1235.5693; obsd. 1235.5684.

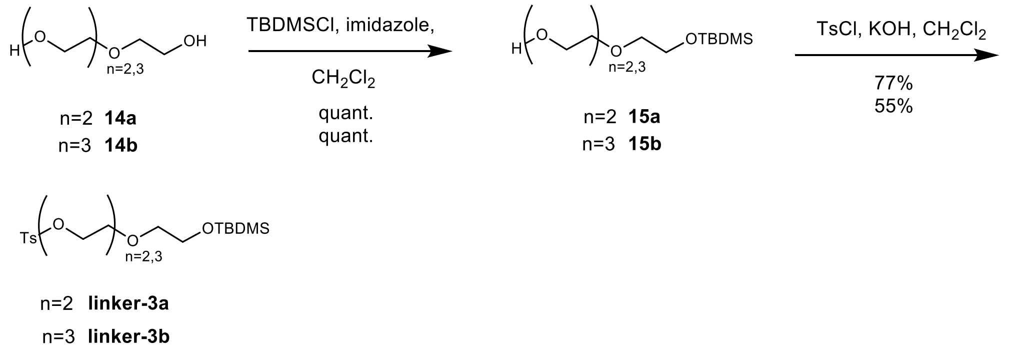

Supplementary Fig. 24. Synthesis of **linker-3a** and **3b**

Compound 15a. To a solution TBDMSCl (1.21 g, 8.0 mmol) and imidazole (907 mg, 13.3 mmol) in anhydrous DMF (15 mL) was added triethylene glycol **14a** (2.0 g, 13.3 mmol). The reaction mixture was stirred at rt for 4 h. The reaction mixture was concentrated and co-evaporated with toluene. The

resulting crude was purified by silica gel column chromatography (petroleum ether/EtOAc 1:1) to give **14a** as a colorless oil (0.60 g, 29%).; R_f= 0.40 (pentane/EtOAc 1:1). ^{1}H NMR (400 MHz, $CDCl_3$): δ 3.76 (t, *J* = 5.2 Hz, 2H), 3.73 – 3.70 (m, 2H), 3.66 (s, 4H), 3.61 – 3.59 (m, 2H), 3.56 (t, *J* = 5.2 Hz, 2H), 2.46 (br, 1H, OH), 0.89 (s, 9H), 0.06 (s, 6H). LC-MS (ES^+): m/z 264.94 $[M+H]^+$.

Compound 15b. Tetraethylene glycol **14b** (4.51 g, 23.2 mmol) and imidazole (379 mg, 5.6 mmol) were dissolved in 3 mL anhydrous CH_2Cl_2. The reaction mixture was cooled down to 0 °C and a solution of TBDMSCl (0.80 ml, 4.64 mmol) in anhydrous CH_2Cl_2 (7.0 mL) was added dropwise over 2 h. The reaction mixture was stirred at rt overnight. H_2O (10 mL) was added to the mixture and the organic layer was washed thrice with H_2O to remove the excess tetraethylene glycol. The organic layer was dried with $MgSO_4$ and concentrated. Compound **15b** was obtained as a colorless oil (1.46 g, quant.), which was used directly for the next step without further purification.; R_f= 0.27 (pentane/EtOAc 1:1). ^{1}H NMR (400 MHz, $CDCl_3$): δ 3.76 (t, *J* = 5.4 Hz, 2H), 3.73 – 3.70 (m, 2H), 3.68 – 3.64 (m, 8H), 3.63 – 3.60 (m, 2H), 3.56 (t, *J* = 5.3 Hz, 2H), 0.89 (s, 9H), 0.06 (s, 6H). LC-MS (ES^+): m/z 309.01 $[M+H]^+$.

Linker-3a. To a solution of **15a** (600 mg, 2.30 mmol) in anhydrous CH_2Cl_2 (10.0 mL) was added anhydrous KOH (510 mg, 9.10 mmol). The reaction mixture was stirred for 15 min at 0 °C before TsCl (0.52 g, 2.70 mmol) was added carefully, and stirred for 3 h. A solution of cold H_2O and CH_2Cl_2 was added into the reaction mixture. The aqueous layer was extracted with CH_2Cl_2 (×2). The organic layer was washed with cold H_2O, dried over $MgSO_4$ and concentrated. The resulting crude was purified by silica gel column chromatography (pentane/EtOAc 7:1) to give **Linker-3a** as a colorless oil (0.74 g, 77 %).; R_f= 0.29 (pentane/EtOAc 7:1). ^{1}H NMR (400 MHz, $CDCl_3$): δ 7.80 (d, *J* = 8.4 Hz, 2H), 7.34 (d, *J* = 7.9 Hz, 2H), 4.16 (t, *J* = 4.6 Hz, 2H), 3.74 (t, *J* = 5.6 Hz, 2H), 3.69 (t, *J* = 4.9 Hz, 2H), 3.59 – 3.56 (m, 4H), 3.52 (t, *J* = 5.0 Hz, 2H), 2.44 (s, 3H), 0.88 (s, 9H), 0.05 (s, 6H). LC-MS (ES^+): m/z 418.91 $[M+H]^+$.

Linker-3b. 15b (1.46 g, 4.74 mmol) was reacted according to the procedure of **15a** described above to yield **Linker-3b** as a colorless oil (1.21 g, 55 %).; R_f= 0.50 (pentane/EtOAc 2:1). ^{1}H NMR (400 MHz, $CDCl_3$): δ 7.80 (d, *J* = 8.3 Hz, 2H), 7.33 (d, *J* = 8.3 Hz, 2H), 4.16 (t, *J* = 5.0 Hz, 2H), 3.75 (t, *J* = 5.5 Hz, 2H), 3.68 (t, *J* = 5.0 Hz, 2H), 3.65 – 3.59 (m, 4H), 3.58 (s, 4H), 3.54 (t, *J* = 5.6 Hz, 2H), 2.44 (s, 3H), 0.89 (s, 9H), 0.06 (s, 6H). LC-MS (ES^+): m/z 462.86 $[M+H]^+$.

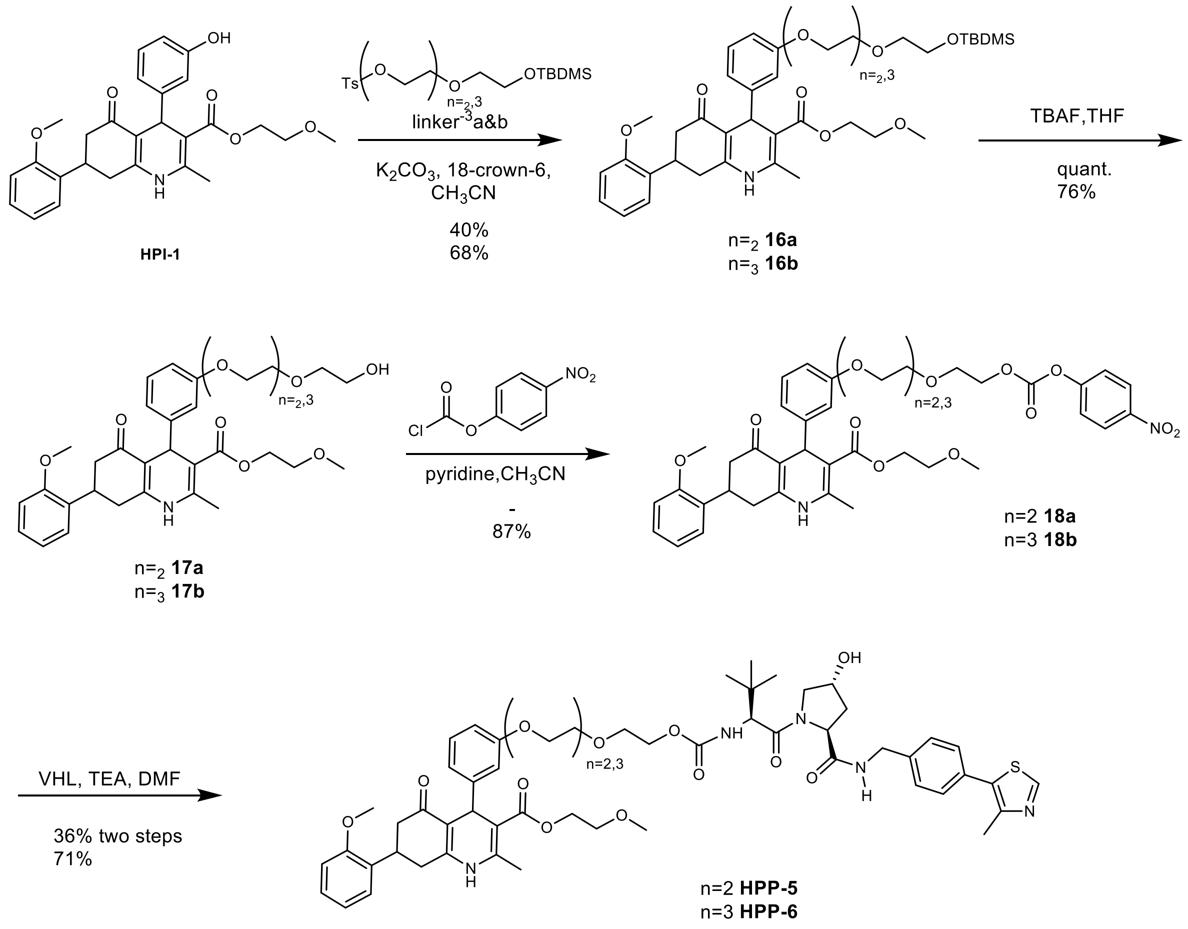

Supplementary Fig. 25. Synthesis of **HPP-5** and **HPP-6**

Compound 16a. To a solution of **HPI-1** (240 mg, 0.52 mmol) in anhydrous CH_3CN (20 mL) were added **Linker-3a** (180 mg, 0.43 mmol), K_2CO_3 (360 mg, 2.60 mmol) and 18-Crown-6 (11 mg, 40 µmol). The reaction mixture was refluxed overnight, after which it was cooled to rt and filtered. The filtrate was concentrated *in vacuo*. To remove the remaining salt, the acquired oil was re-dissolved in EtOAc, washed with H_2O (×2) and brine, dried over $MgSO_4$ and concentrated. The resulting crude was purified by silica gel column chromatography (pentane/EtOAc 1:1 to 1:2) to give **16a** as a yellow oil (115 mg, 40 %).; R_f= 0.36 (pentane/EtOAc 1:1). ^{1}H NMR (400 MHz, $CDCl_3$): δ 7.23 (d, *J* = 8.0 Hz, 1H), 7.14 (t, *J* = 8.0 Hz, 2H), 7.01 (d, *J* = 7.3 Hz, 1H), 6.94 (t, *J* = 7.0 Hz, 2H), 6.88 (d, *J* = 8.6 Hz, 1H), 6.69 (d, *J* = 8.0 Hz, 1H), 5.81 (br, 1H, NH), 5.15 (s, 1H), 4.16 (t, *J* = 5.5 Hz, 2H), 4.12 (t, *J* = 4.5 Hz, 2H), 3.84 (t, *J* = 4.5 Hz, 2H), 3.81 (s, 3H), 3.77 (t, *J* = 5.5 Hz, 2H), 3.71 – 3.68 (m, 4H), 3.65 – 3.61 (m, 1H), 3.57 (t, *J* = 5.5 Hz, 2H), 3.52 (t, *J* = 4.5 Hz, 2H), 3.31 (s, 3H), 2.76 – 2.53 (m, 4H), 2.40 (s, 3H), 0.89 (s, 9H), 0.06 (s, 6H). LC-MS (ES$^+$): m/z 710.30 [M+H]$^+$.

Compound 16b. To a solution of **HPI-1** (190 mg, 0.42 mmol) in anhydrous CH_3CN (8.0 mL) were added **Linker-3b** (240 mg, 0.52 mmol), K_2CO_3 (430 mg, 3.12 mmol), and 18-Crown-6 (14 mg, 50 µmol). The reaction mixture was refluxed overnight, after which it was cooled to room temperature and poured

into EtOAc, washed with H_2O (×2) and brine, dried over $MgSO_4$ and concentrated. The resulting crude was purified by silica gel column chromatography (CH_2Cl_2/MeOH 10:0 to 10:1) and another silica gel column chromatography (Pentane/EtOAc 1:1 to 1:2) to give **16b** as a yellow oil (0.265 g, 68%).; R_f= 0.22 (pentane/EtOAc 1:1). ^{1}H NMR (400 MHz, $CDCl_3$): δ 7.23 (d, *J* = 8.0 Hz, 1H), 7.13 (t, *J* = 8.0 Hz, 2H), 7.01 (d, *J* = 7.4 Hz, 1H), 6.93 (t, *J* = 7.5 Hz, 2H), 6.86 (d, *J* = 8.0 Hz, 1H), 6.68 (d, *J* = 7.1 Hz, 1H), 6.15 (br, 1H, NH), 5.14 (s, 1H), 4.16 (t, *J* = 5.5 Hz, 2H), 4.11 (t, *J* = 5.5 Hz, 2H), 3.82 (t, *J* = 5.1 Hz, 2H), 3.79 (s, 3H), 3.76 (t, *J* = 5.5 Hz, 2H), 3.72 – 3.67 (m, 5H), 3.65 (s, 4H), 3.55 (t, *J* = 5.3 Hz, 2H), 3.51 (t, *J* = 5.0 Hz, 2H), 3.30 (s, 3H), 2.77 – 2.53 (m, 4H), 2.38 (s, 3H), 0.88 (s, 9H), 0.06 (s, 6H). LC-MS (ES^+): m/z 754.36 $[M+H]^+$.

Compound 17a. To a solution of **16a** (120 mg, 0.16 mmol) in anhydrous THF (3.0 mL) was added 1.0 M TBAF in THF (90 µl, 0.32 mmol) and the mixture was stirred at 40 °C overnight. The reaction mixture was concentrated, and the resulting oil was re-dissolved in EtOAc. The organic layer was washed with water (×8), sat. NH_4Cl aq. (×3) and brine, dried over $MgSO_4$ and concentrated. Compound **17a** was obtained as a pale-yellow oil (102 mg, quant.), which was used directly for the next step without further purification.; R_f= 0.35 (CH_2Cl_2/MeOH 15:1). ^{1}H NMR (400 MHz, $CDCl_3$): δ 7.25 – 6.68 (m, 8H), 6.02 (br, 1H, NH), 5.15 (s, 1H), 4.18 – 4.11 (m, 4H), 3.85 (t, *J* = 5.0 Hz, 2H), 3.80 (s, 3H), 3.74 – 3.68 (m, 6H), 3.65 – 3.58 (m, 1H), 3.61 (t, *J* = 4.2 Hz, 2H), 3.52 (t, *J* = 5.0 Hz, 2H), 3.30 (s, 3H), 2.79 – 2.53 (m, 4H), 2.40 (s, 3H). LC-MS (ES^+): m/z 596.15 $[M+H]^+$.

Compound 17b. 16b (0.22 g, 0.29 mmol) was reacted according to the procedure of **16a** described above. The resulting crude was purified by silica gel column chromatography (EtOAc 100% to CH_2Cl_2/MeOH 10:1) to give **17b** as a yellow oil (140 mg, 76%).; R_f= 0.27 (CH_2Cl_2/MeOH 10:1). ^{1}H NMR (400 MHz, $CDCl_3$): δ 7.23 – 6.66 (m, 8H), 5.13 (s, 1H), 4.17 (t, *J* = 5.0 Hz, 2H), 4.13 – 4.10 (m, 2H), 5.56 – 3.79 (m, 2H), 3.80 (s, 3H), 3.72 – 3.66 (m, 10H), 3.60 – 3.58 (m, 3H), 3.52 (t, *J* = 5.0 Hz, 2H), 3.31 (s, 3H), 2.81 – 2.58 (m, 4H), 2.41 (s, 3H). LC-MS (ES^+): m/z 640.22 $[M+H]^+$.

HPP-5. A stirred mixture of 4-nitrophenyl chloroformate (51 mg, 0.25 mmol) and pyridine (30 µL, 0.34 mmol) in CH_3CN (2.0 mL) was cooled to 0 °C for 15 min. A solution of **17a** (100 mg, 0.17 mmol) in anhydrous CH_3CN (2.0 mL) was added slowly. The reaction mixture was stirred at rt overnight, after which it was concentrated *in vacuo*. The resulting crude was re-dissolved in CH_2Cl_2 and washed with brine. The organic layer was dried over $MgSO_4$ and concentrated to give crude **18a.** LC-MS (ES^+): m/z 761.18 $[M+H]^+$. The crude containing **18a** was re-dissolved in anhydrous DMF (20 mL). **VHL** (40 mg, 92.1 µmol) and Et_3N (50 µL, excess) were added, and stirred at rt overnight. The reaction mixture was concentrated and purified by PTLC (SiO_2, CH_2Cl_2/MeOH 17:1, eluted twice) to give **HPP-5** as a yellow solid (65 mg, 36% two steps). 6.14 mg of **HPP-5** were further purified by reverse phase HPLC. Semi-preparative HPLC runs were carried out with a gradient from 5% to 85% acetonitrile/water system

(0.1% TFA) for 55 min and a flow of 3 mL/min, monitored by a PDA detector at 254 nm and 360 nm. The fraction was collected and lyophilized to afford 5 mg of the product; R_f= 0.35 (CH_2Cl_2/MeOH 10:1).; R_f= 0.39 (CH_2Cl_2/MeOH 15:1). ^{1}H NMR (500 MHz, CD_3OD): δ 8.87 (s, 1H), 7.47 – 7.37 (m, 4H), 7.24 – 6.67 (m, 8H), 5.06 (s, 1H), 4.57 (t, *J* = 8.3 Hz, 1H), 4.52 (m, 2H), 4.34 (d, *J* = 13.4 Hz, 2H), 4.20 – 4.06 (m, 6H), 3.88 (d, *J* = 11.1 Hz, 1H), 3.81 (d, *J* = 7.5 Hz, 6H), 3.71 – 3.62 (m, 6H), 3.59 – 3.48 (m, 3H), 3.30 (s, 3H), 2.86 – 2.50 (m, 3H), 2.47 (s, 4H), 2.36 (s, 3H), 1.01 (s, 9H). ^{13}C NMR (126 MHz, CD_3OD): δ 197.2, 173.0, 171.2, 167.8, 158.6, 157.1, 152.6, 151.4, 148.9, 147.5, 145.2, 138.8, 132.0, 130.4, 130.0, 128.9 (2C), 128.5, 127.7, 127.6 (2C), 126.6, 120.4, 120.2, 114.3, 111.5, 111.4, 110.4, 104.8, 70.3, 70.2, 70.1, 69.6, 69.5, 69.1, 66.9, 64.0, 62.6, 59.5, 59.4, 57.7, 56.5, 54.4, 47.8, 42.3, 42.2, 37.5, 36.2, 35.2, 33.1, 32.0, 25.5 (3C), 17.3, 14.4. LC-MS (ES$^+$): m/z 527.03 $[M+2H]^{2+}$ and m/z 1052.11 $[M+H]^+$. ESI-HRMS (m/z): calcd. for $[C_{56}H_{69}N_5O_{13}S + H]^+$ 1052.4685; obsd. 1052.4683.

HPP-6. 4-Nitrophenyl chloroformate (88 mg, 0.44 mmol) was reacted with **17b** (0.140 g, 0.22 mmol) according to the procedure of **17a** described above. The resulting crude was purified by silica gel column chromatography (pentane/EtOAc 1:4 to 100 % EtOAc) to give **18b** (0.153 g, 87%); R_f = 0.36 (pentane/EtOAc 1:4). ^{1}H NMR (400 MHz, $CDCl_3$): δ 8.26 (d, *J* = 9.1 Hz, 2H). 7.37 (d, *J* = 9.1 Hz, 2H), 7.24 – 6.64 (m, 8H), 5.90 (br, 1H, NH), 5.14 (s, 1H), 4.42 (t, *J* = 4.6 Hz, 2H), 4.16 (t, *J* = 4.7 Hz, 2H), 4.13 – 4.11 (m, 2H), 3.84 (t, *J* = 5.0 Hz, 2H), 3.81 – 3.79 (m, 2H), 3.73 – 3.68 (m, 8H), 3.64 – 3.57 (m, 4H), 3.51 (t, *J* = 5.0 Hz, 2H), 3.31 (s, 3H), 2.79 – 2.52 (m, 4H), 2.39 (s, 3H). LC-MS (ES$^+$): m/z 805.24 $[M+H]^+$. **18b** (145 mg, 0.18 mmol) was re-dissolved in anhydrous DMF (5.0 mL). **VHL ligand** (109 mg, 0.25 mmol) and Et_3N (130 µL, excess) were added, and stirred overnight at room temperature. The reaction mixture was concentrated and purified by PTLC (SiO_2, CH_2Cl_2/MeOH 13:1 and 15:1, eluted once, respectively) to give **HPP-6** as a yellow solid (140 mg, 71%).; R_f= 0.28 (CH_2Cl_2/MeOH 13:1). ^{1}H NMR (500 MHz, CD_3OD): δ 8.74 (s, 1H), 7.35 – 7.26 (m, 4H), 7.12 – 6.55 (m, 8H), 4.96 (s, 1H), 4.48 (t, *J* = 8.3 Hz, 1H), 4.44 – 4.35 (m, 2H), 4.27 – 4.20 (m, 2H), 4.10 – 3.92 (m, 6H), 3.77 (d, *J* = 11.0 Hz, 1H), 3.74 – 3.66 (m, 6H), 3.58 – 3.48 (m, 10H), 3.46 – 3.39 (m, 3H), 3.19 (s, 3H), 2.71 – 2.45 (m, 3H), 2.35 (s, 4H), 2.25 (s, 3H), 2.15 – 2.06 (m, 1H), 1.97 (m, 1H), 0.91 (s, 9H). ^{13}C NMR (126 MHz, CD_3OD): δ 197.2, 173.0, 171.2, 167.8, 158.6, 157.1, 152.6, 151.4, 149.0, 147.6, 145.2, 138.8, 132.0, 130.3, 130.1, 128.9 (2C), 128.6, 127.7, 127.5 (2C), 126.8, 126.6, 120.4, 120.2, 114.3, 111.5, 110.4, 104.8, 78.1, 70.3, 70.2, 70.2, 70.1, 70.1, 69.7, 69.5, 69.0, 66.9, 64.0, 62.6, 59.5, 59.4, 57.7, 56.6, 54.4, 42.3, 37.5, 36.5, 36.2, 35.3, 33.1, 32.0, 25.6 (3C), 17.4, 14.5. LC-MS (ES$^+$): m/z 548.75 $[M+2H]^{2+}$ and m/z 1096.42 $[M+H]^+$. ESI-HRMS (m/z): calcd. for $[C_{58}H_{73}N_5O_{14}S + H]^+$ 1096.4948; obsd. 1096.4944.

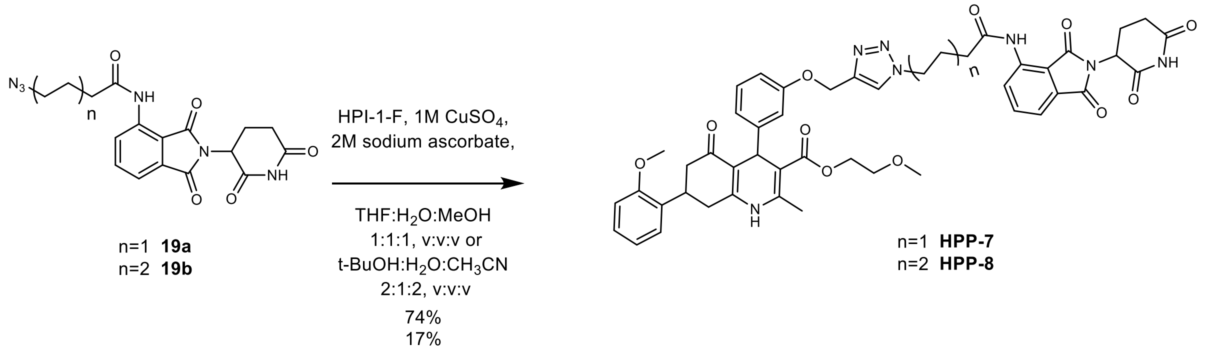

Supplementary Fig. 26. Synthesis of **HPP-7** and **HPP-8**

Compound 19a and **19b** were prepared following previous reported procedures[10]**.**

HPP-7. To a solution of **19a** (20 mg, 0.052 mmol) and **HPI-1-F** (26 mg, 0.052 mmol) in degassed THF/H_2O/MeOH (3.6 ml, 1:1:1) were added degassed aqueous solutions of 2 M sodium ascorbate (52 µL, 0.10 mmol) and 1 M $CuSO_4$ (10 µL, 0.01 mmol, 20 mol%). The mixture was stirred at rt overnight, after which it was concentrated under reduced pressure. The reaction mixture washed with H_2O and extracted with CH_2Cl_2. The aqueous layer was re-extracted with CH_2Cl_2 (×6). The combined organic layers were dried over $MgSO_4$ and concentrated. The resulting crude was purified by PTLC (SiO_2, CH_2Cl_2/MeOH 13:1, eluted once) to give **HPP-7** as an off-white solid (34 mg, 74%).; R_f= 0.28 (CH_2Cl_2/MeOH 13:1). ^{1}H NMR (500 MHz, $CDCl_3$): δ 9.39 (br, 1H, NH), 8.76 (d, *J* = 8.5 Hz, 1H), 8.19 (d, *J* = 16.4 Hz, 1H, NH), 7.74 (s, 1H), 7.72 – 7.66 (m, 1H), 7.55 (d, *J* = 7.3 Hz, 1H), 7.25 – 6.67 (m, 8H), 5.99 (br, 1H, NH), 5.26 – 5.07 (m, 3H), 4.94 (m, 1H), 4.49 (t, *J* = 6.6 Hz, 2H), 4.26 – 4.10 (m, 2H), 3.79 (s, 3H), 3.68 – 3.55 (m, 1H), 3.52 (t, *J* = 4.9 Hz, 2H), 3.29 (s, 3H), 2.95 – 2.53 (m, 7H), 2.50 (t, *J* = 7.0 Hz, 2H), 2.38 – 2.32 (m, 4H), 2.19 – 2.09 (m, 1H). ^{13}C NMR (126 MHz, $CDCl_3$): δ 193.3, 168.2, 166.6, 165.4, 165.4, 164.9, 164.3, 155.6, 154.8, 147.0, 146.4, 142.2, 141.5, 141.5, 135.1, 134.1, 128.8, 128.1, 126.6, 125.7, 124.7, 122.9, 120.9, 119.0, 118.9, 118.4, 116.4, 112.3, 110.5, 110.2, 108.3, 68.1, 60.6, 59.4, 56.5, 52.8, 46.9, 46.8, 40.0, 34.2, 34.2, 31.4, 30.8, 29.0, 23.0, 20.3, 17.2. LC-MS (ES^+): m/z 886.15 $[M+H]^+$. ESI-HRMS (m/z): calcd. for $[C_{47}H_{47}N_7O_{11} + H]^+$ 886.3406; obsd. 886.3409.

HPP-8. To a solution of compound **19b** (13 mg, 0.032 mmol) and **HPI-1-F** (16 mg, 0.032 mmol) in degassed *t*-BuOH/CH_3CN/H_2O (1.6 ml, 2:2:1) were added degassed aqueous solutions of 2 M sodium ascorbate (160 µL, 0.32 mmol) and 1 M $CuSO_4$ (16 µL, 0.02 mmol, 50 mol%) and the mixture was stirred at rt overnight. CH_2Cl_2 and H_2O were added to the reaction mixture and after partitioning of the layers, the aqueous layer was extracted with CH_2Cl_2. The organic layer was dried over $MgSO_4$ and concentrated. The resulting crude was purified by silica gel column chromatography (CH_2Cl_2/MeOH 50:1 to 20:1) to give **HPP-8** as a white solid (5 mg, 17%).; R_f= 0.23 (CH_2Cl_2/MeOH 20:1). ^{1}H NMR (500 MHz, $CDCl_3$): δ 9.40 (br, 1H, NH), 8.79 (d, *J* = 8.5 Hz, 1H), 8.36 (d, *J* = 11.6 Hz, 1H, NH), 7.77 – 7.61 (m,

2H), 7.53 (d, *J* = 7.2 Hz, 1H), 7.24 – 6.69 (m, 8H), 6.12 (br, 1H, NH), 5.25 – 5.06 (m, 3H), 4.99 – 4.87 (m, 1H), 4.36 (t, *J* = 6.9 Hz, 2H), 4.16 (t, *J* = 4.6, 2H), 3.79 (s, 3H), 3.65 – 3.54 (m, 1H), 3.54 – 3.49 (m, 2H), 3.29 (s, 3H), 2.91 – 2.51 (m, 7H), 2.45 (dd, *J* = 8.3, 6.6 Hz, 2H), 2.35 (s, 3H), 2.21 – 2.11 (m, 1H), 2.00 – 1.90 (m, 2H), 1.78 (m, 2H), 1.43 - 1.38 (m, 2H). ^{13}C NMR (126 MHz, $CDCl_3$): δ 195.6, 171.8, 170.7, 169.1, 167.3, 166.7, 158.0, 157.1, 149.5, 148.8, 144.0, 137.7, 136.5, 131.1, 130.4, 128.9, 128.0, 127.1, 127.0, 125.2, 121.4, 121.3, 120.7, 120.6, 118.5, 115.4, 114.7, 112.4(2C), 110.7, 105.6, 70.5, 62.9, 61.8, 58.8, 55.2, 50.1, 49.3, 42.4, 37.4, 36.6, 33.2, 33.1, 31.3, 29.9, 25.9, 24.4, 22.7, 19.5. LC-MS (ES$^+$): m/z 457.82 $[M+2H]^{2+}$ and m/z 914.16 $[M+H]^+$. ESI-HRMS (m/z): calcd. for $[C_{49}H_{51}N_7O_{11} + H]^+$ 914.3719; obsd. 914.3719.

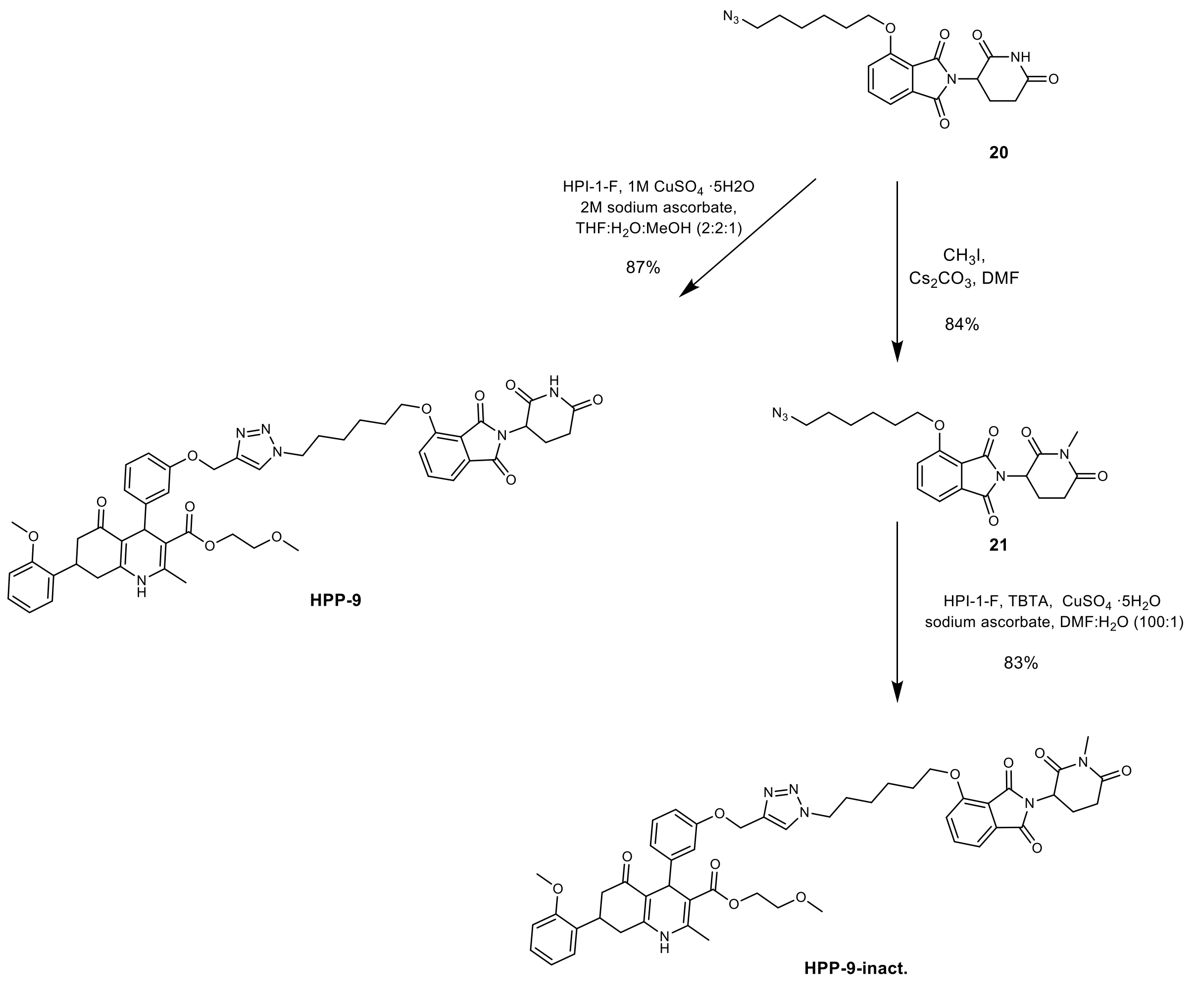

Supplementary Fig. 27. Synthesis of **HPP-9** and **HPP-9-inact.**

Compound 20 was prepared following previous reported procedures[6].

HPP-9. To a solution of **20** (23 mg, 0.058 mmol) and **HPI-1-F** (23 mg, 0.046 mmol) in degassed THF/H_2O/MeOH (3.0 ml, 1:1:1) were added degassed aqueous solutions of 2 M sodium ascorbate (46

µL, 0.092 mmol) and 1 M $CuSO_4$ (9 µL, 0.009 mmol, 20 mol%). The mixture was stirred overnight at rt, after which it was concentrated under reduced pressure. CH_2Cl_2 and H_2O were added to the reaction mixture and after partitioning of the layers, the aqueous layer was extracted with CH_2Cl_2 (×6). The organic layer was dried over $MgSO_4$ and concentrated. The resulting crude was purified by PTLC (SiO_2, CH_2Cl_2/MeOH 13:1, eluted once) to give **HPP-9** as a white solid (36 mg, 87%).; R_f= 0.49 (CH_2Cl_2/MeOH 10:1). ^{1}H NMR (500 MHz, $CDCl_3$): δ 8.28 (br, 1H, NH), 7.72 (d, *J* = 7.9 Hz, 1H), 7.66 (dd, *J* = 8.2, 7.2 Hz, 1H), 7.44 (dd, *J* = 7.4, 1.9 Hz, 1H), 7.26 – 6.69 (m, 9H), 6.12 (br, 1H, NH), 5.24 – 5.07 (m, 3H), 4.93 (dd, *J* = 12.4, 5.4 Hz, 1H), 4.42 – 4.31 (m, 2H), 4.23 – 4.09 (m, 4H), 3.79 (s, 3H), 3.66 – 3.52 (m, 1H), 3.52 (t, *J* = 5.0 Hz, 2H), 3.29 (s, 3H), 2.90 – 2.51 (m, 7H), 2.36 (s, 3H), 2.15 – 2.07 (m, 1H), 2.01 – 1.90 (m, 2H), 1.85 (m, 2H), 1.57 (p, *J* = 7.5 Hz, 2H), 1.47 – 1.37 (m, 2H). ^{13}C NMR (126 MHz, $CDCl_3$): δ 195.6, 170.9, 168.2, 167.3, 167.1, 165.8, 158.0, 157.2, 156.9, 156.6, 149.5, 148.8, 144.2, 144.0, 136.6, 133.8, 130.5, 128.9, 128.0, 127.1, 123.1, 121.4, 120.8, 118.9, 117.2, 115.9, 114.7, 112.8, 112.5, 110.7, 105.6, 70.5, 69.1, 62.9, 61.8, 58.9, 55.2, 50.2, 49.1, 42.5, 36.6, 33.2, 31.4, 29.9, 28.4, 25.9, 25.3, 22.6, 19.5. LC-MS (ES^+): m/z 451.35 $[M+2H]^{2+}$ and m/z 901.14 $[M+H]^+$. ESI-HRMS (m/z): calcd. for $[C_{50}H_{54}N_6O_{11} + H]^+$ 901.3767; obsd. 901.3771.

Compound 21. To a solution of **20** (30 mg, 0.072 mmol) in DMF (1 mL) was added Cs_2CO_3 (40 mg, 0.13 mmol) and CH_3I (0.01 mL, 0.11 mmol) at rt. The reaction mixture was stirred for 2h at the same temperature and additional CH_3I was added and let it stir for another 2 hours. Then the mixture was diluted with EtOAc and quenched with HCl 1N. Then washed 3 times with H_2O and one with brine, dried with $MgSO_4$ and concentrated in vacuo. Purification by PTLC with 30:1 CH_2Cl_2:MeOH (25 mg, 84%). (R_F: 0.78 in CH_2Cl_2:MeOH 30:1). ^{1}H NMR (400 MHz, $CDCl_3$) δ 7.66 (dd, *J* = 8.5, 7.3 Hz, 1H), 7.44 (dd, *J* = 7.3, 0.7 Hz, 1H), 7.20 (dd, *J* = 8.5, 0.7 Hz, 1H), 5.00 – 4.91 (m, 1H), 4.18 (t, *J* = 6.4 Hz, 2H), 3.29 (t, *J* = 6.9 Hz, 2H), 3.20 (s, 3H), 3.02 – 2.67 (m, 3H), 2.13 – 2.05 (m, 1H), 1.95 – 1.85 (m, 2H), 1.69 – 1.42 (m, 6H).

HPP-9 inact. To a solution of HPI-1-F (18 mg, 36 µmol), **21** (15 mg, 36 µmol) and TBTA (5.7 mg, 10.5 µmol) in DMF (1 mL), a solution of $CuSO_4$ (5.7 mg, 36 µmol) and sodium ascorbate (4.8 mg, 24 µmol) in H_2O was added. The mixture was vigorously stirred at rt for 2h. The crude was purified by reverse phase chromatography (BGB Scorpius C18 4.5 g, H_2O + 0.1% TFA/CH_3CN + 0.1% TFA 95:5 to 10:90) to give **HPP-9 inactive** as a pale-yellow powder (27 mg, 83%). ^{1}H NMR (500 MHz, $CDCl_3$) δ 7.79 (d, *J* = 5.1 Hz, 1H), 7.66 (dd, *J* = 8.4, 7.3 Hz, 1H), 7.44 (dd, *J* = 7.2, 1.2 Hz, 1H), 7.26 – 6.68 (m, 9H), 6.37 (d, *J* = 7.6 Hz, 1H), 5.12 – 5.05 (m, 1H), 4.94 (dd, *J* = 12.4, 5.5 Hz, 1H), 4.40 (t, *J* = 7.1 Hz, 2H), 4.22 – 4.11 (m, 4H), 3.79 (d, *J* = 7.4 Hz, 3H), 3.54 (q, *J* = 5.6, 4.8 Hz, 3H), 3.31 (d, *J* = 7.4 Hz, 3H), 3.17 (s, 3H), 3.00 – 2.91 (m, 1H), 2.87 – 2.66 (m, 4H), 2.60 (ddd, *J* = 17.2, 4.3, 2.6 Hz, 2H), 2.38 – 2.32 (m, 3H), 2.12 – 2.04 (m, 1H), 1.98 (qd, *J* = 7.9, 2.3 Hz, 2H), 1.86 (h, *J* = 6.2, 5.7 Hz, 2H), 1.56 (td, *J* = 9.2, 8.5, 6.4 Hz, 2H), 1.41 (p, *J* =

7.7 Hz, 2H). ^{13}C NMR (126 MHz, $CDCl_3$) δ 198.6, 171.4, 169.0, 167.3, 166.1, 159.5, 159.1, 157.9, 157.30, 157.3, 156.6, 152.7, 148.5, 143.9, 143.7 136.7, 133.9, 129.9, 129.2, 128.4, 127.3, 123.7, 121.6, 120.9, 119.0, 115.9, 114.5, 113.0, 112.5, 110.9, 70.6, 69.1, 63.0, 61.1, 58.9, 55.3, 49.9, 41.6, 36.6, 33.4, 33.0, 31.9, 29.9, 28.5, 27.4, 26.0, 25.3, 19.4. ESI-HRMS (m/z): calcd. for $[C_{51}H_{56}N_6O_{11} + H]^+$ 915.3923; obsd. 915.3924.

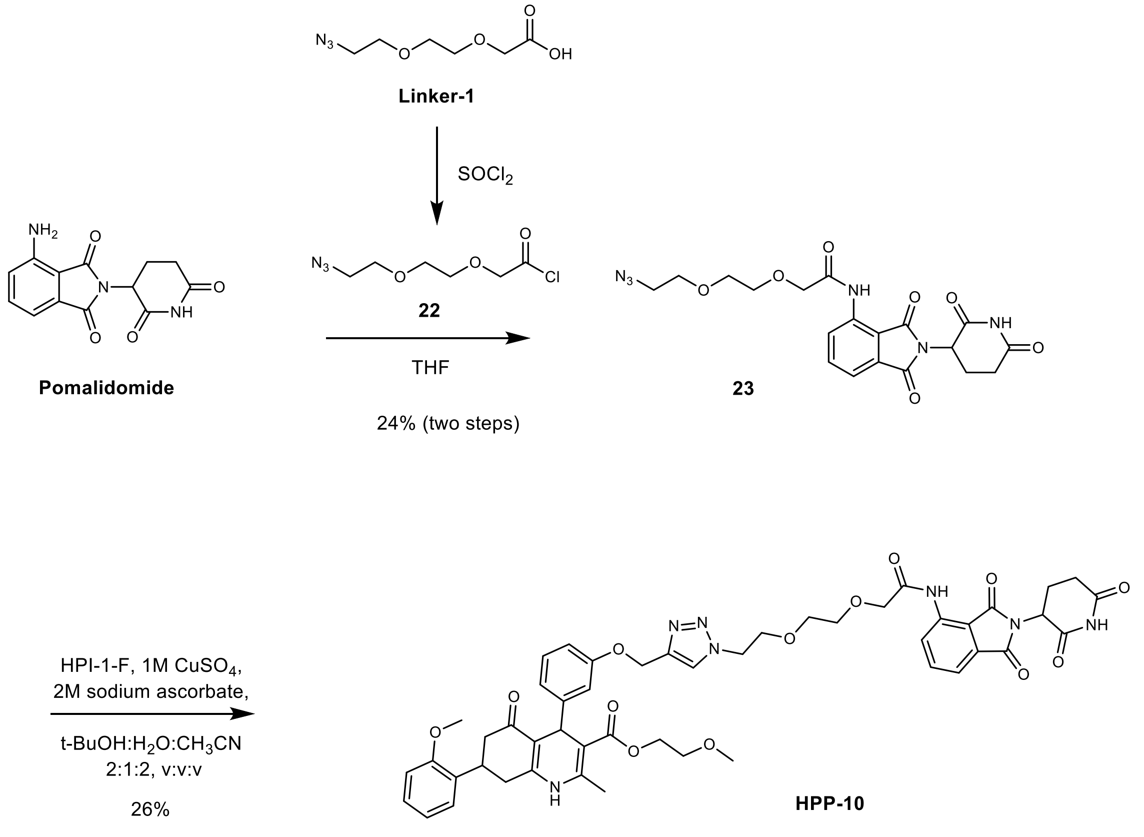

Supplementary Fig. 28. Synthesis of **HPP-10**

Compound 23. To a solution of **linker-1** (100 mg, 0.53 mmol) was added $SOCl_2$ (77 µL, 1.06 mmol). The reaction mixture was refluxed for 3 h, after which it was checked for completion by TLC (diluted in MeOH,-pentane/EtOAc 1:1, R_f = 0.53), then cooled to rt. The solution containing **22** was used directly for the next step without further purification. To a solution of **pomalidomide** (66 mg, 0.24 mmol) in anhydrous THF (1.0 mL) was added the solution containing **22** (110 mg, theoretical mass, 0.53 mmol). The reaction mixture was refluxed for 4 h, after which it was concentrated and extracted with CH_2Cl_2. The organic layer was washed with sat. $NaHCO_3$ aq., dried over $MgSO_4$ and concentrated. The resulting crude was purified by reverse phase chromatography (BGB Scorpius C18 4.5 g, H_2O + 0.1% TFA/CH_3CN + 0.1% TFA 80:20 to 30:70) to give **23** as an off-white solid (50 mg, 24% two steps).; R_f= 0.24

(CH_2Cl_2/MeOH 20:1). ^{1}H NMR (400 MHz, $CDCl_3$): δ 10.45 (br, 1H, NH), 8.88 (d, *J* = 8.5 Hz, 1H), 7.95 (br, 1H, NH), 7.73 (dd, *J* = 8.4, 7.3 Hz, 1H), 7.58 (d, *J* = 7.3 Hz, 1H), 5.03 – 4.88 (m, 1H), 4.22 (s, 2H), 3.88 – 3.68 (m, 6H), 3.39 (t, *J* = 5.2 Hz, 2H), 2.99 – 2.66 (m, 3H), 2.23 – 2.11 (m, 1H). LC-MS (ES$^+$): m/z 444.89 $[M+H]^+$.

HPP-10. To a solution of compound **23** (13 mg, 0.029 mmol) and **HPI-1-F** (15 mg, 0.029 mmol) in degassed *t*-BuOH/CH_3CN/H_2O (1.5 mL, 2:2:1) were added degassed aqueous solutions of 2 M sodium ascorbate (150 µL, 0.29 mmol) and 1 M $CuSO_4$ (15 µL, 0.015 mmol, 50 mol%) and the mixture was stirred overnight at rt. CH_2Cl_2 and H_2O were added to the reaction mixture and after partitioning of the layers, the aqueous layer was extracted with CH_2Cl_2. The organic layer was dried over $MgSO_4$ and concentrated. The resulting crude was purified by reverse phase chromatography (BGB Scorpius C18 4.5 g, H_2O + 0.1% TFA/CH_3CN + 0.1% TFA 75:25 to 30:70) to give **HPP-10** as an off-white solid (5 mg, 26%).; R_f= 0.13 (CH_2Cl_2/MeOH 20:1). ^{1}H NMR (500 MHz, $CDCl_3$): δ 10.42 (br, 1H, NH), 8.83 (d, *J* = 8.4 Hz, 1H), 8.75 (br, 1H, NH), 7.80 (s, 1H), 7.72 – 7.64 (m, 1H), 7.55 (d, *J* = 7.3 Hz, 1H), 7.26 – 6.62 (m, 8H), 6.34 (s, 1H), 5.13 (s, 3H), 4.96 (m, 1H), 4.62 – 4.43 (m, 2H), 4.15 (m, 4H), 3.96 (m, 2H), 3.82 – 3.71 (m, 7H), 3.64 – 3.55 (m, 1H), 3.52 (t, *J* = 4.9 Hz, 2H), 3.29 (s, 3H), 2.90 – 2.51 (m, 7H), 2.36 (s, 3H), 2.21 – 2.09 (m, 1H). ^{13}C NMR (126 MHz, $CDCl_3$): δ 195.9, 171.2, 169.0, 168.5, 168.2, 168.1, 167.3, 166.7, 158.1, 157.2, 149.9, 148.8, 144.3, 144.1, 136.6, 136.4, 131.4, 130.5, 128.9, 128.0, 127.1, 125.2, 124.3, 121.4, 120.8, 118.9, 116.2, 114.4, 112.6, 110.7, 105.6, 71.7, 70.9, 70.5, 70.4, 69.9, 62.9, 61.6, 58.8, 55.2, 50.3, 49.3, 42.4, 36.5, 33.2, 32.9, 31.4, 22.7, 19.4. LC-MS (ES+): m/z 473.84 $[M+2H]^{2+}$ and m/z 946.14 $[M+H]^+$. ESI-HRMS (m/z): calcd. for $[C_{49}H_{51}N_7O_{13} + H]^+$ 946.3618; obsd. 946.3616.

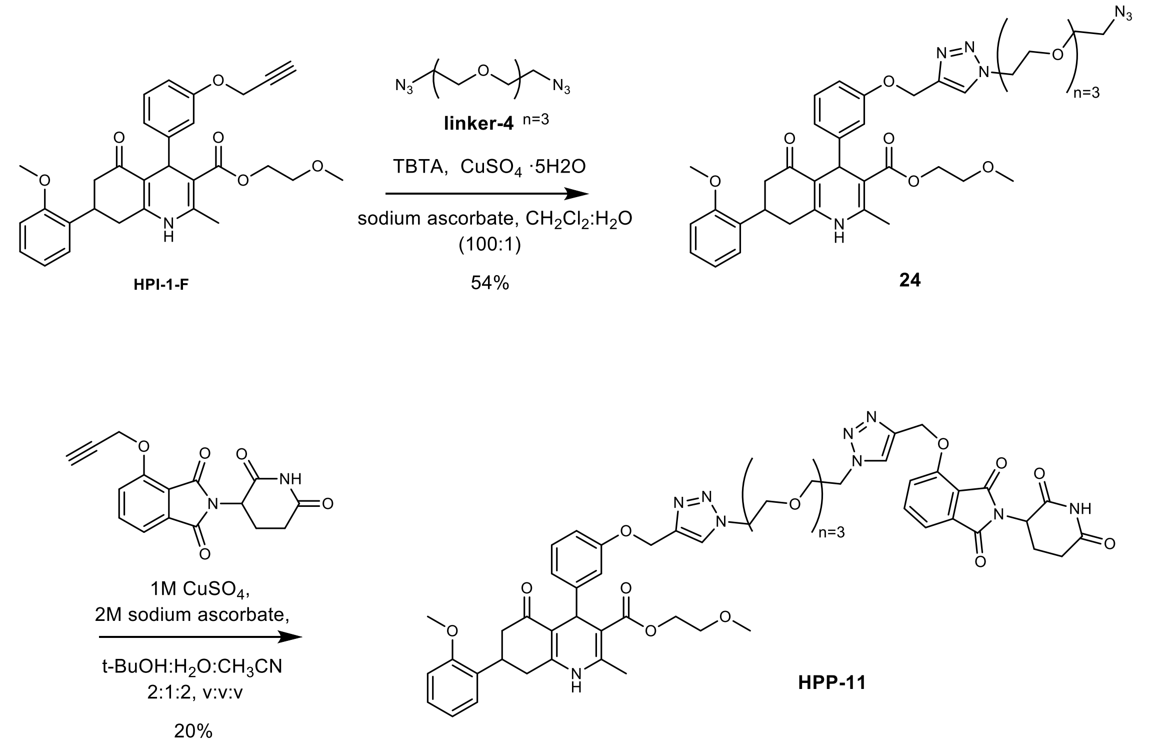

Supplementary Fig. 29. Synthesis of **HPP-11**

Compound 24. To a solution of **HPI-1-F** (12 mg, 24 µmol), **linker-4** (60 mg, 245 µmol, 10eq) and TBTA (3.8 mg, 7 µmol) in CH_2Cl_2 (1.0 mL), a solution of $CuSO_4$ (3.8 mg, 24 µmol) and sodium ascorbate (4.8 mg, 24 µmol) in H_2O (50 µL) was added. The mixture was vigorously stirred at rt and under nitrogen atmosphere for 2 h. The crude was purified by reverse phase chromatography (BGB Scorpius C18 4.5 g, H_2O + 0.1% TFA/CH_3CN + 0.1% TFA 95:5 to 10:90) to give **24** as a yellow oil (9.7 mg, 54%).

HPP-11. To a solution of compound **24** (14 mg, 0.019 mmol) and compound **10** (6 mg, 0.019 mmol) in degassed *t*-BuOH/CH_3CN/H_2O (1.0 mL, 2:2:1) were added degassed aqueous solutions of 2 M sodium ascorbate (19 µL, 0.039 mmol) and 1 M $CuSO_4$ (4 µL, 0.004 mmol, 20 mol%) and the mixture was stirred overnight at rt. CH_2Cl_2 and H_2O were added to the reaction mixture and after partitioning of the layers, the aqueous layer was extracted with CH_2Cl_2. The organic layer was dried over $MgSO_4$ and concentrated. The resulting crude was purified by PTLC (SiO_2, CH_2Cl_2/MeOH 15:1, eluted once) to give **HPP-11** as an off-white solid (4 mg, 20%). 10 mg of **HPP-11** were further purified by reverse phase HPLC. Semi-preparative HPLC runs were carried out with a gradient from 30% to 70% acetonitrile/water system (0.1% TFA) for 55 min and a flow of 3 mL/min, monitored by a PDA detector at 254 nm and 360 nm. The fraction was collected and lyophilized to afford 8.2 mg of the product; R_f= 0.35 (CH_2Cl_2/MeOH 10:1.^{1}H NMR (500 MHz, $CDCl_3$): δ 8.44 (br, 1H, NH), 7.90 (s, 1H), 7.83 (s, 1H), 7.67

– 7.62 (m, 1H), 7.51 (dd, *J* = 8.4, 2.9 Hz, 1H), 7.45 (d, *J* = 7.2 Hz, 1H), 7.24 – 6.70 (m, 8H), 6.36 (br, 1H, NH), 5.41 (s, 2H), 5.18 – 5.07 (m, 3H), 4.92 (m, 1H), 4.54 – 4.45 (m, 4H), 4.16 (m, 2H), 3.86 (t, *J* = 5.1 Hz, 2H), 3.83 – 3.73 (m, 5H), 3.58 – 3.49 (m, 11H), 3.30 (s, 3H), 2.88 – 2.51 (m, 7H), 2.35 (s, 3H), 2.10 (m, 1H). ^{13}C NMR (126 MHz, $CDCl_3$): δ 195.6, 171.0, 168.2, 168.2, 167.3, 166.9, 165.7, 158.2, 157.1, 155.7, 149.6, 148.8, 144.2, 142.8, 136.7, 133.7, 130.5, 128.9, 128.0, 127.1, 124.7, 124.2, 121.3, 120.7, 120.1, 116.5, 114.5, 112.3, 110.7, 105.5, 70.5, 70.5, 70.3, 69.3, 69.2, 63.2, 62.9, 61.7, 58.8, 55.2, 50.3, 50.2, 49.1, 42.5, 36.4, 33.2, 32.9, 31.4, 22.6, 19.4. LC-MS (ES$^+$): m/z 529.67 $[M+2H]^{2+}$ and m/z 1058.25 $[M+H]^+$. ESI-HRMS (m/z): calcd. for $[C_{54}H_{59}N_9O_{14} + H]^+$ 1058.4254; obsd. 1058.4261.

3. NMR Spectra

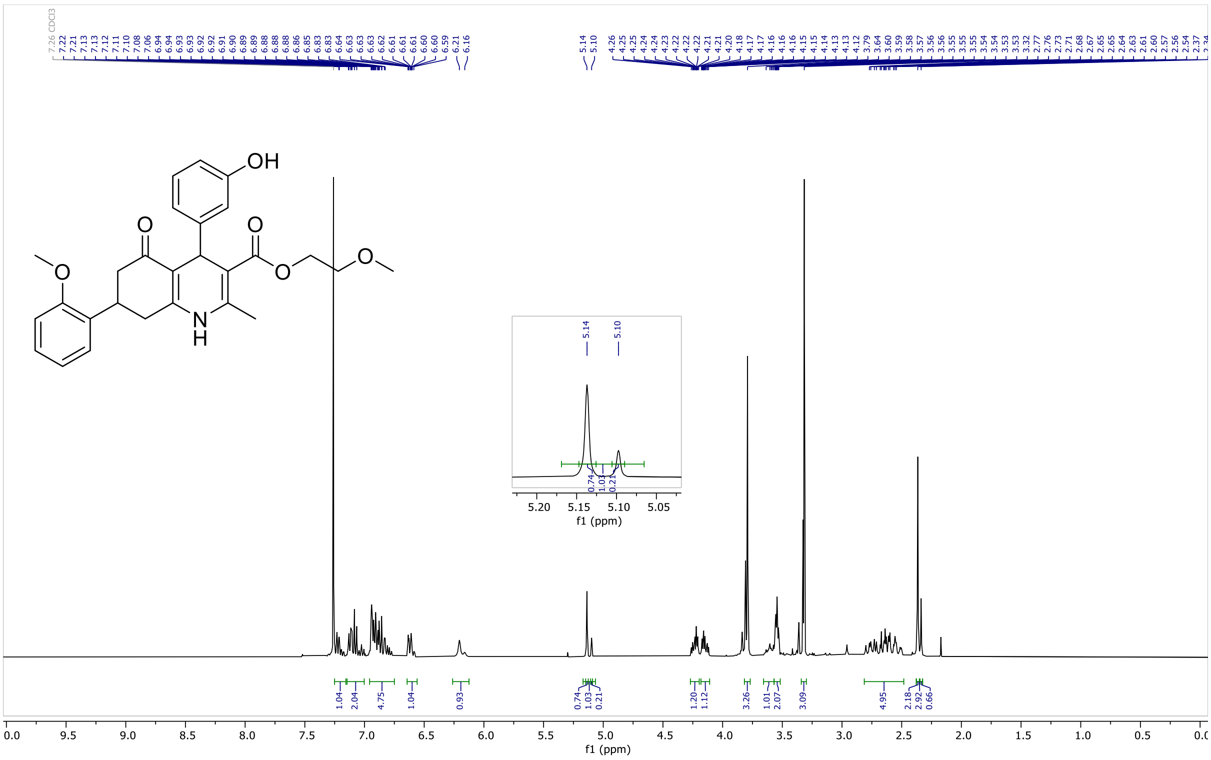

Supplementary Fig. 30. ^{1}H NMR (400 MHz, $CDCl_3$) of **HPI-1**. Diastereomeric pair ratio 3.4:1.

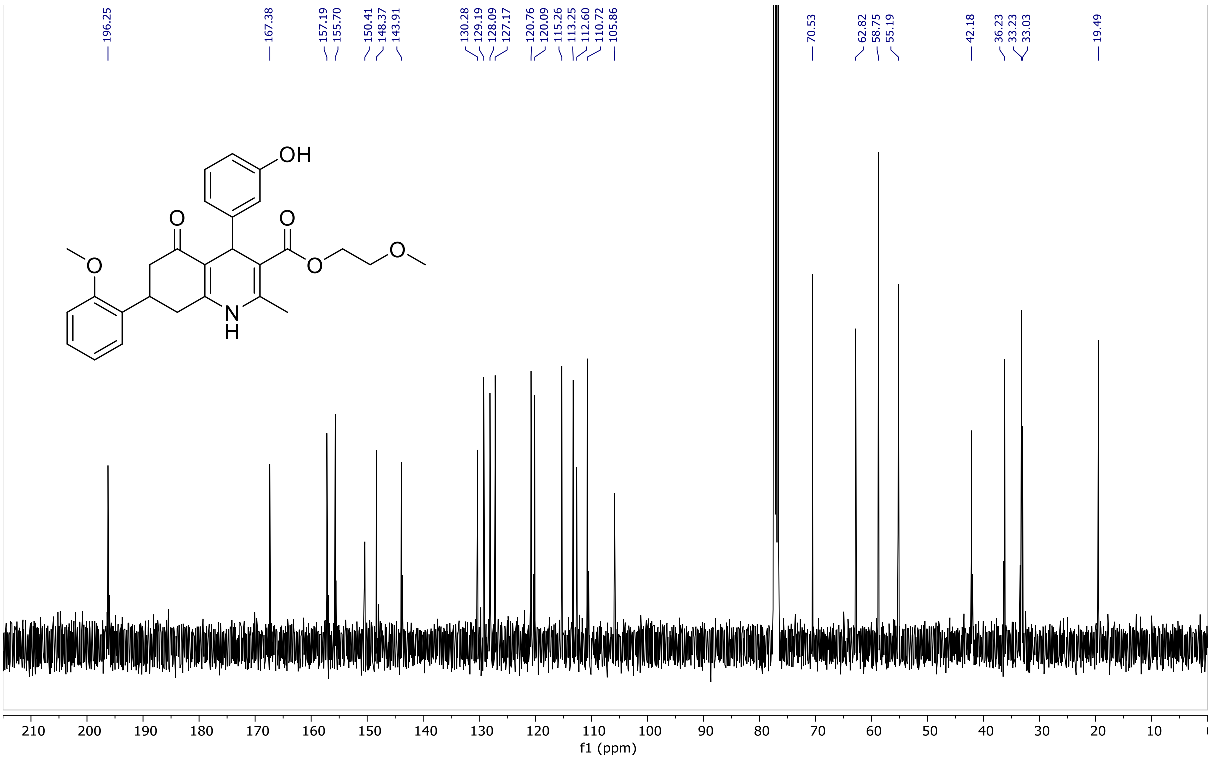

Supplementary Fig. 31. ^{13}C NMR (101 MHz, $CDCl_3$) of **HPI-1**.

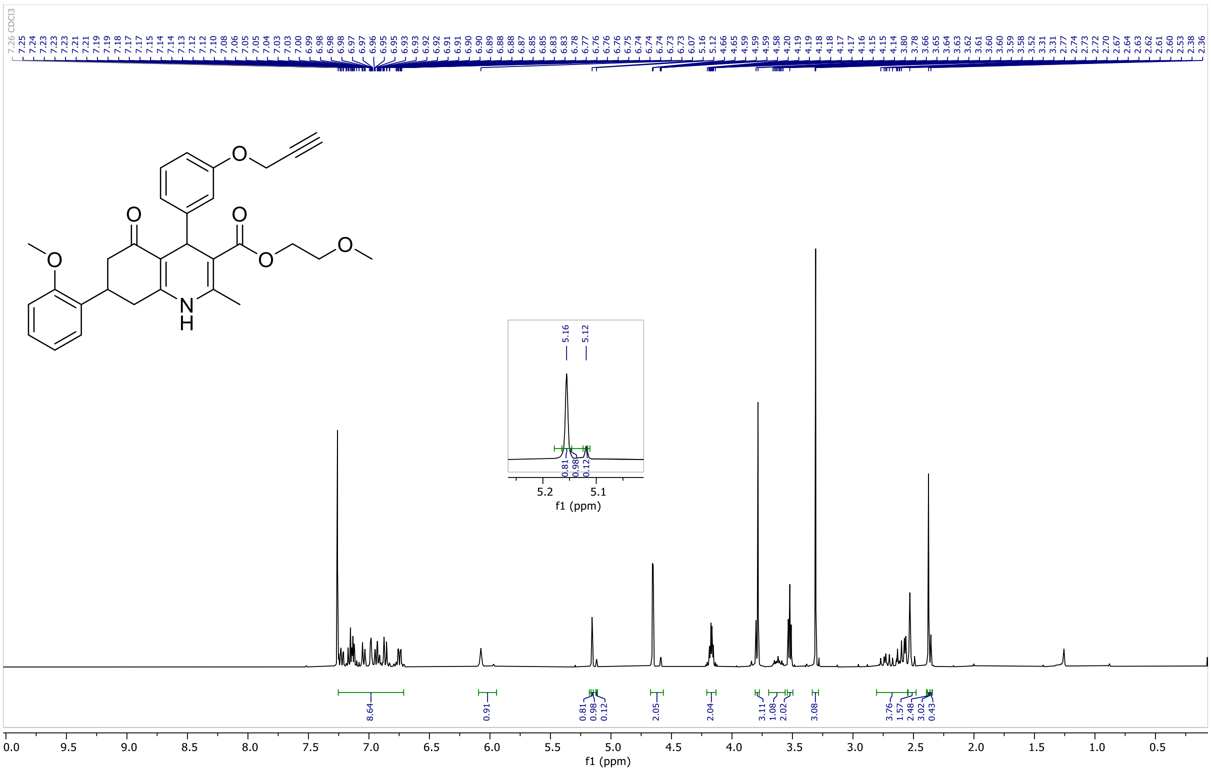

Supplementary Fig. 32. ^{1}H NMR (400 MHz, $CDCl_3$) of **HPI-1-F**. Diastereomeric pair ratio 6.7:1.

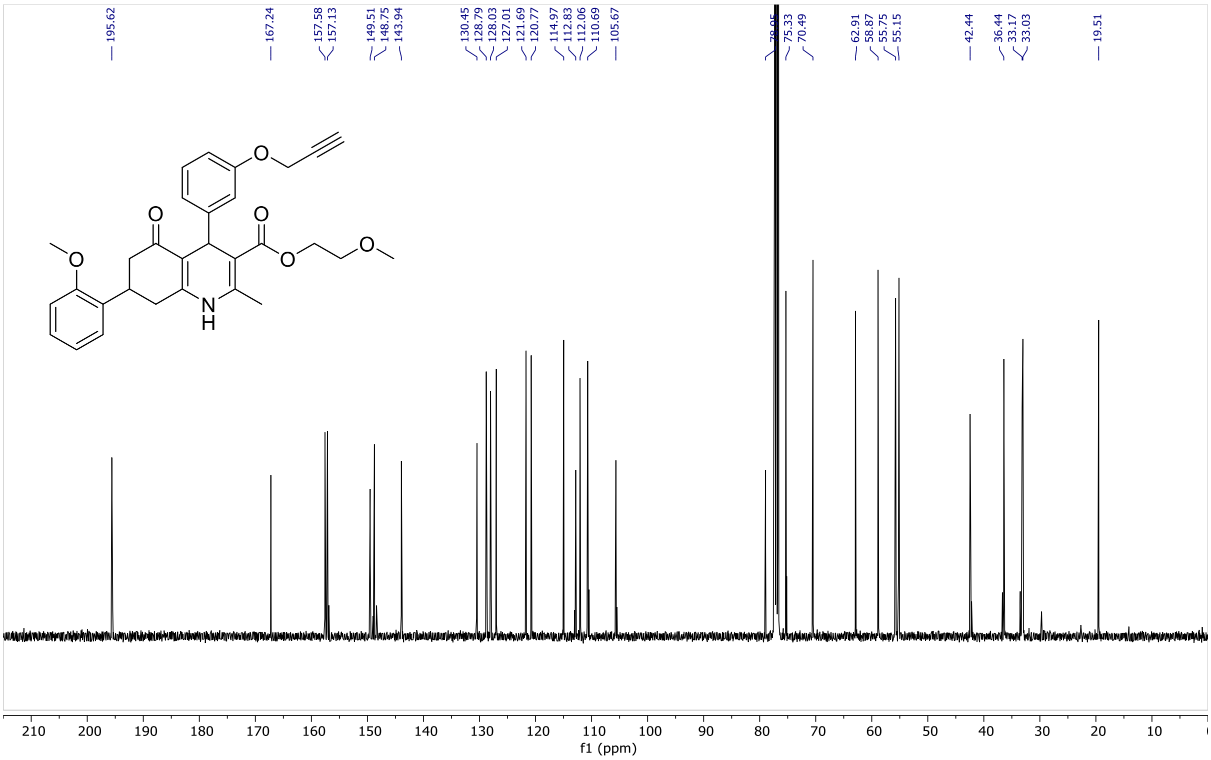

Supplementary Fig. 33. ^{13}C NMR (101 MHz, $CDCl_3$) of **HPI-1-F**.

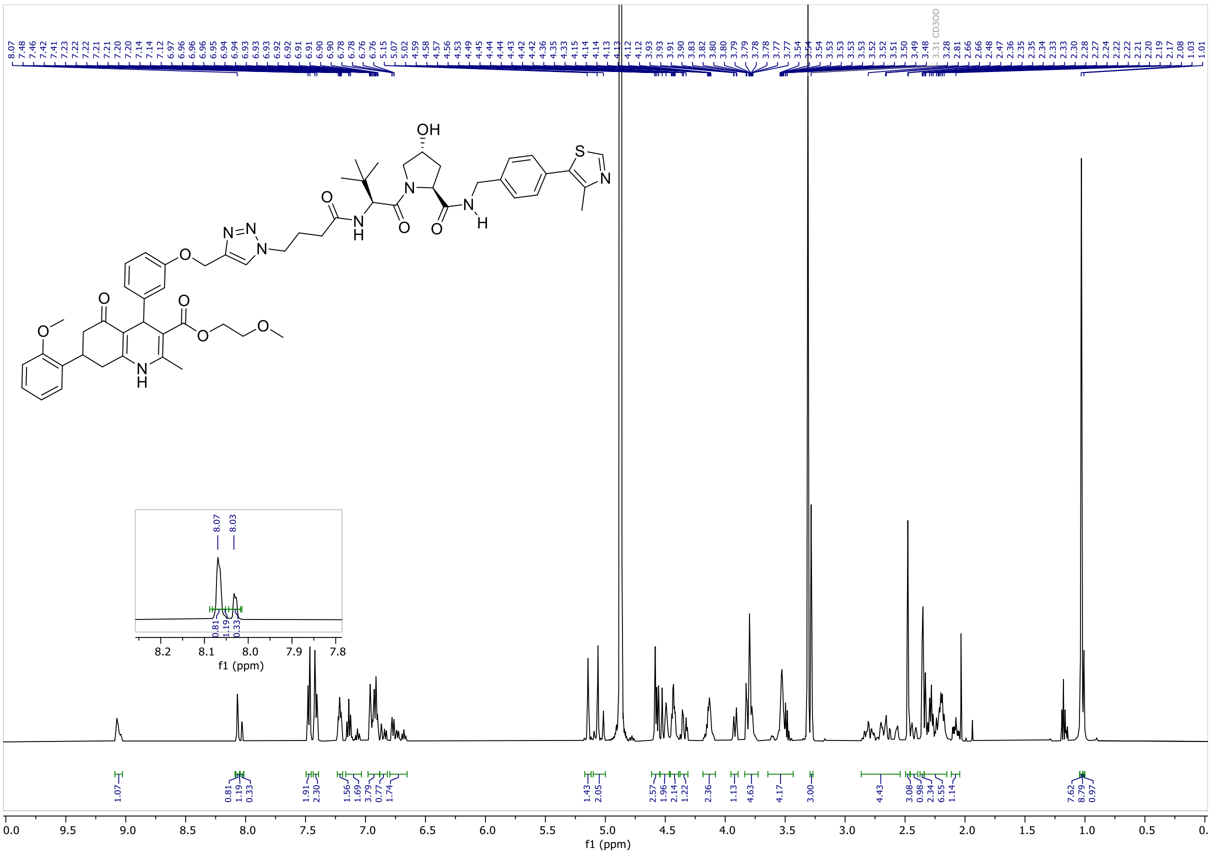

Supplementary Fig. 34. ^{1}H NMR (500 MHz, CD_3OD) of **HPP-1**. Diastereomeric pair ratio 3.9:1.

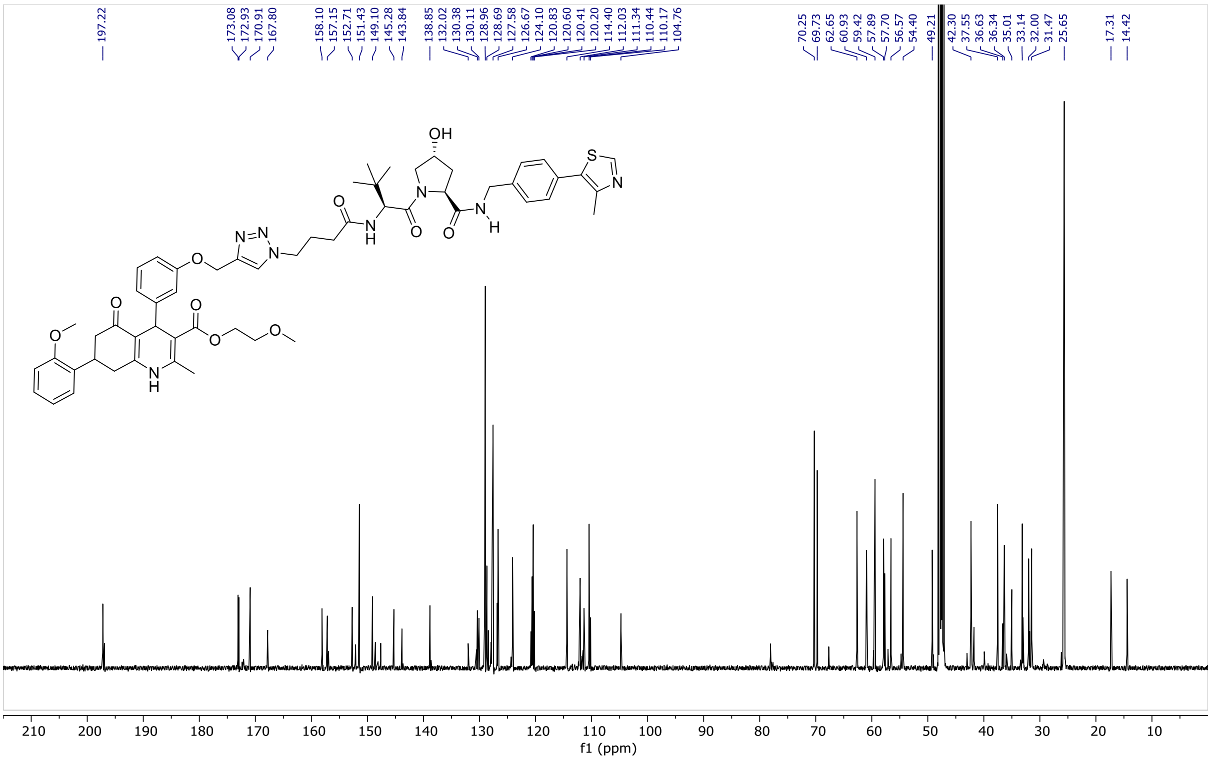

Supplementary Fig. 35.^{13}C NMR (126 MHz, CD_3OD) of **HPP-1**.

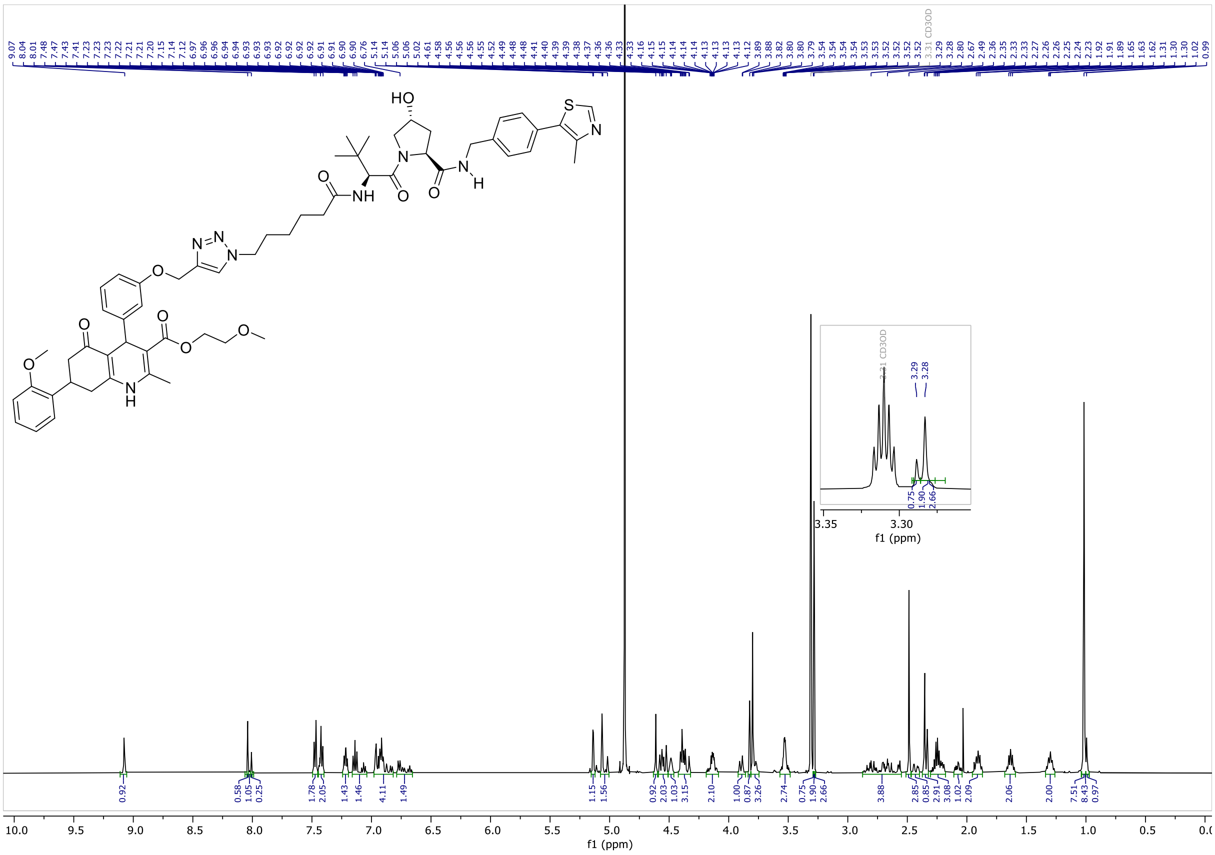

Supplementary Fig. 36. ^{1}H NMR (500 MHz, CD_3OD) of **HPP-2**. Diastereomeric pair ratio 5.2:1.

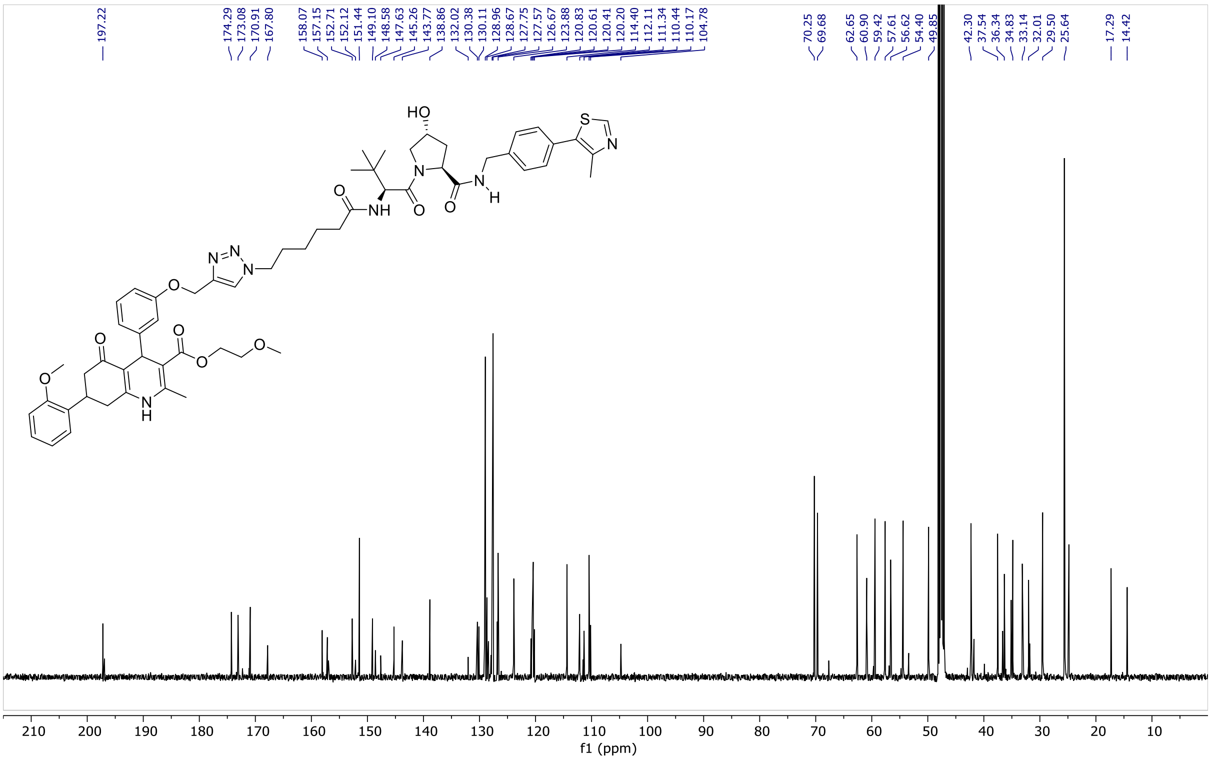

Supplementary Fig. 37. ^{13}C NMR (126 MHz, CD_3OD) of compound **HPP-2**.

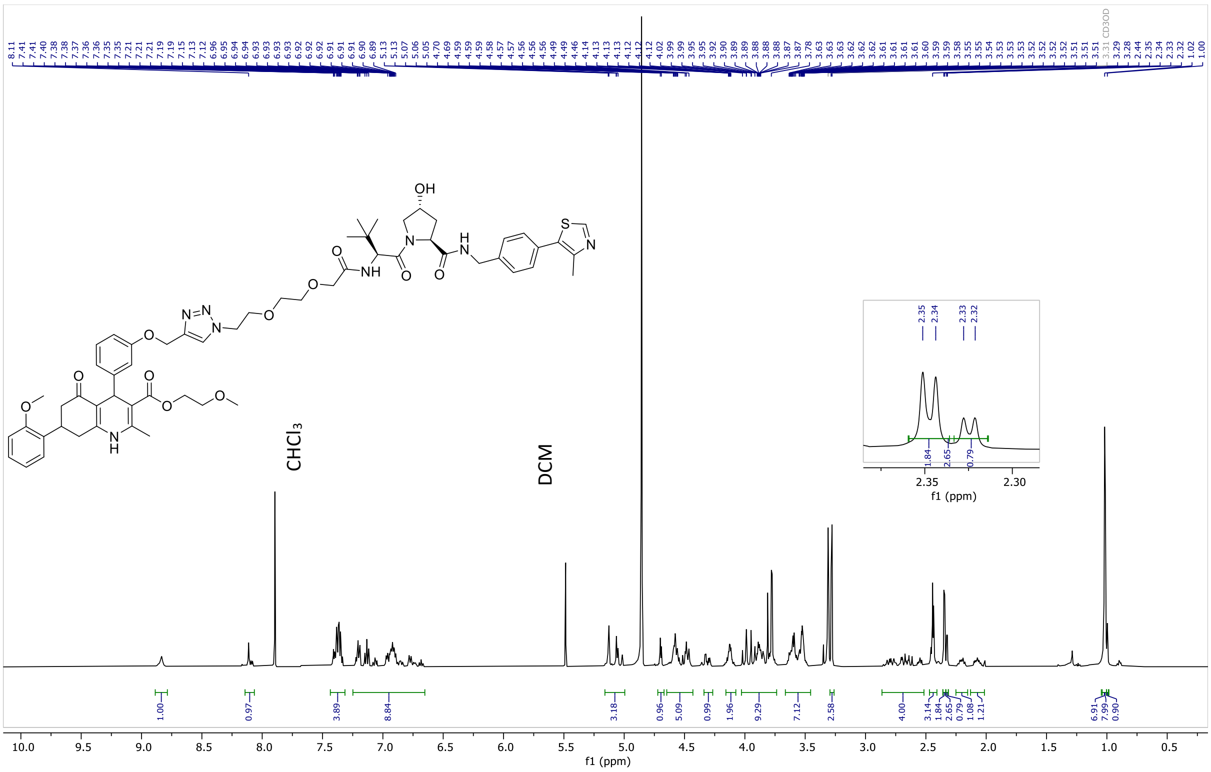

Supplementary Fig. 38. ^{1}H NMR (500 MHz, CD_3OD) of **HPP-3**. Diastereomeric pair ratio 4:1.

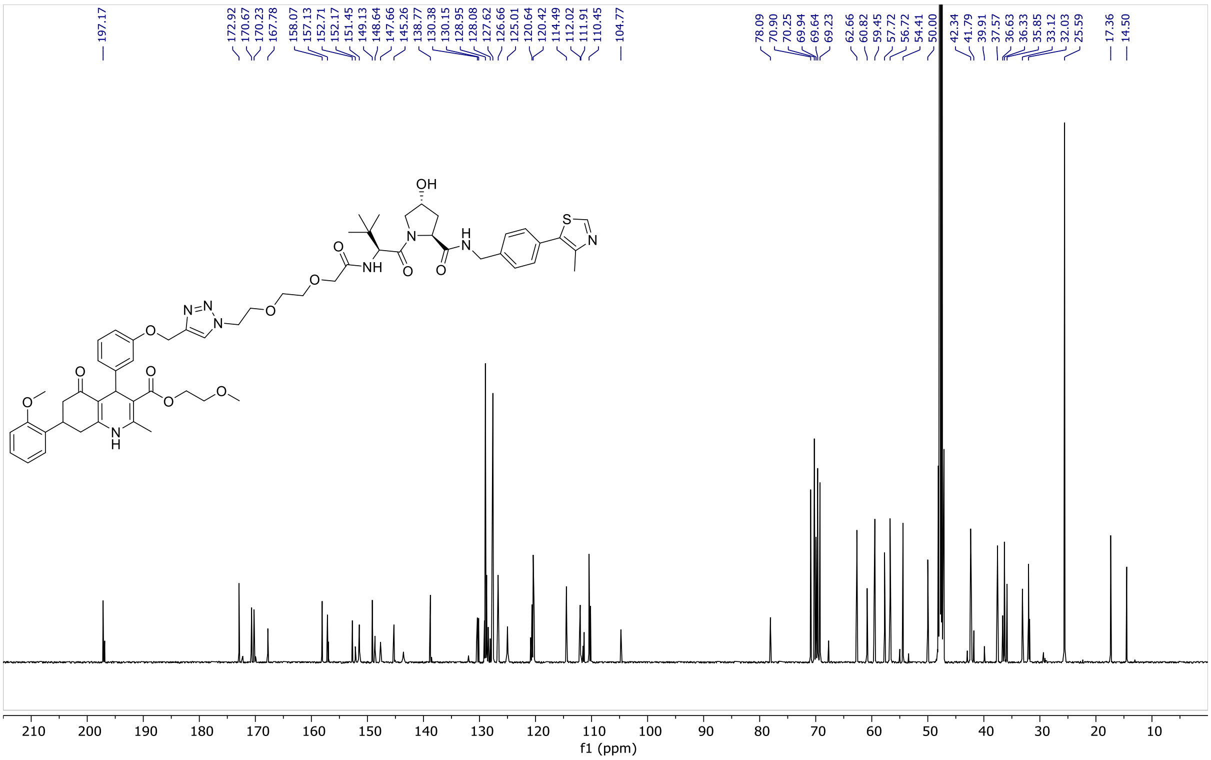

Supplementary Fig. 39. ^{13}C NMR (126 MHz, CD_3OD) of **HPP-3**.

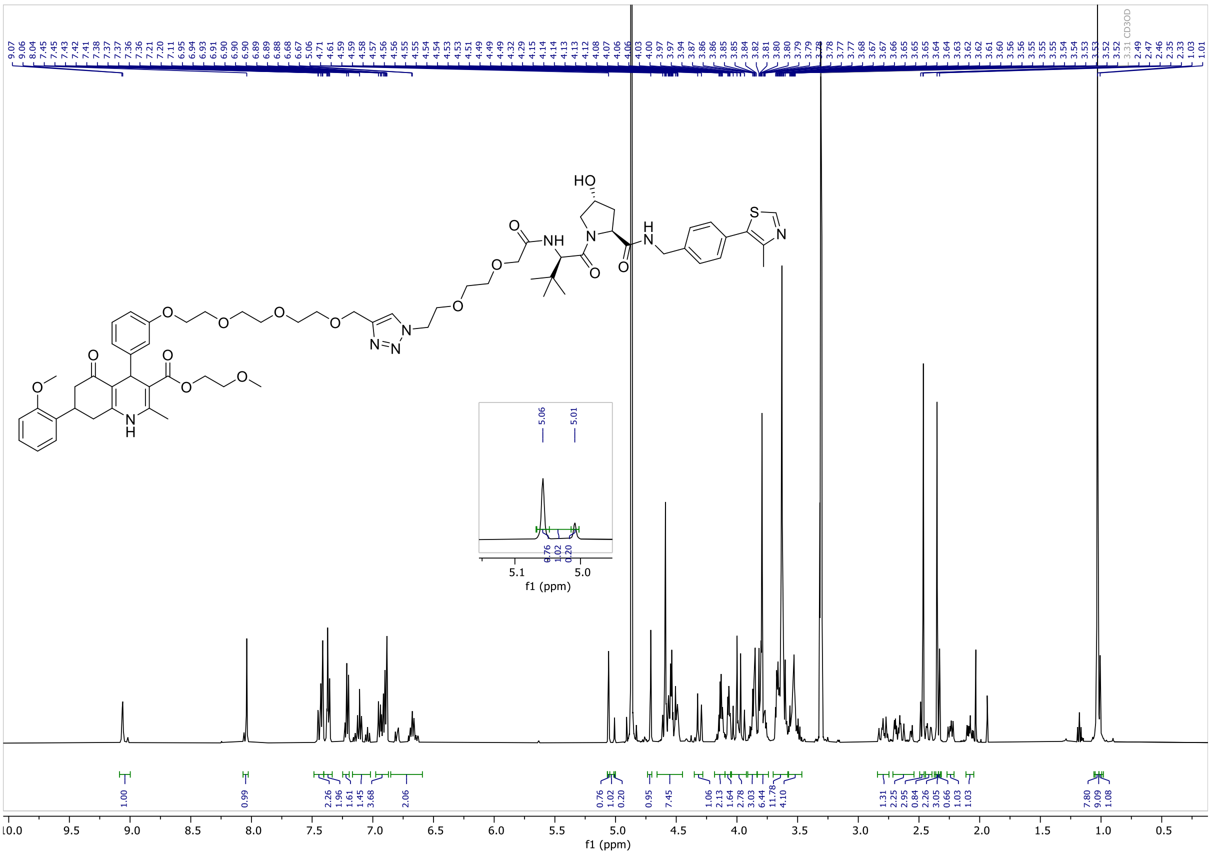

Supplementary Fig. 40. ^{1}H NMR (400 MHz, CD_3OD) of **HPP-4**. Diastereomeric pair ratio 4.4:1.

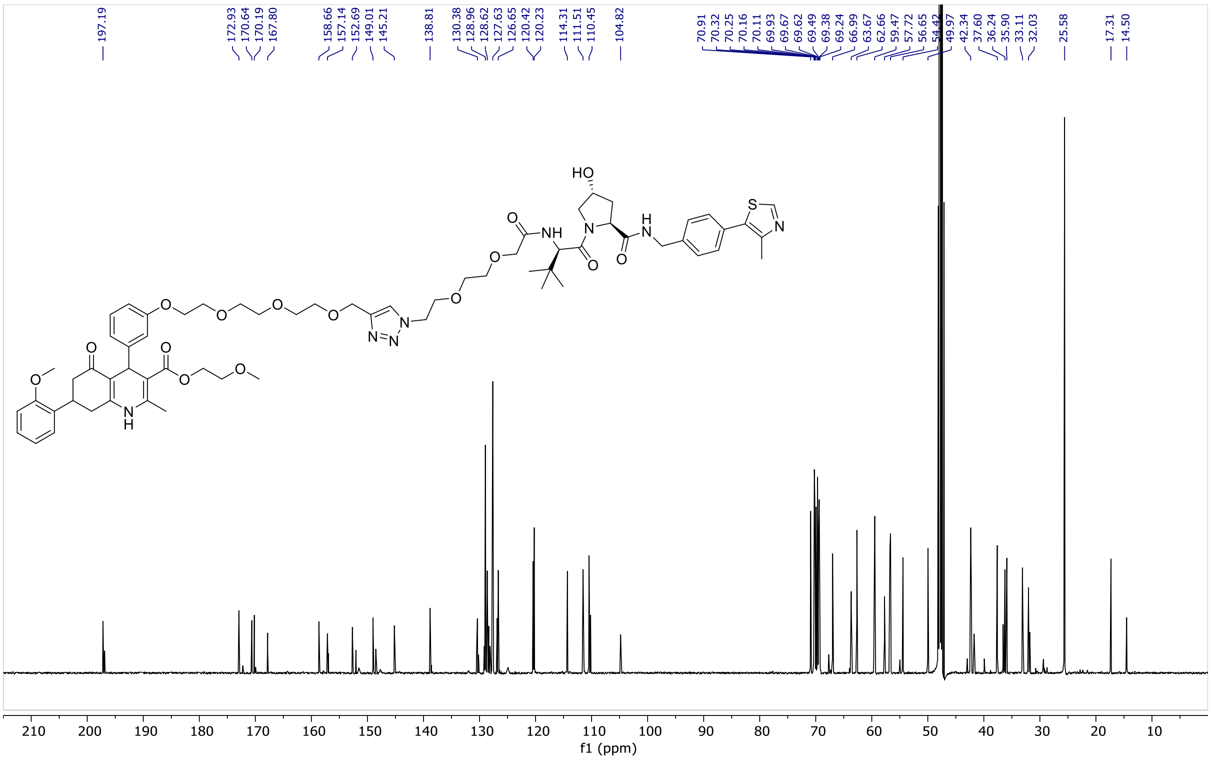

Supplementary Fig. 41. ^{13}C NMR (126 MHz, CD_3OD) of compound **HPP-4**.

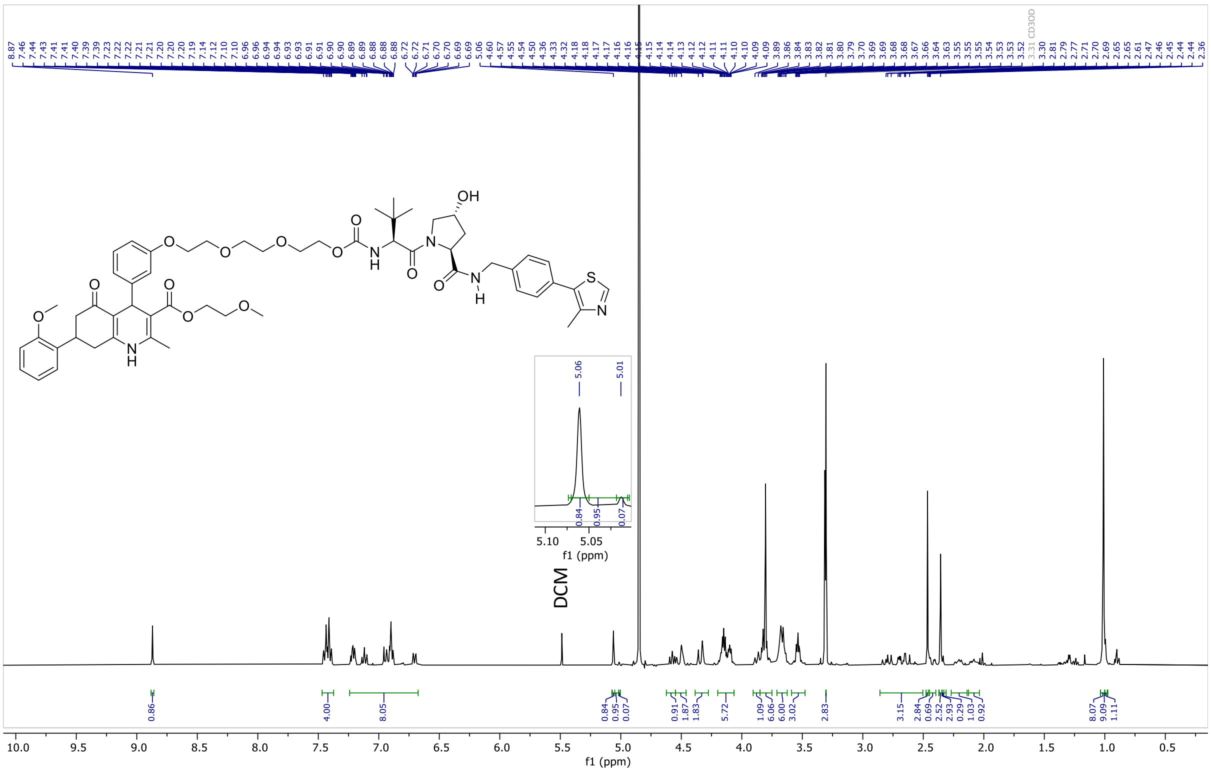

Supplementary Fig. 42. ^{1}H NMR (500 MHz, CD_3OD) of **HPP-5**. Diastereomeric pair ratio 8.7:1.

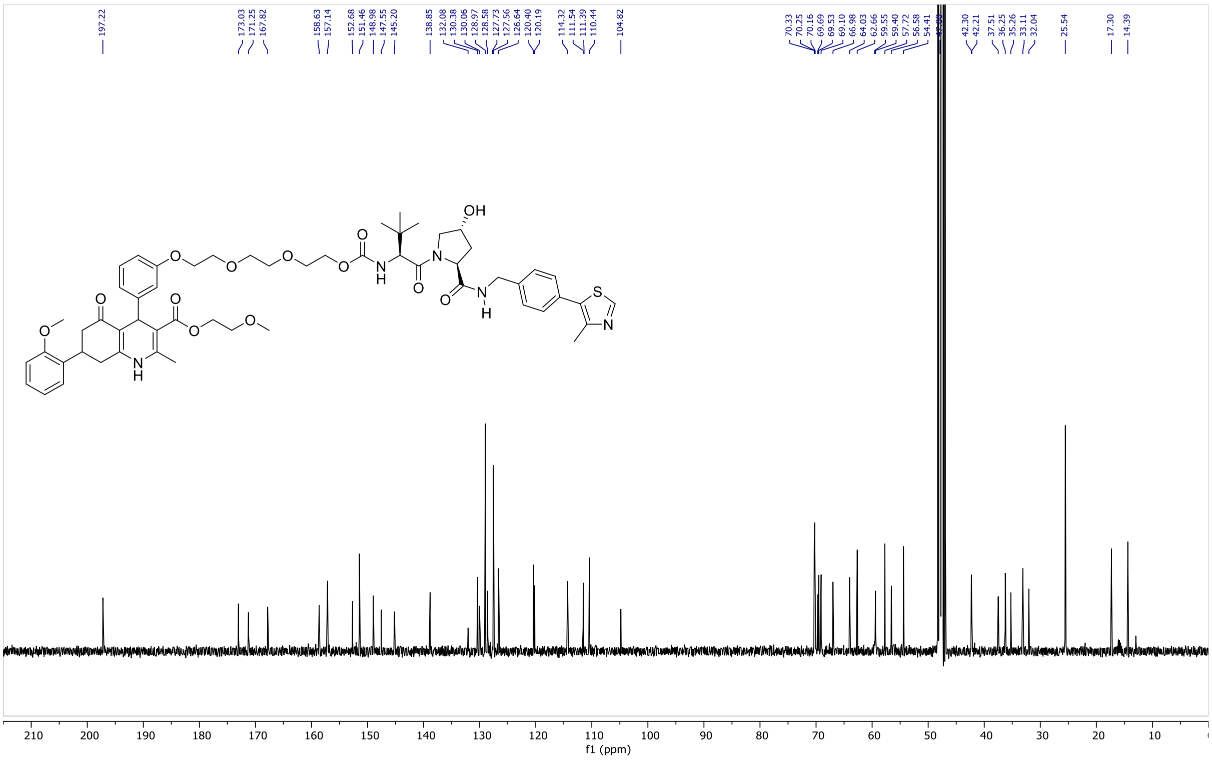

Supplementary Fig. 43. ^{13}C NMR (126 MHz, CD_3OD) of **HPP-5**.

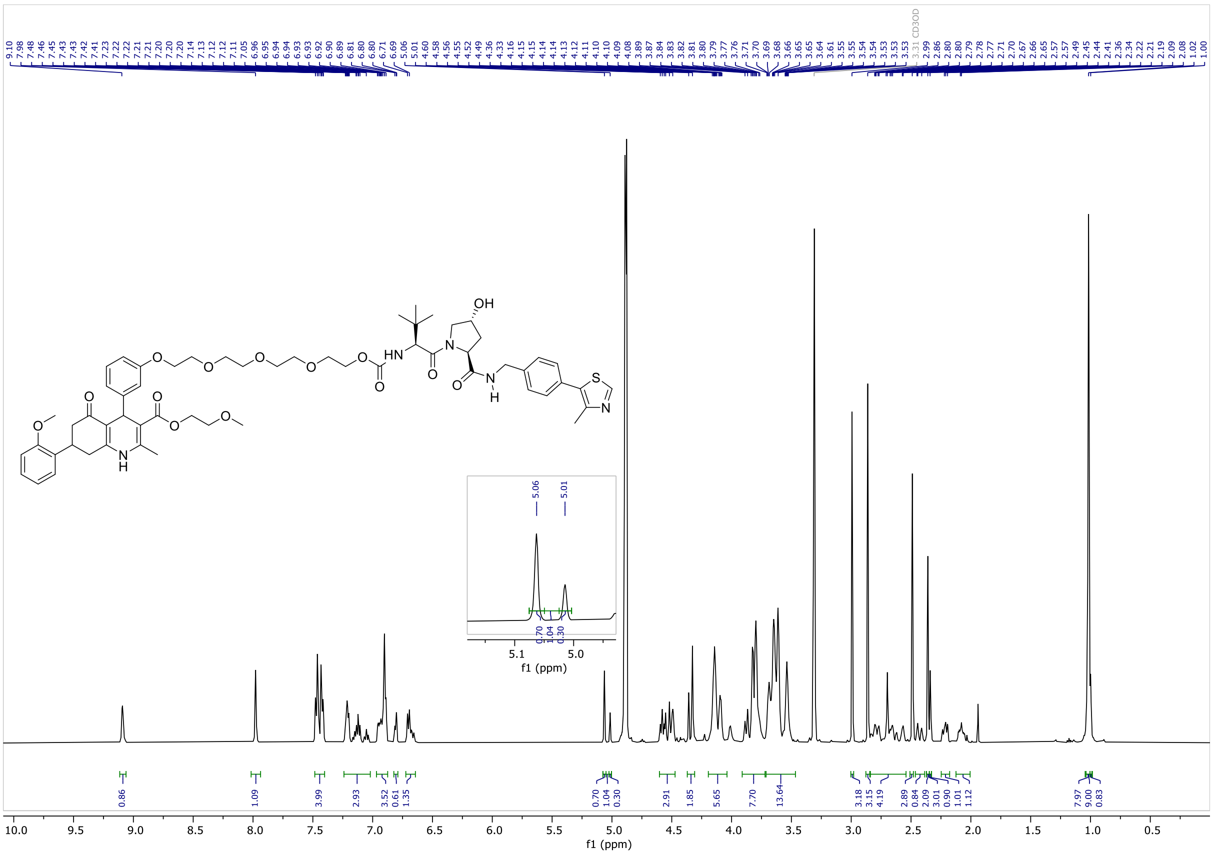

Supplementary Fig. 44. ^{1}H NMR (500 MHz, CD_3OD) of **HPP-6**. Diastereomeric pair ratio 4:1.

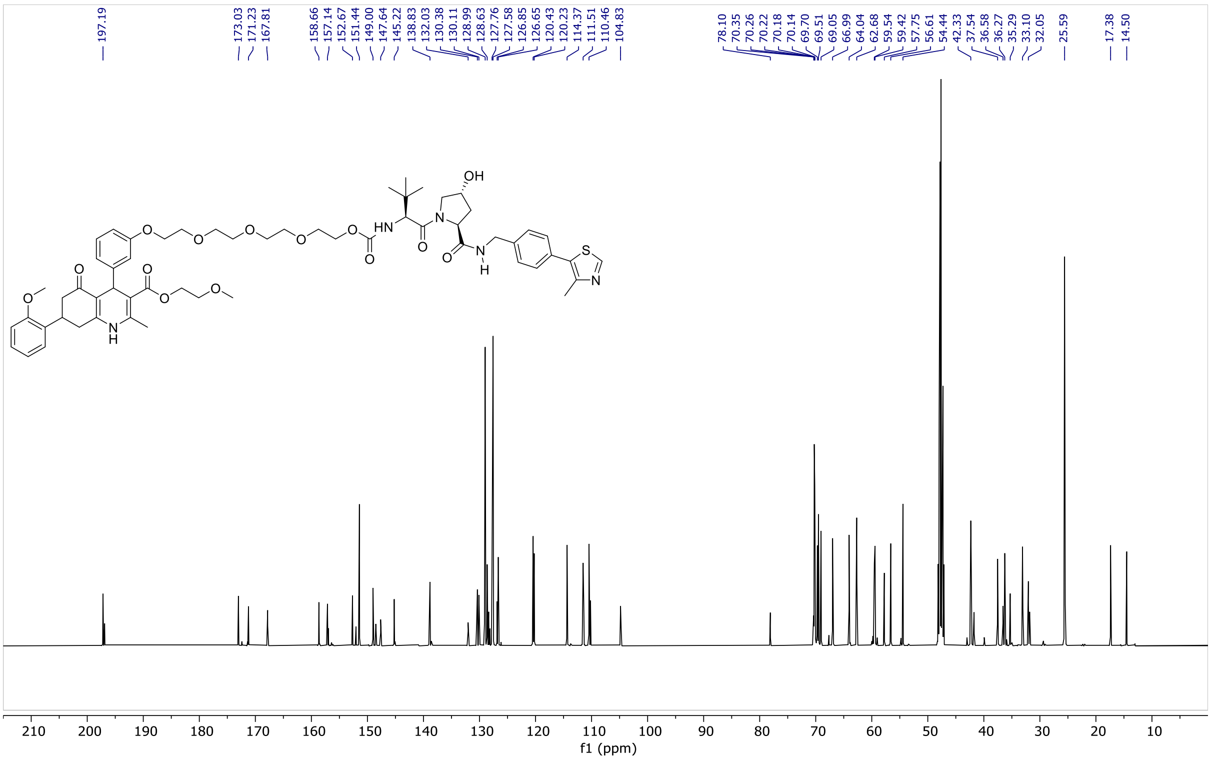

Supplementary Fig. 45. ^{13}C NMR (126 MHz, CD_3OD) of **HPP-6**.

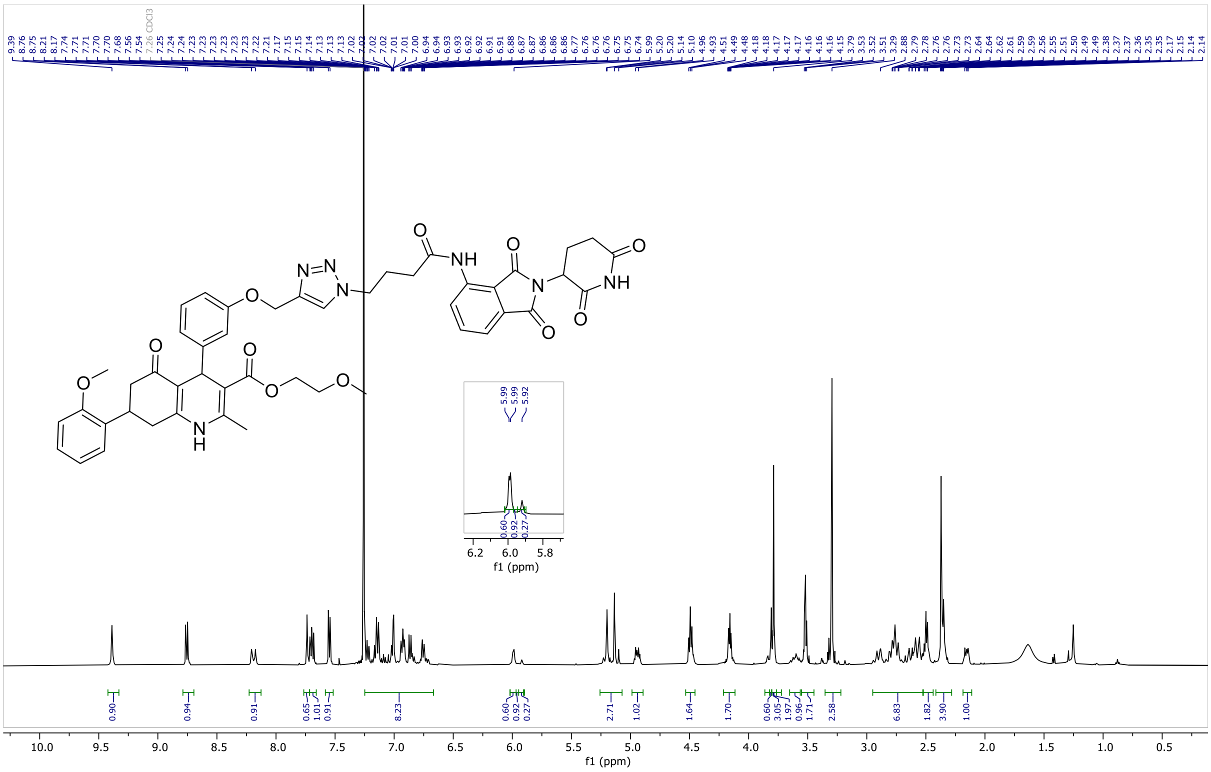

Supplementary Fig. 46. ^{1}H NMR (500 MHz, $CDCl_3$) of **HPP-7**. Diastereomeric pair ratio 3:1.

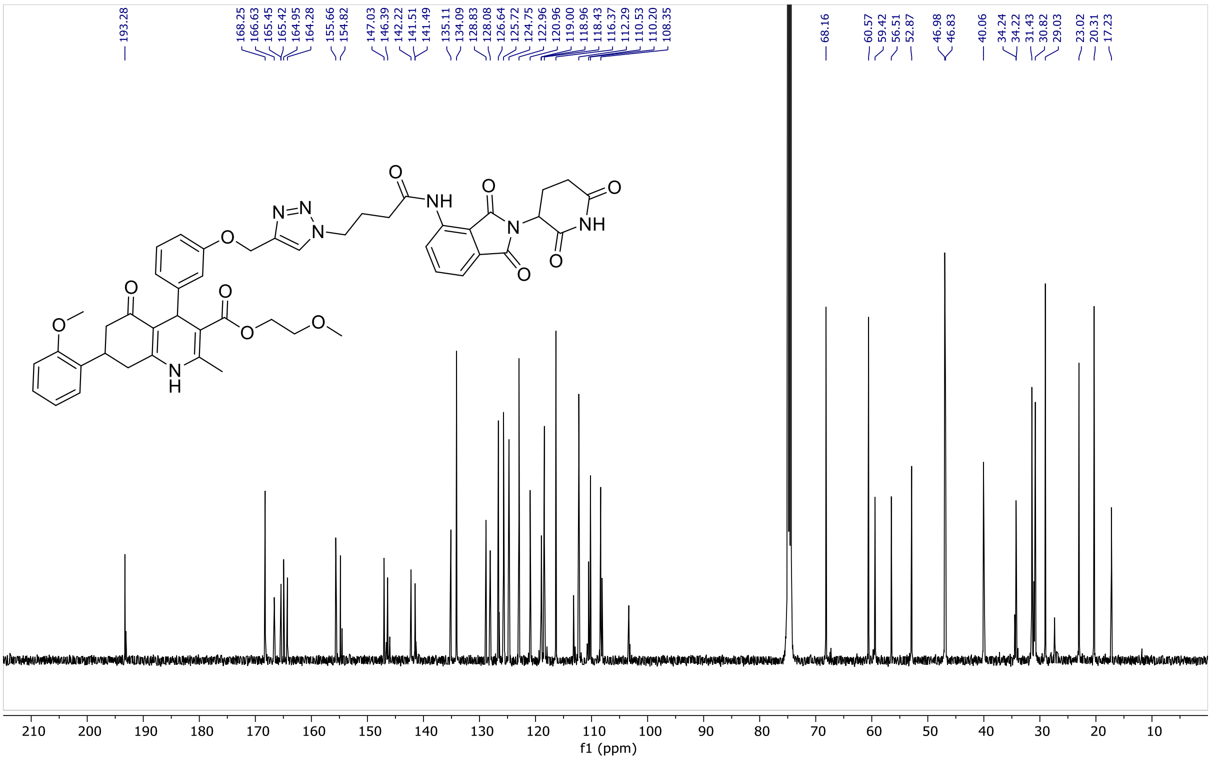

Supplementary Fig. 47.^{13}C NMR (126 MHz, $CDCl_3$) of **HPP-7**.

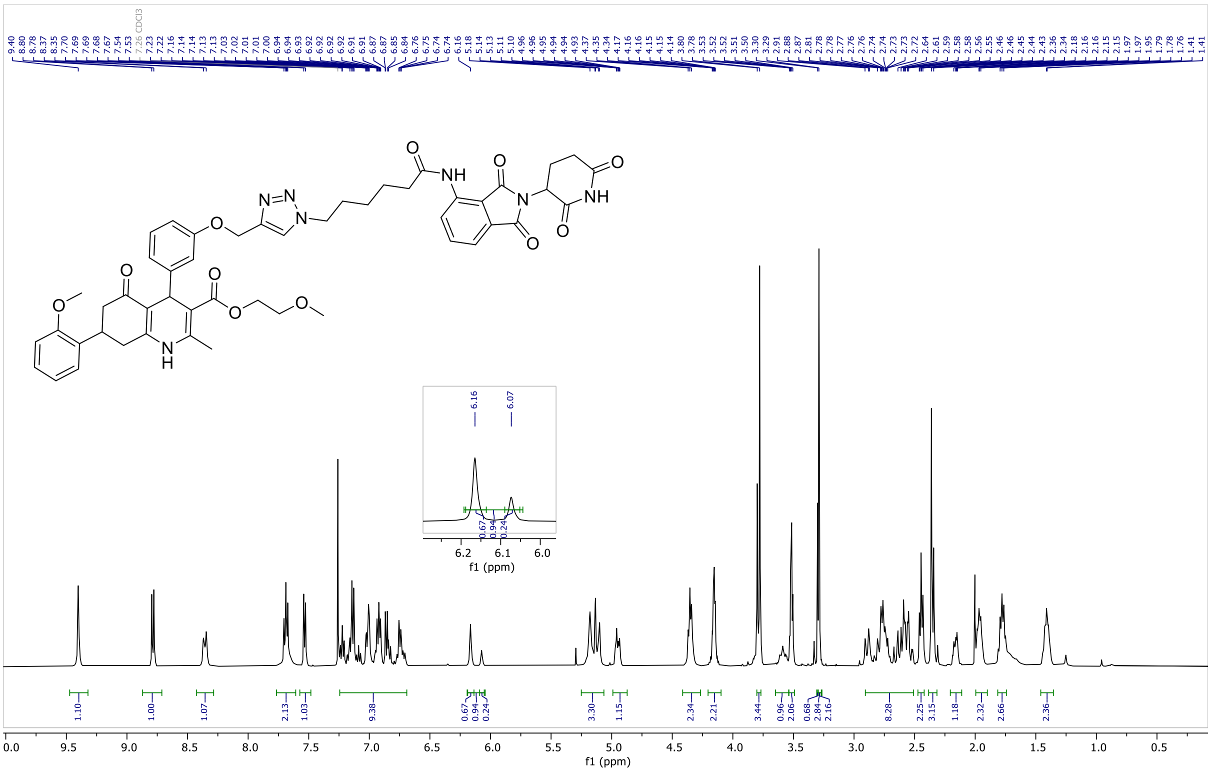

Supplementary Fig. 48. ^{1}H NMR (500 MHz, $CDCl_3$) of **HPP-8**. Diastereomeric pair ratio 3:1.

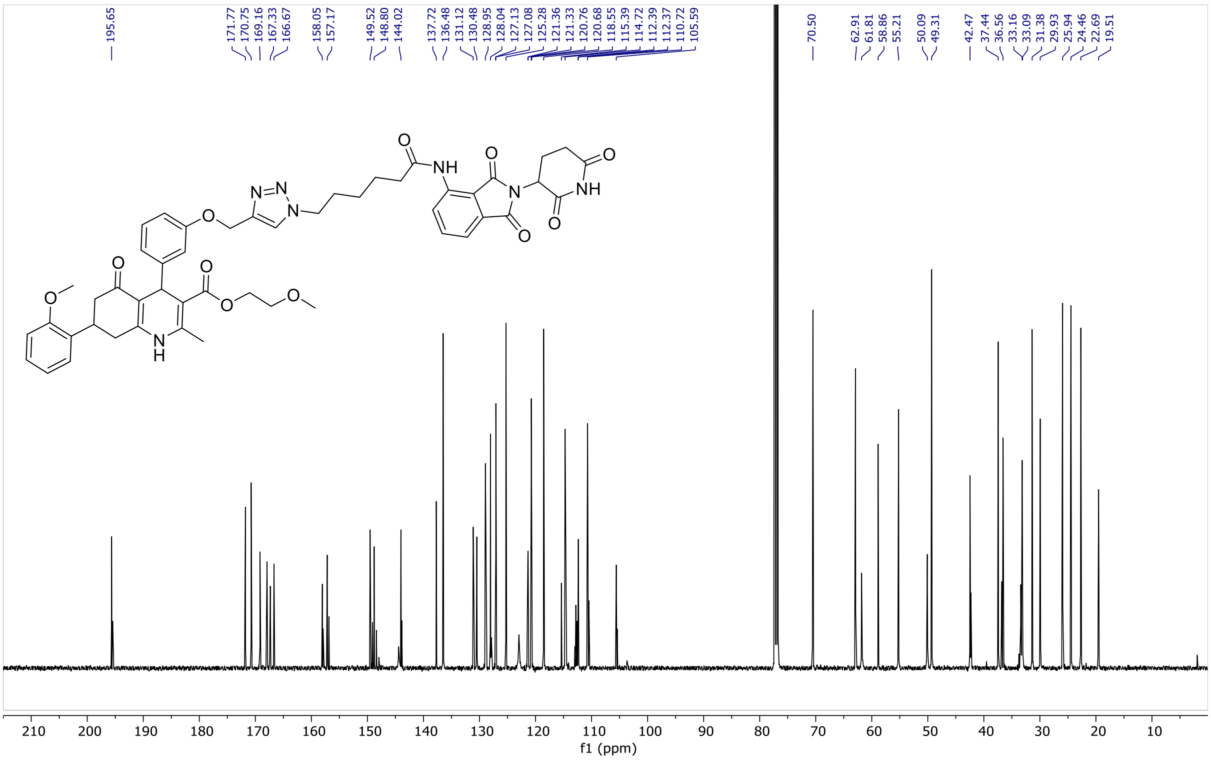

Supplementary Fig. 49. ^{13}C NMR (126 MHz, $CDCl_3$) of **HPP-8**.

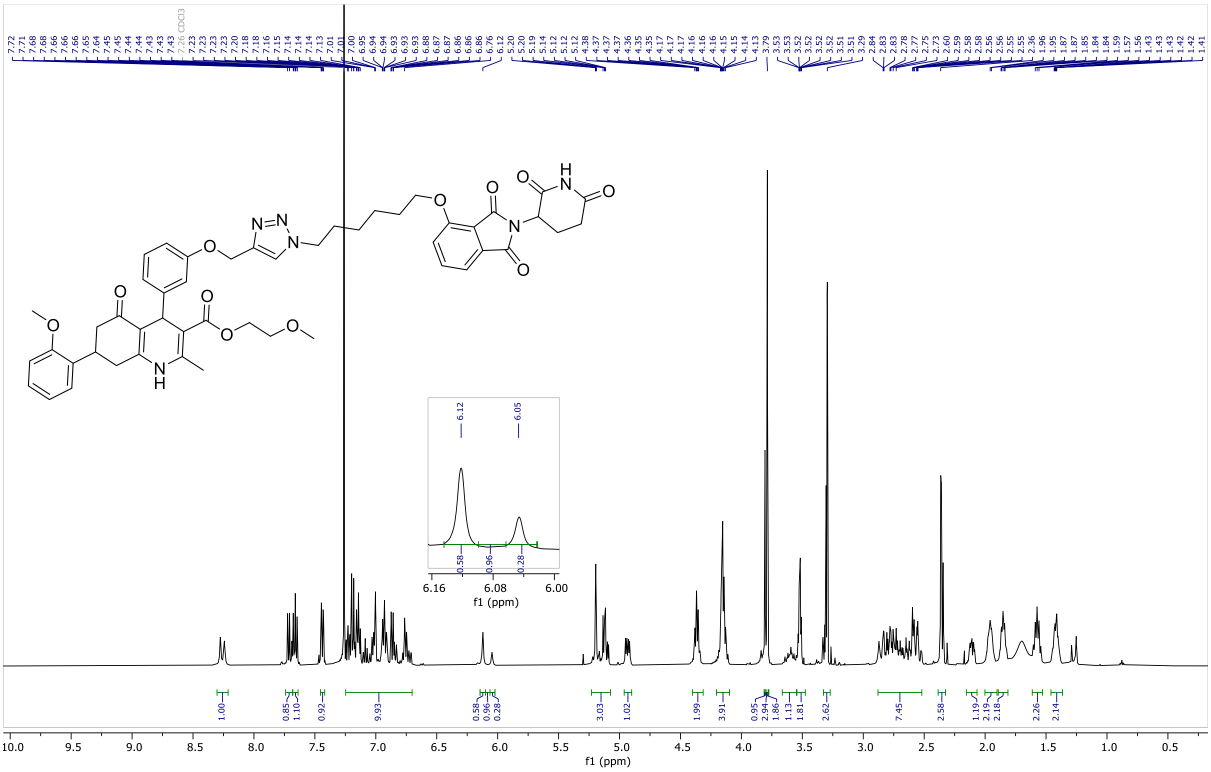

Supplementary Fig. 50. ^{1}H NMR (500 MHz, $CDCl_3$) of **HPP-9**. Diastereomeric pair ratio 2:1.

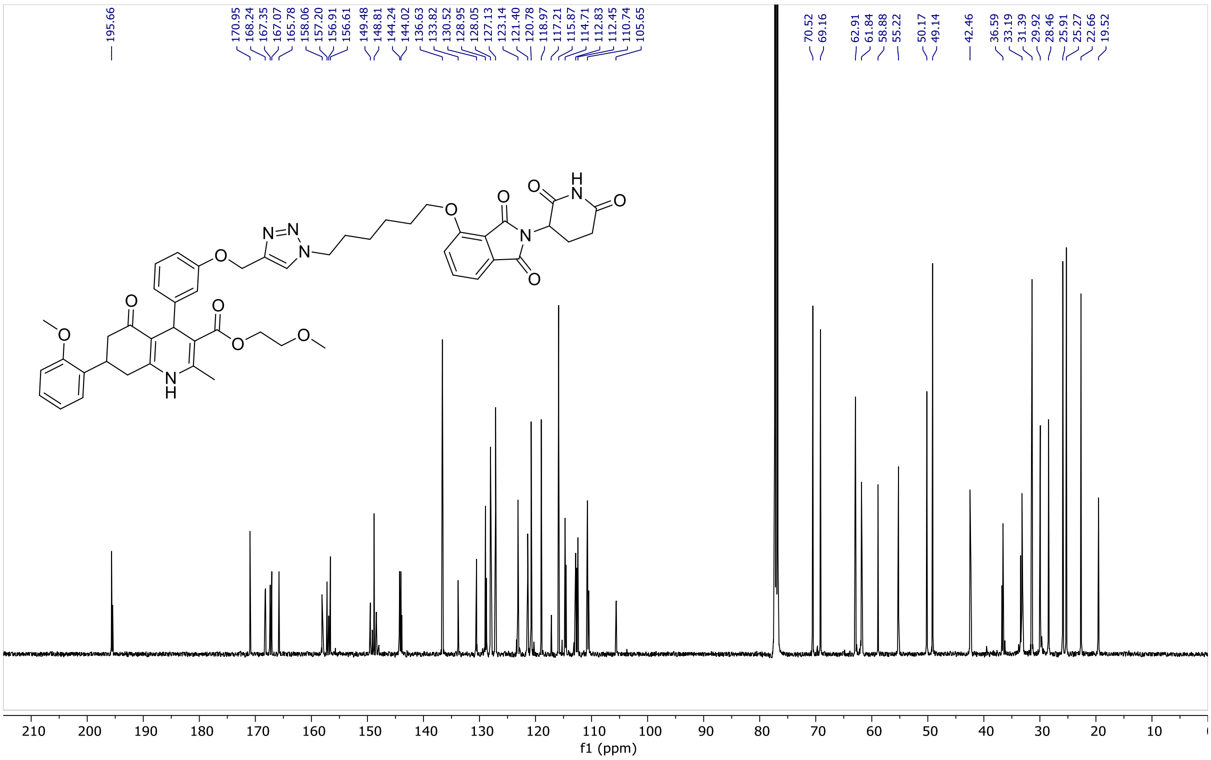

Supplementary Fig. 51. ^{13}C NMR (126 MHz, $CDCl_3$) of **HPP-9**.

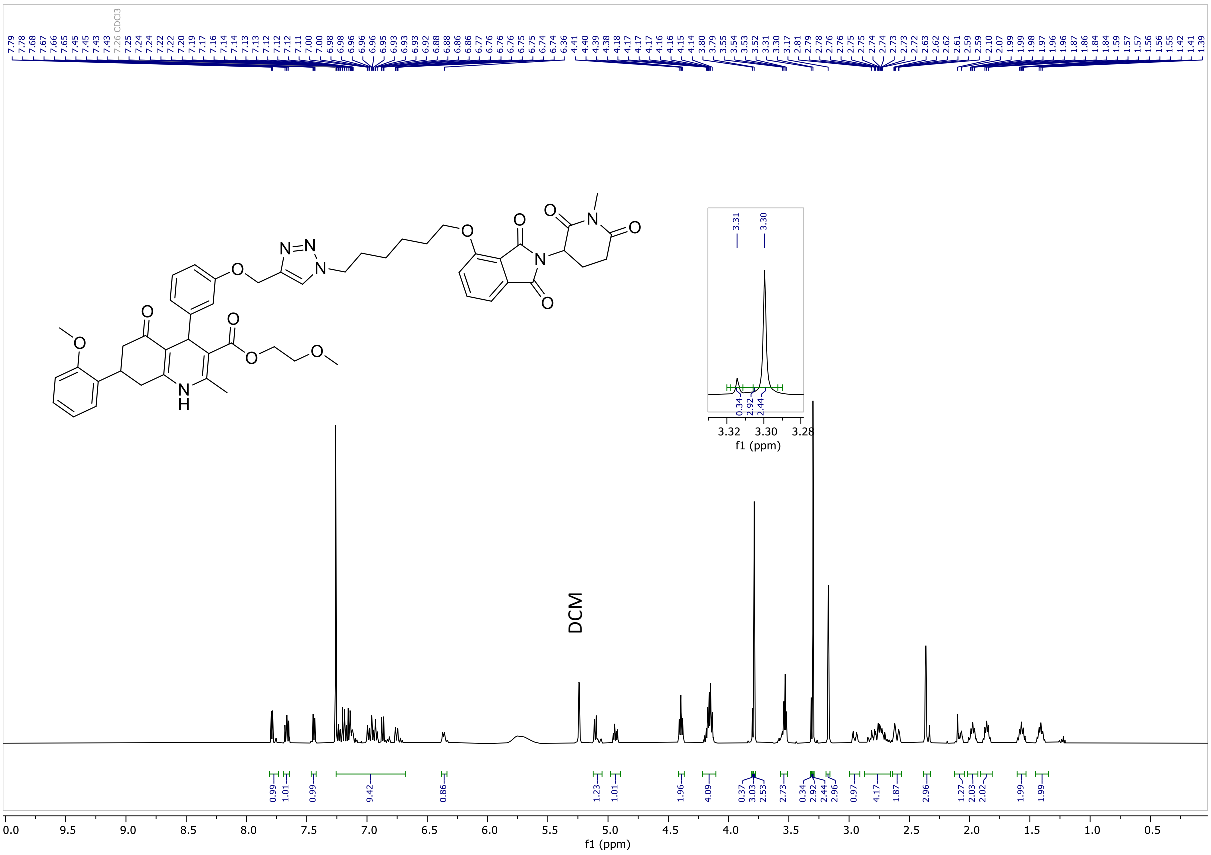

Supplementary Fig. 52. ^{1}H NMR (500 MHz, $CDCl_3$) of **HPP-9-inact**. Diastereomeric pair ratio 6.8:1.

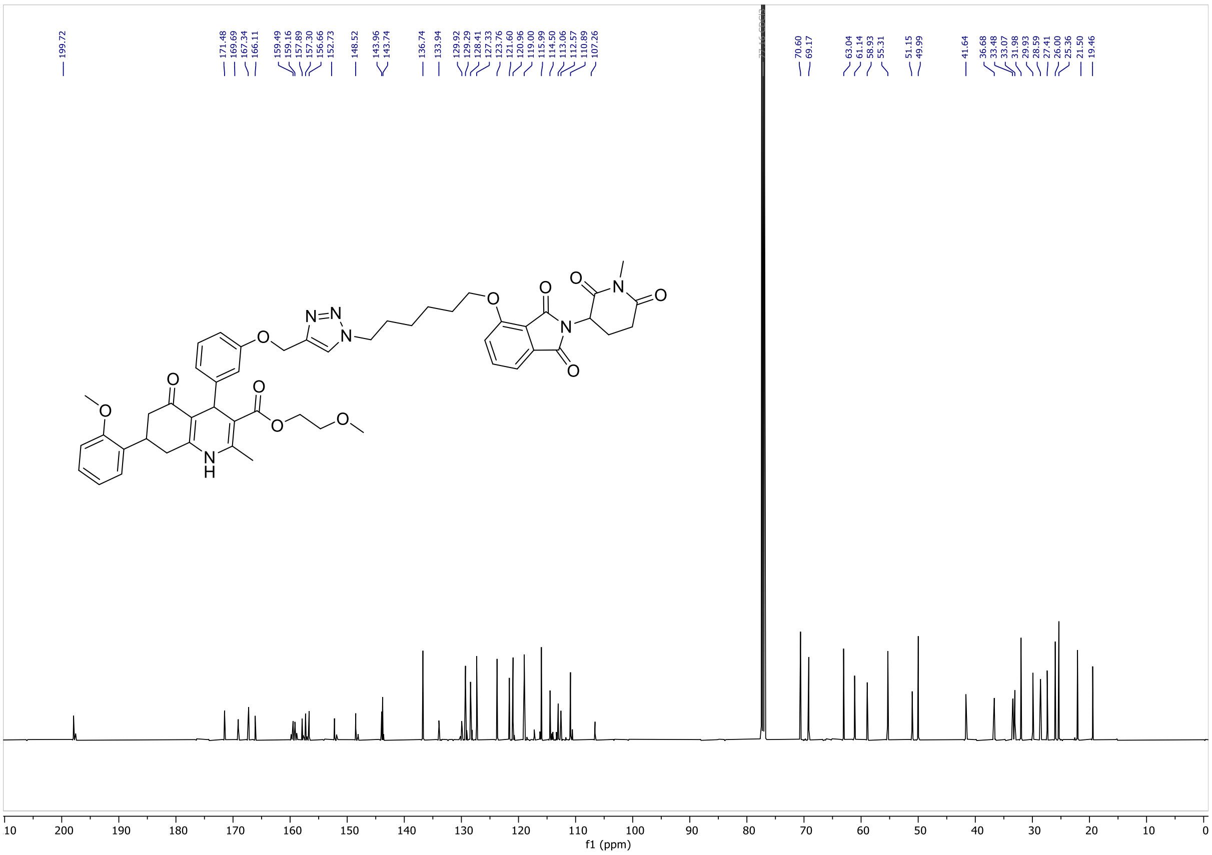

Supplementary Fig. 53. ^{13}C NMR (126 MHz, $CDCl_3$) of **HPP-9-inact**.

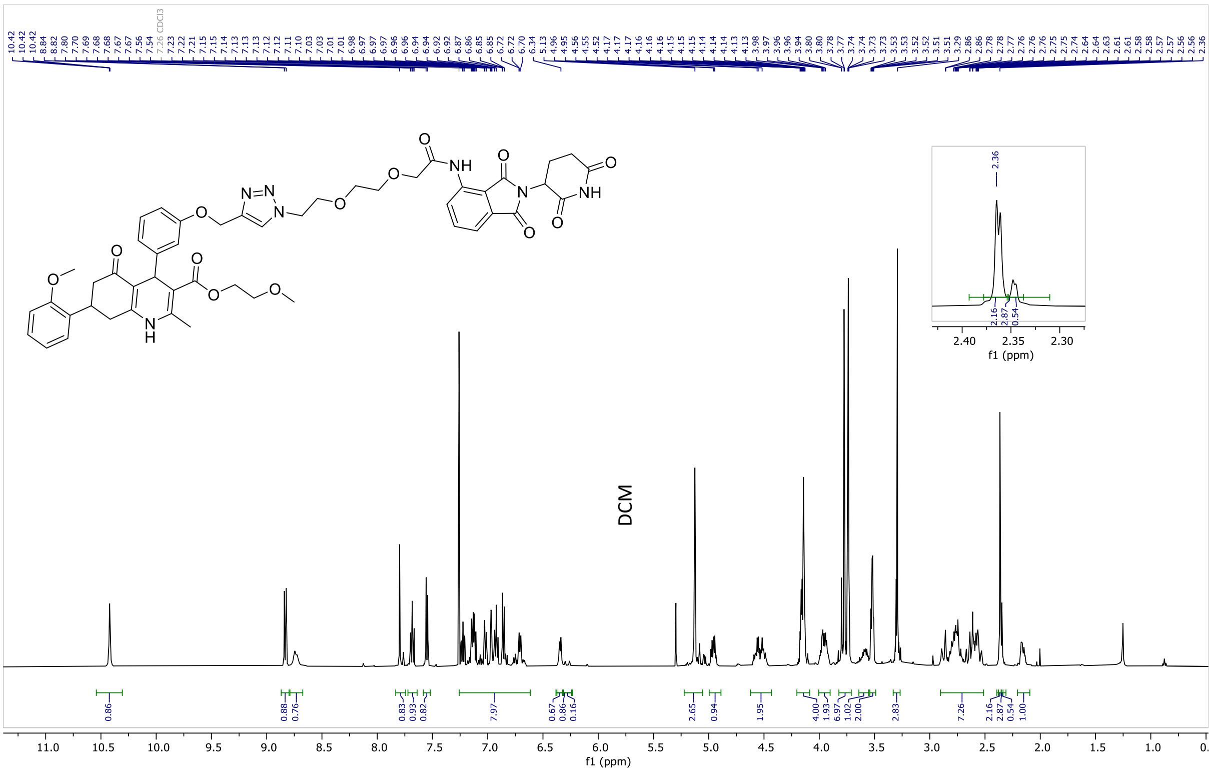

Supplementary Fig. 54. ^{1}H NMR (500 MHz, $CDCl_3$) of **HPP-10**. Diastereomeric pair ratio 4.2:1.

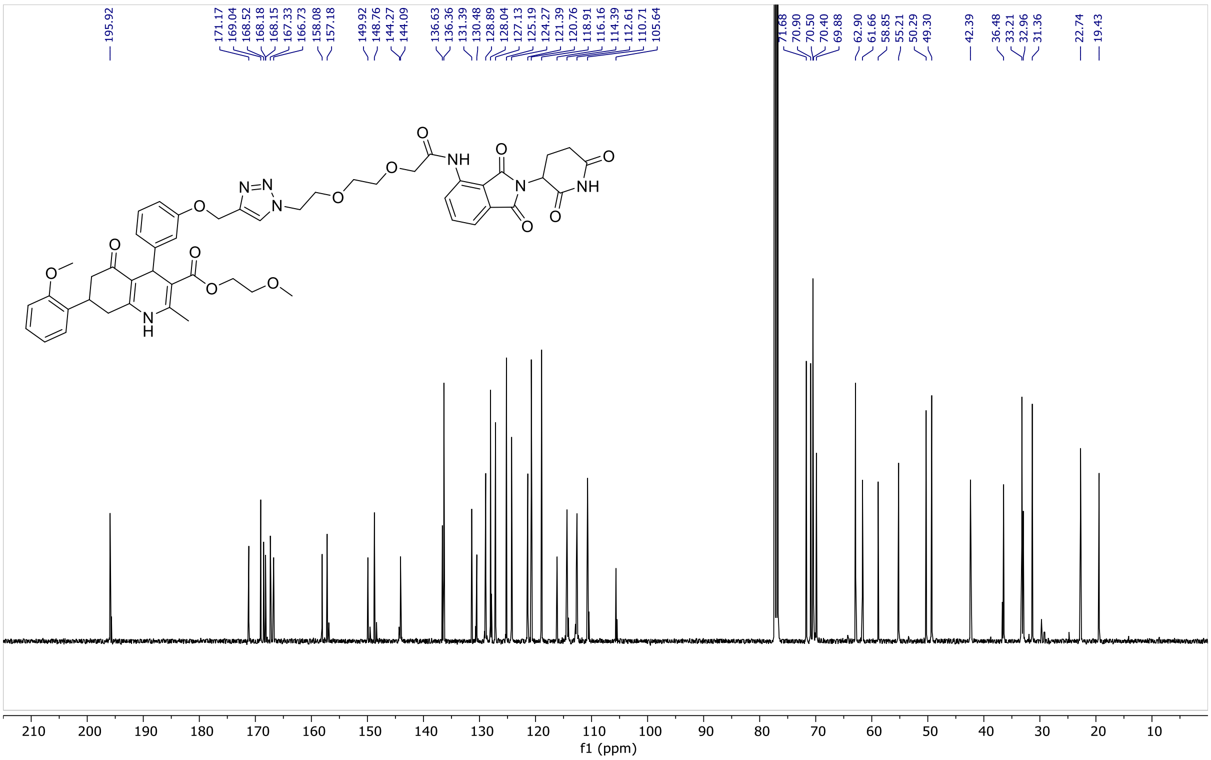

Supplementary Fig. 55. ^{13}C NMR (126 MHz, $CDCl_3$) of **HPP-10**.

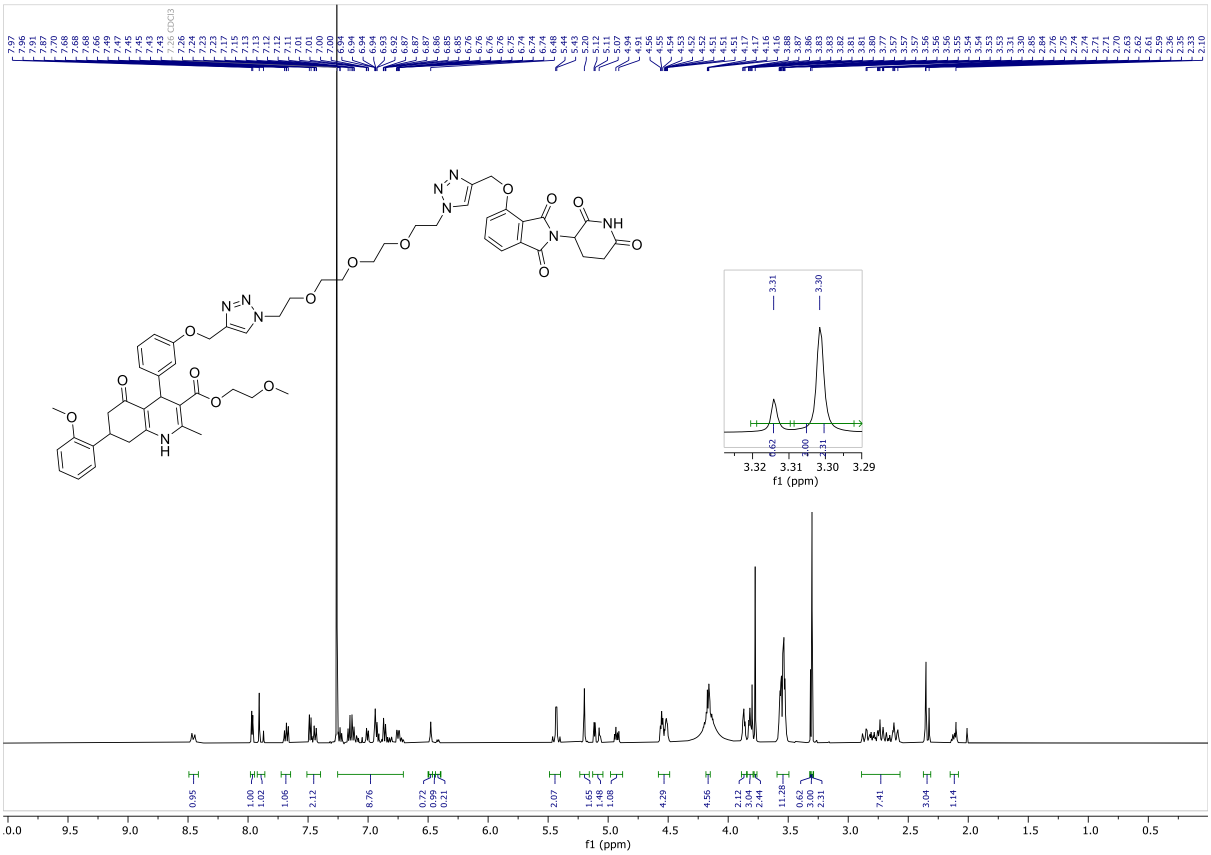

Supplementary Fig. 56. ^{1}H NMR (500 MHz, $CDCl_3$) of **HPP-11**. Diastereomeric pair ratio 3.6:1.

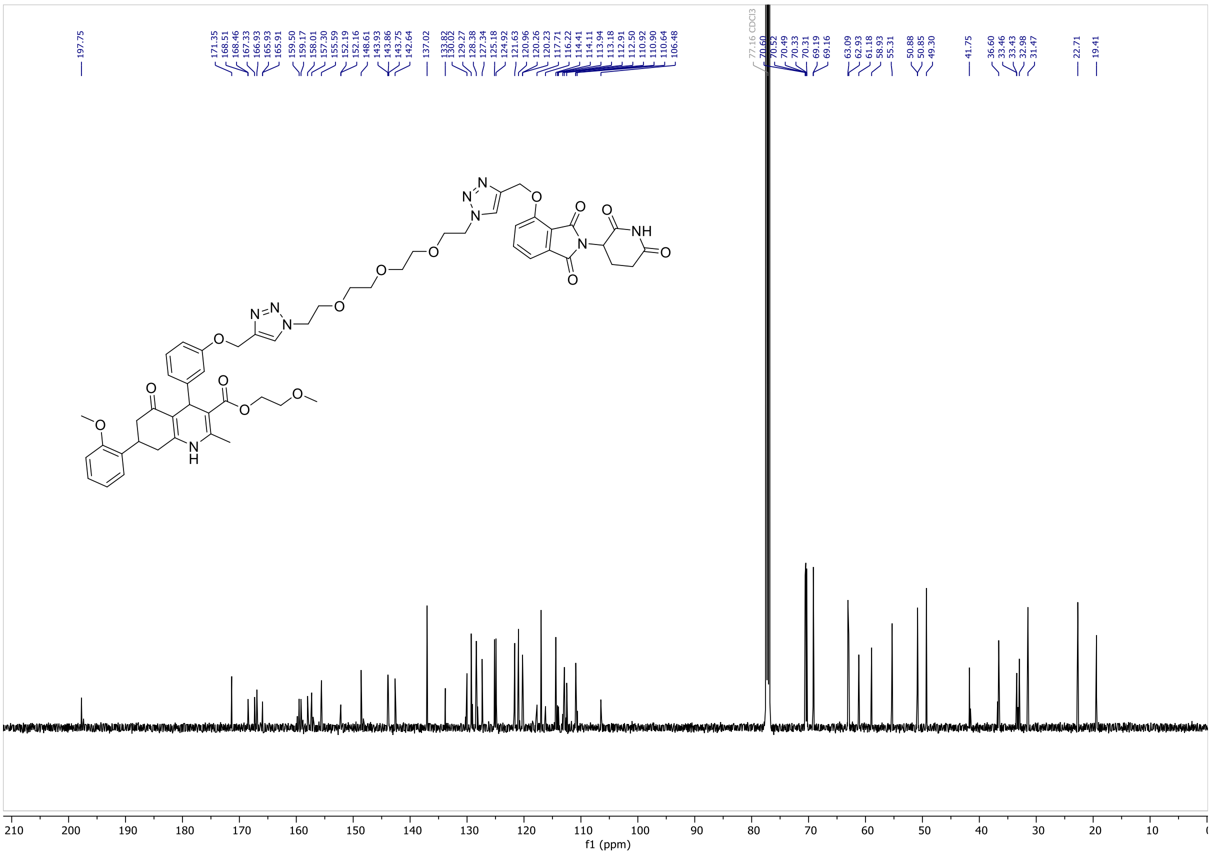

Supplementary Fig. 57. ^{13}C NMR (126 MHz, $CDCl_3$) of **HPP-11**.

4. LC-MS Spectra

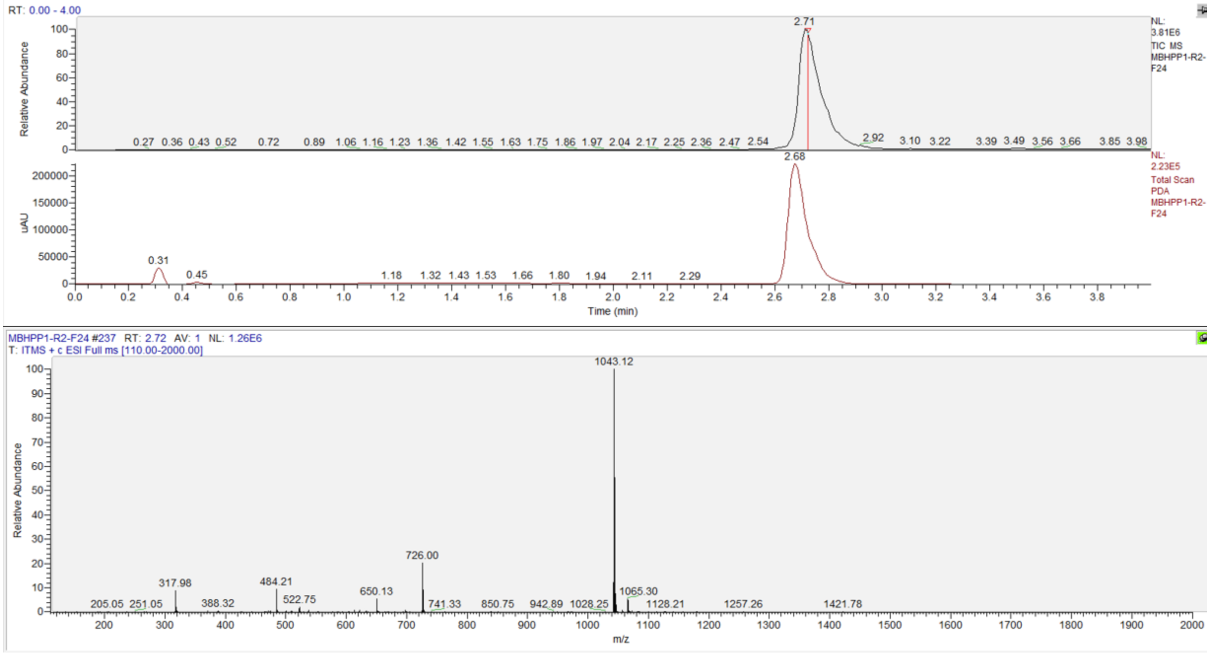

Supplementary Fig. 58. LC-MS of **HPP-1**.

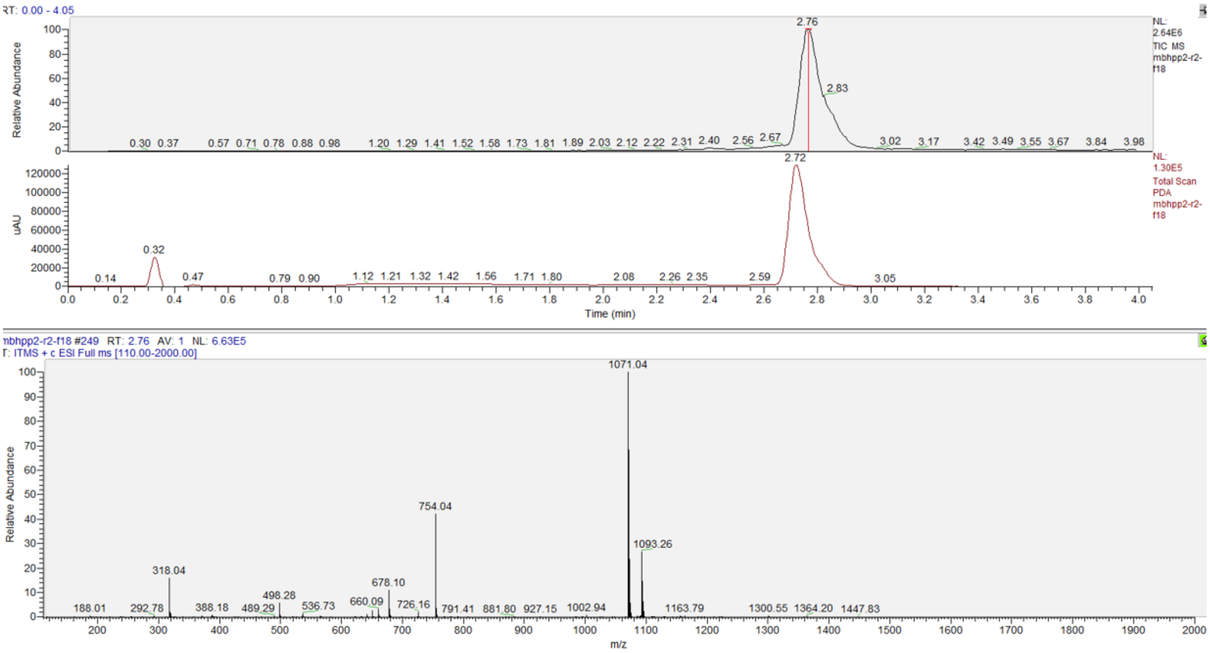

Supplementary Fig. 59. LC-MS of **HPP-2**.

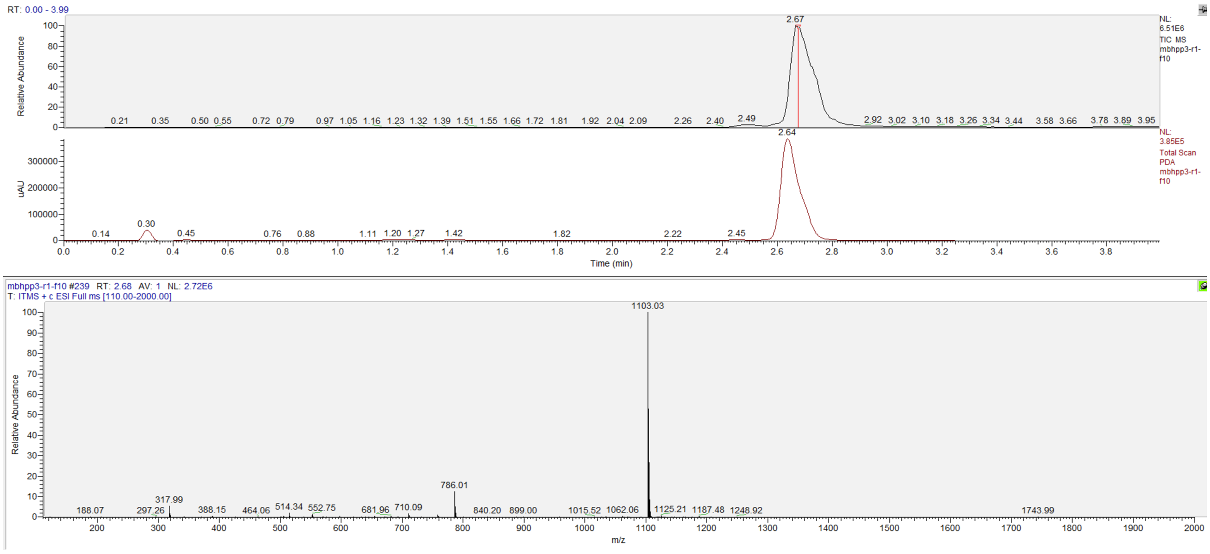

Supplementary Fig. 60. LC-MS of **HPP-3**.

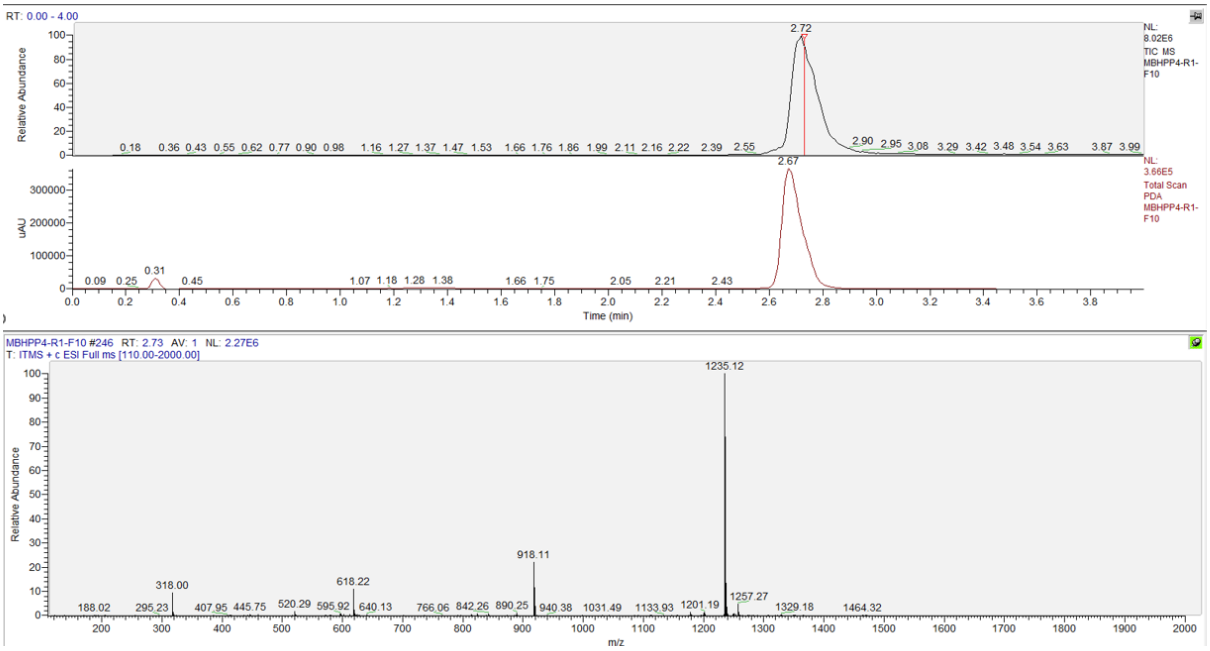

Supplementary Fig. 61. LC-MS of **HPP-4**.

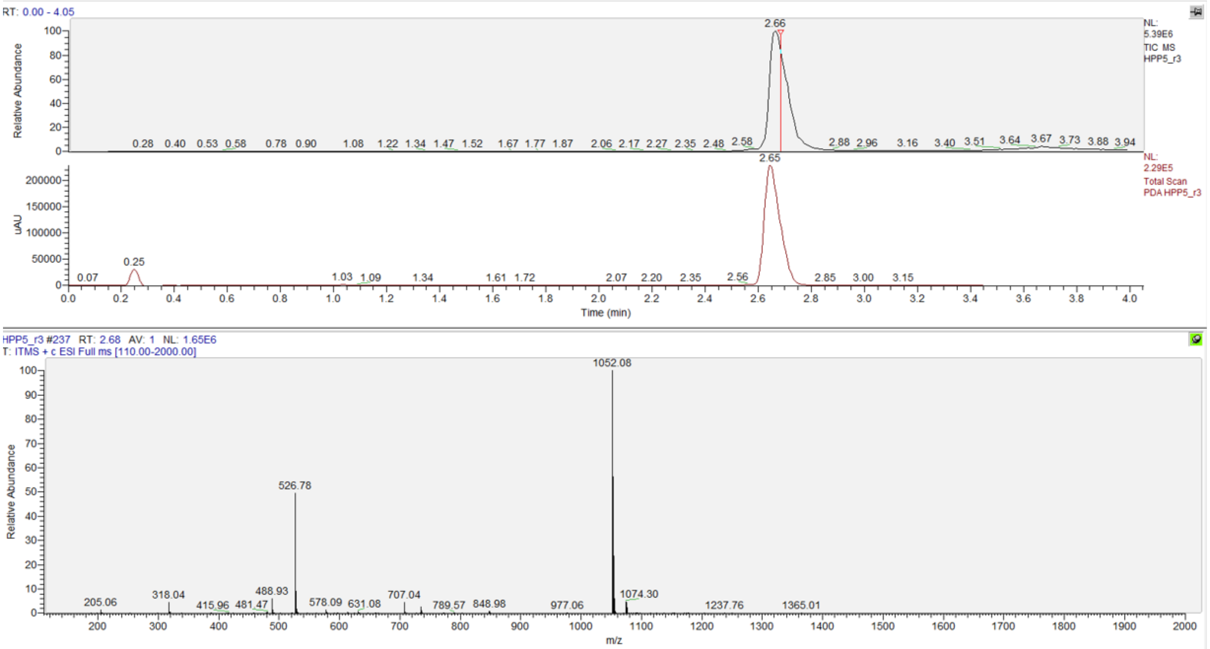

Supplementary Fig. 62. LC-MS of **HPP-5**.

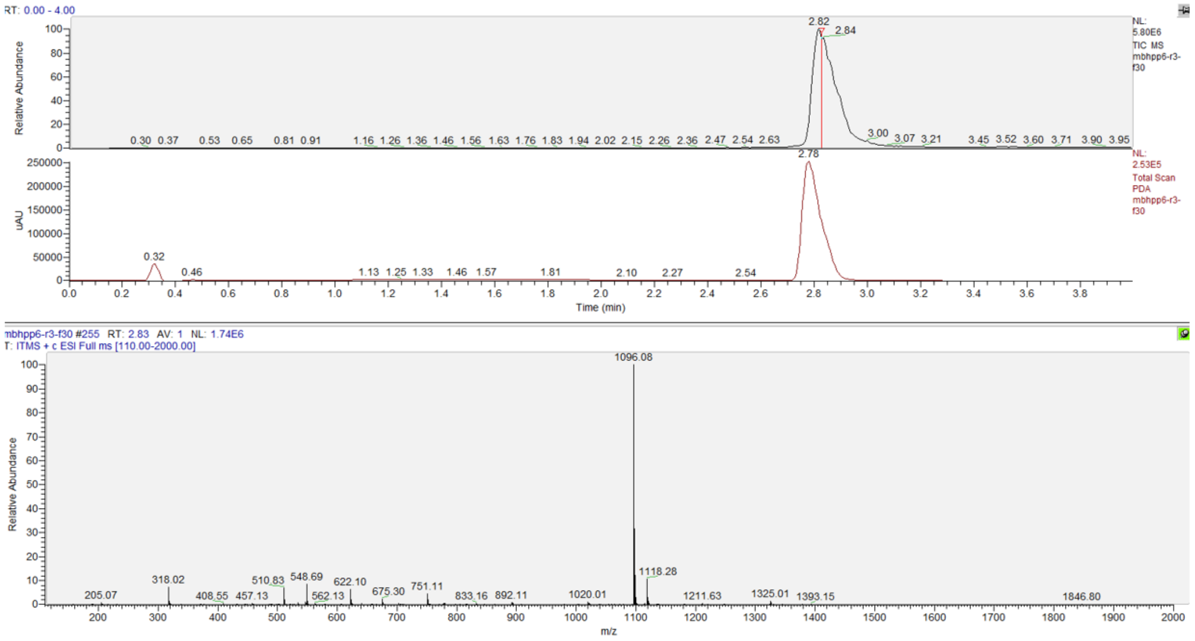

Supplementary Fig. 63. LC-MS of **HPP-6**.

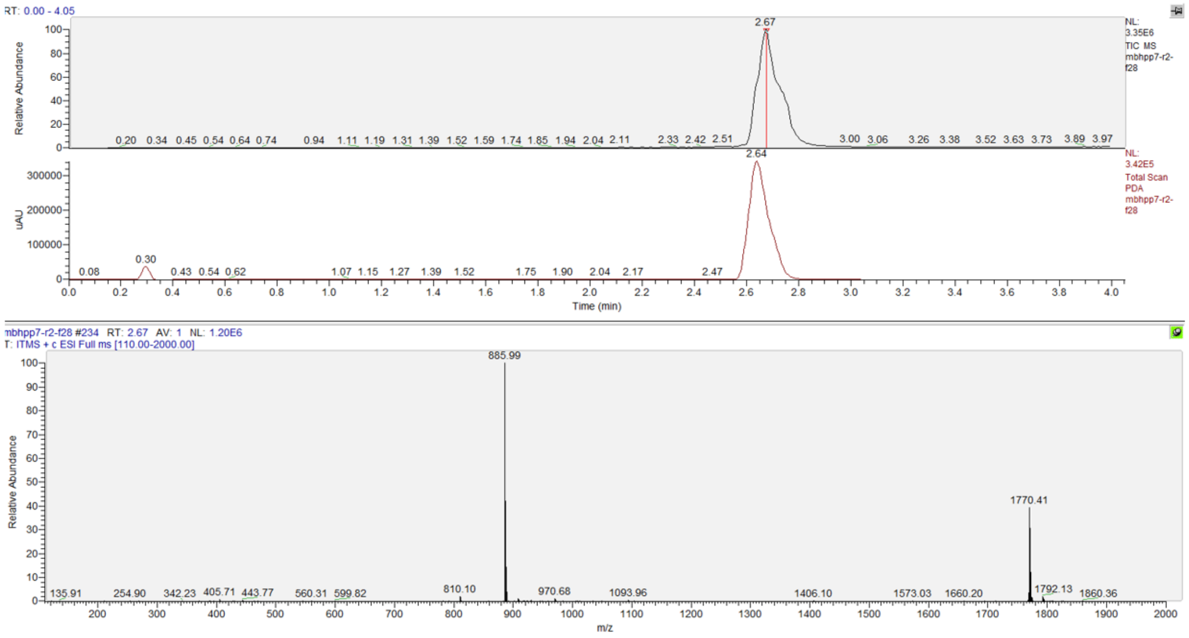

Supplementary Fig. 64. LC-MS of **HPP-7**.

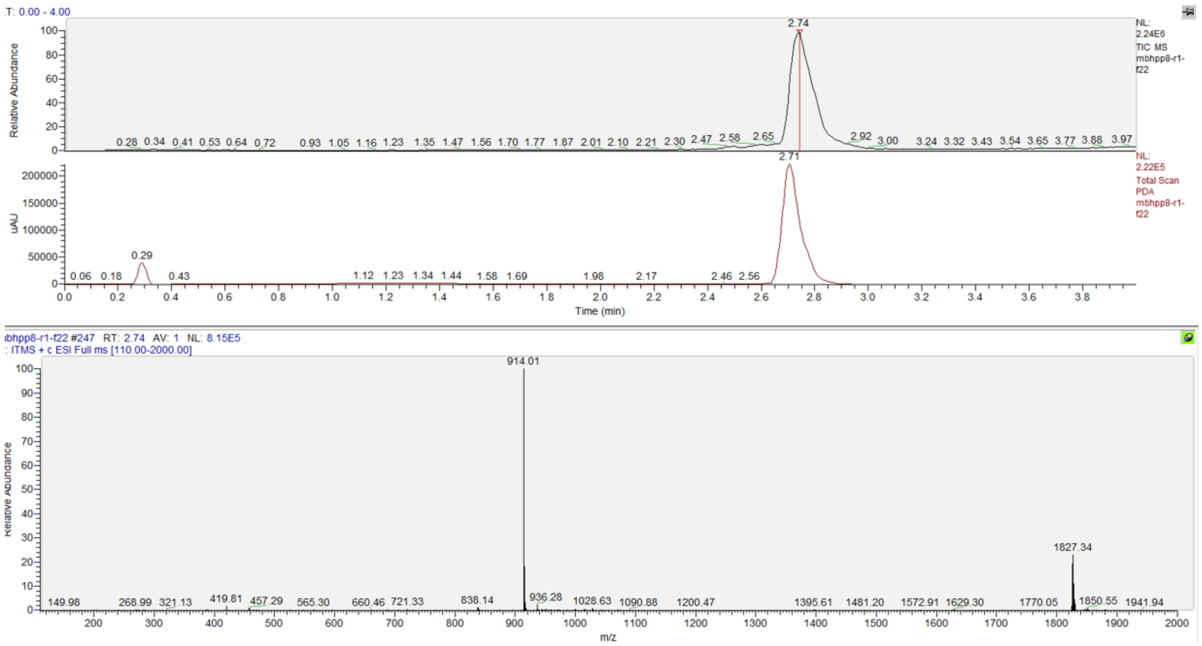

Supplementary Fig. 65. LC-MS of **HPP-8**.

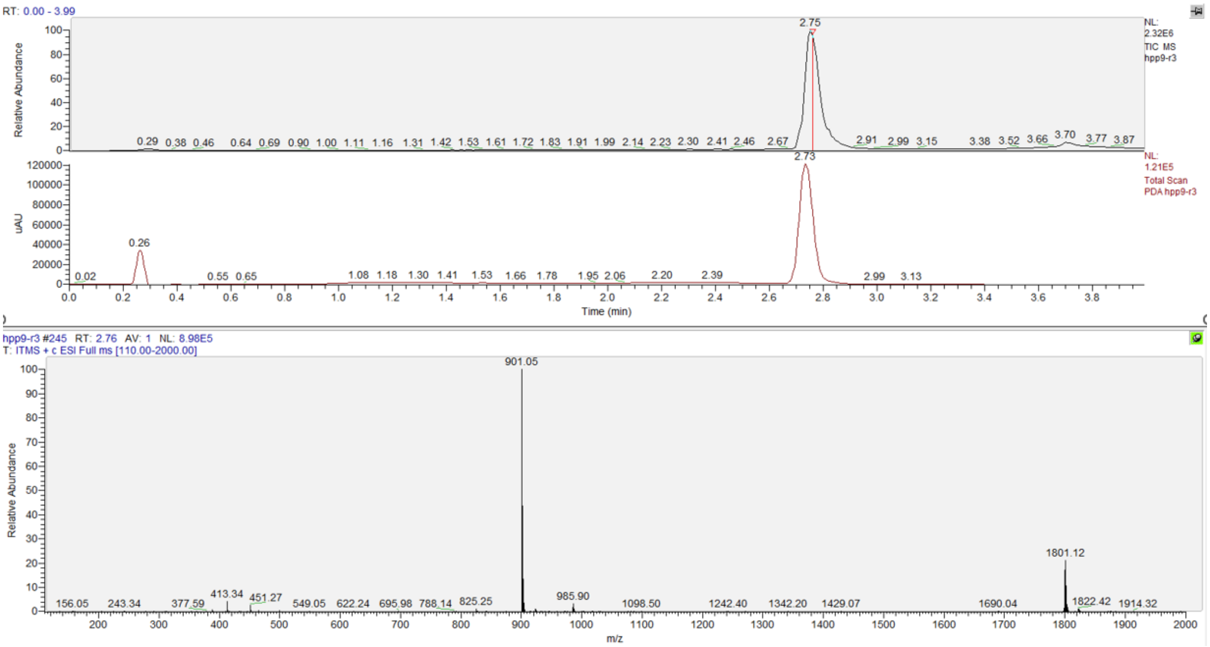

Supplementary Fig. 66. LC-MS of **HPP-9**.

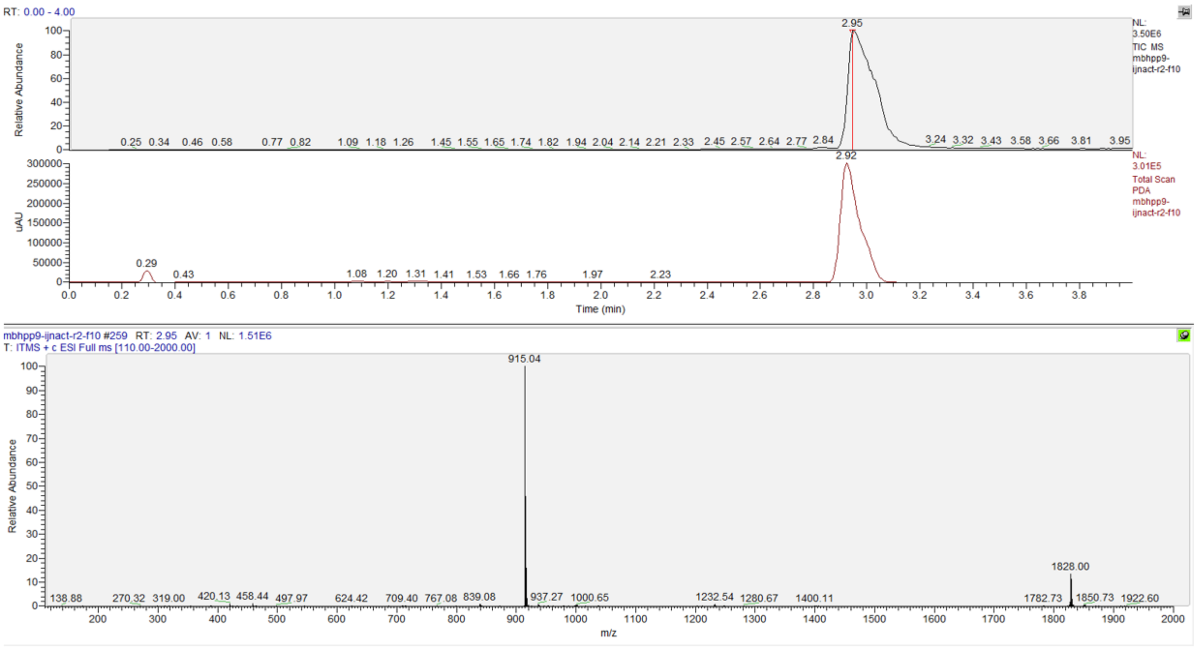

Supplementary Fig. 67. LC-MS of **HPP-9-inact**.

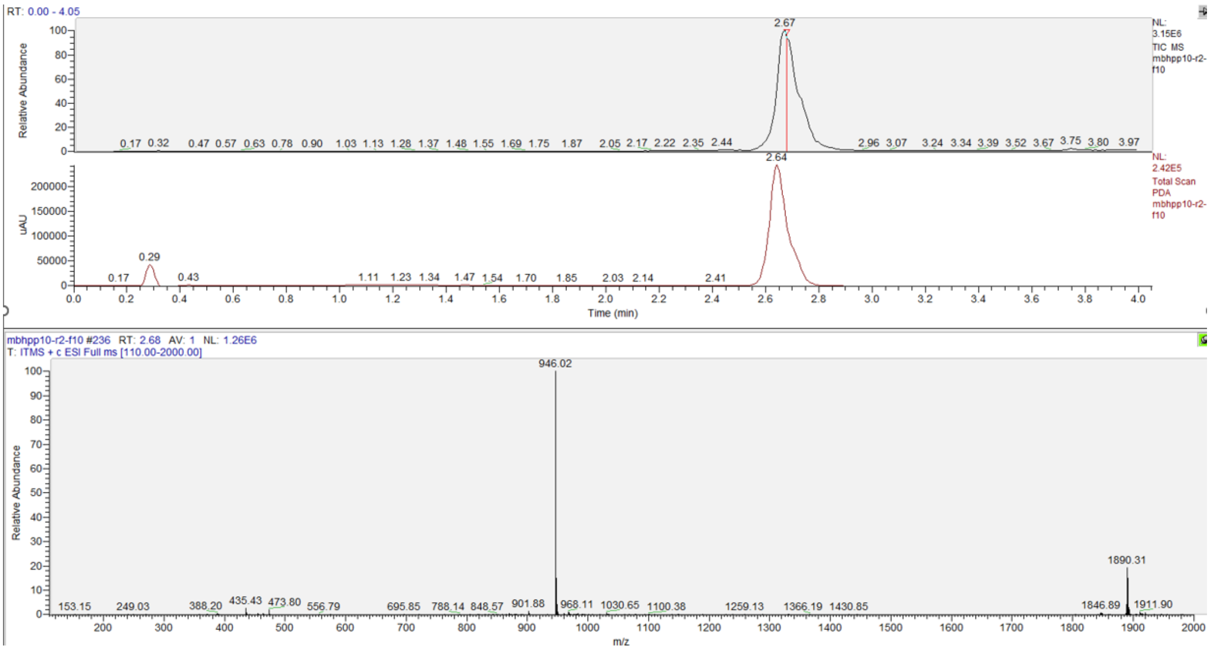

Supplementary Fig. 68. LC-MS of **HPP-10**.

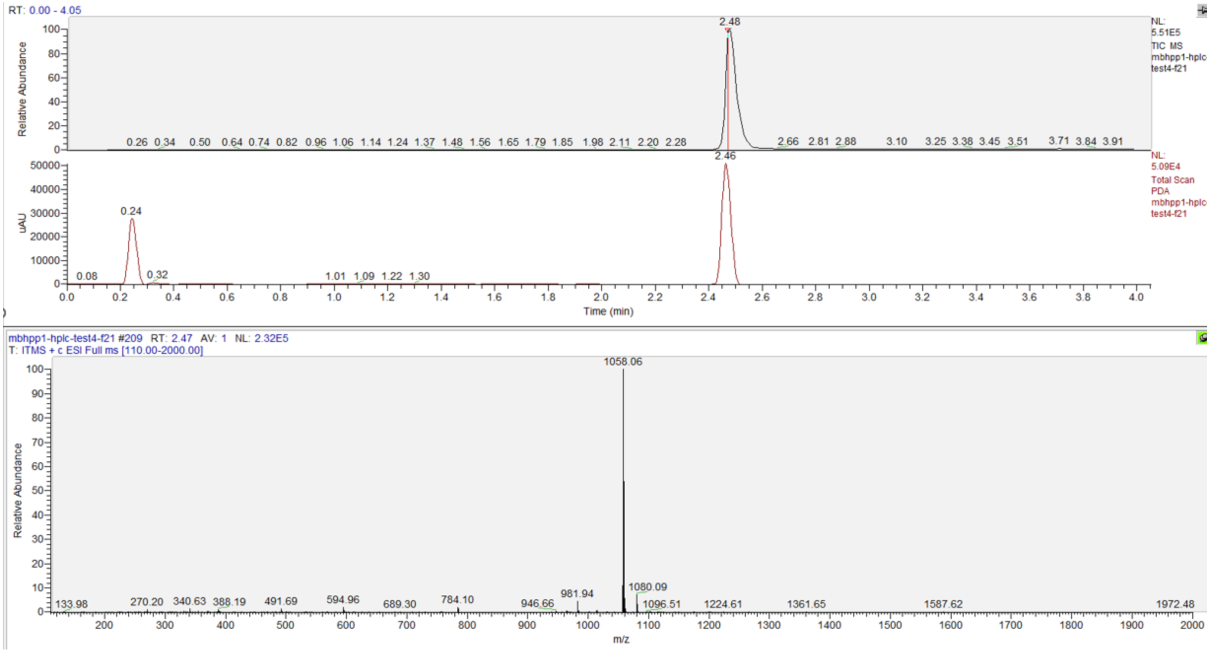

Supplementary Fig. 69. LC-MS of **HPP-11**.

5. Uncropped western blot membranes

Fig. 1D

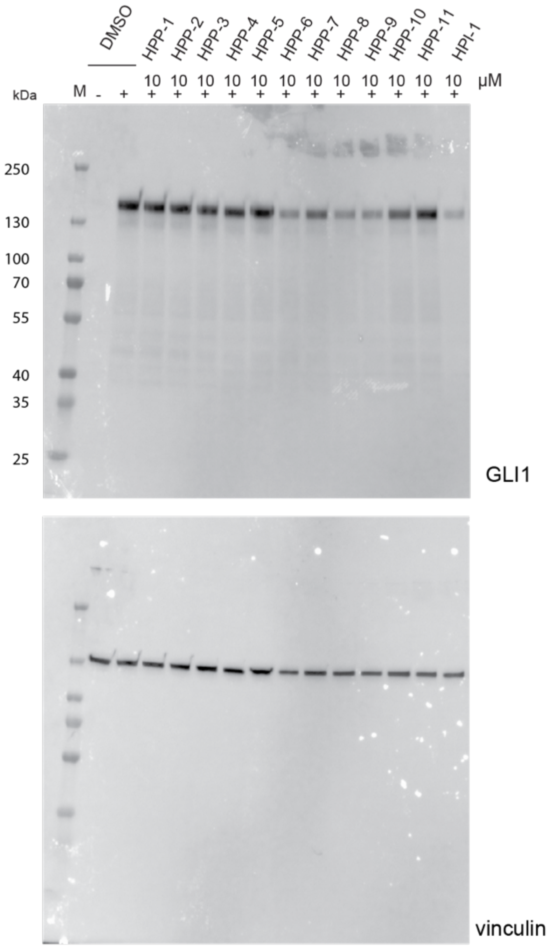

Fig. 2D

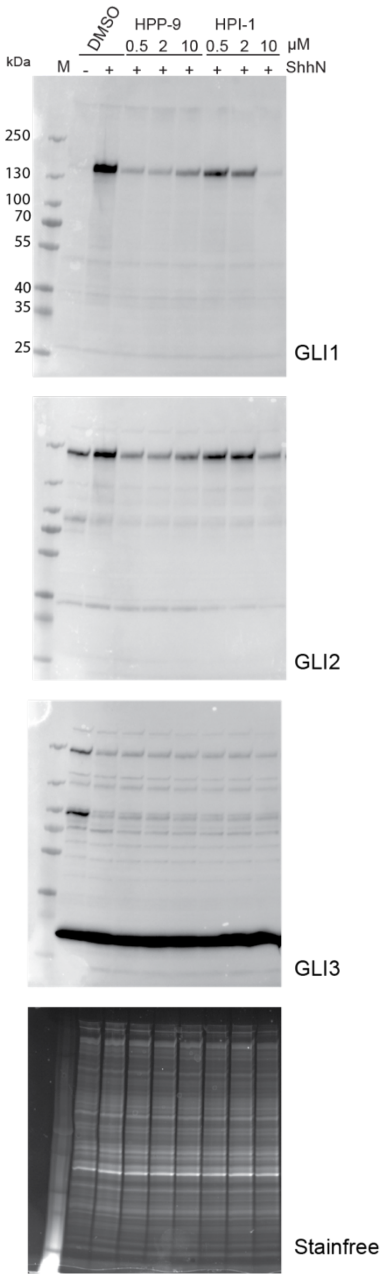

Fig. 3C

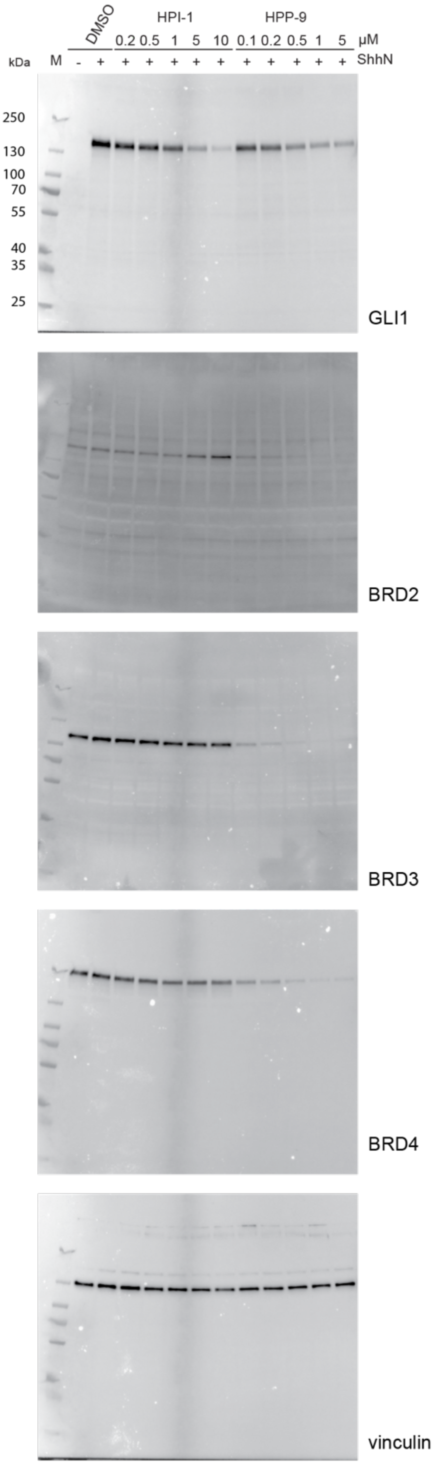
DMSO
HPI-1
HPP-9
0.2 0.5 1 5 10 0.1 0.2 0.5 1 5 µM
M - + + + + + + + + + + + ShhN
kDa
250
130
100
70
55
40
35
25
GLI1
BRD2
BRD3
BRD4
vinculin

Fig. 3D

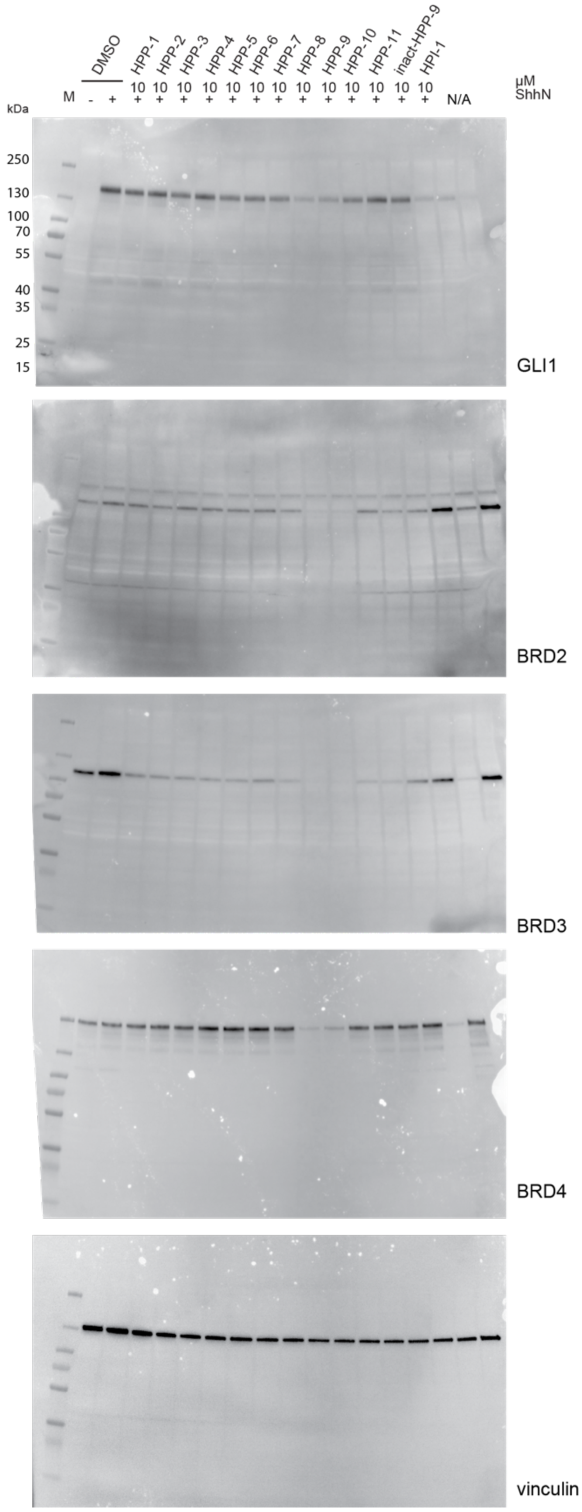
DMSO
HPP-1
HPP-2
HPP-3
HPP-4
HPP-5
HPP-6
HPP-7
HPP-8
HPP-9
HPP-10
HPP-11
inact-HPP-9
HPI-1
10 10 10 10 10 10 10 10 10 10 10 10 10 µM
M - + + + + + + + + + + + + + + N/A ShhN
kDa
250
130
100
70
55
40
35
25
15
GLI1
BRD2
BRD3
BRD4
vinculin

Fig.3F

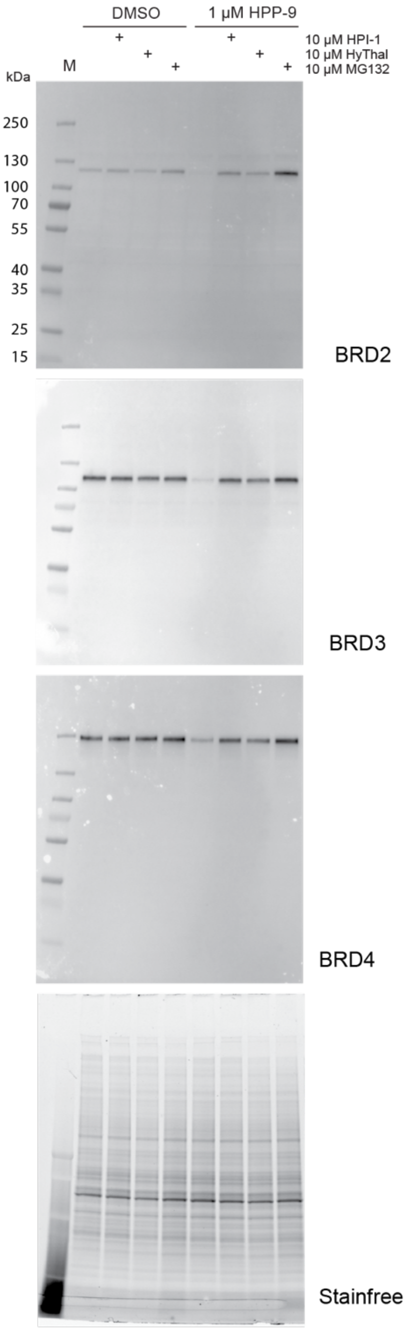
DMSO
1 µM HPP-9
10 µM HPI-1
10 µM HyThal
10 µM MG132
M
kDa
250
130
100
70
55
40
35
25
15
BRD2
BRD3
BRD4
Stainfree

Fig. 3G

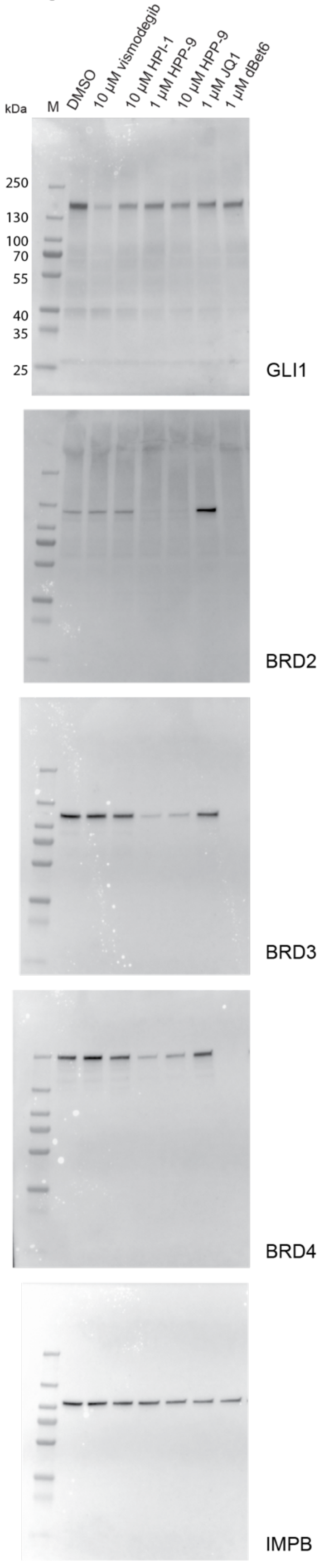
kDa
M
DMSO
10 µM vismodegib
10 µM HPI-1
1 µM HPP-9
10 µM HPP-9
1 µM JQ1
1 µM dBet6
250
130
100
70
55
40
35
25
GLI1
BRD2
BRD3
BRD4
IMPB

Fig. 5A & Supplementary Fig. 9

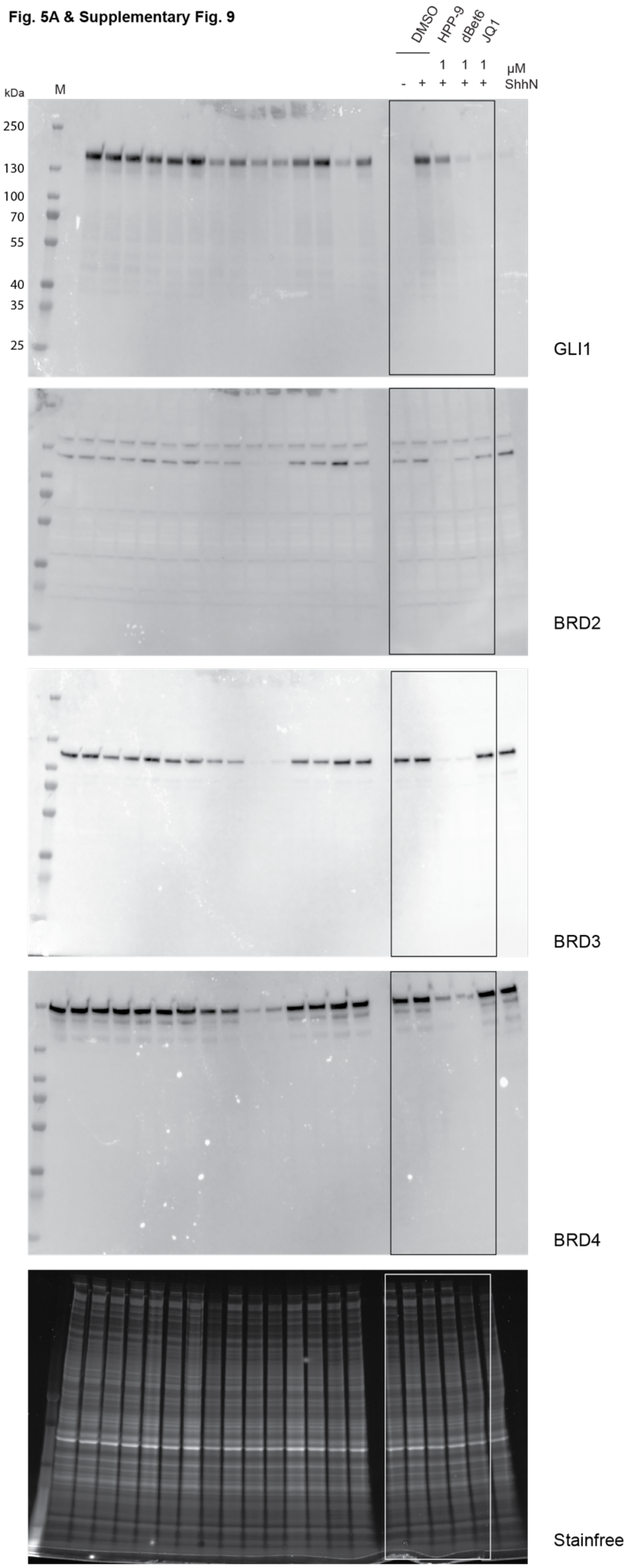

Fig. 6B

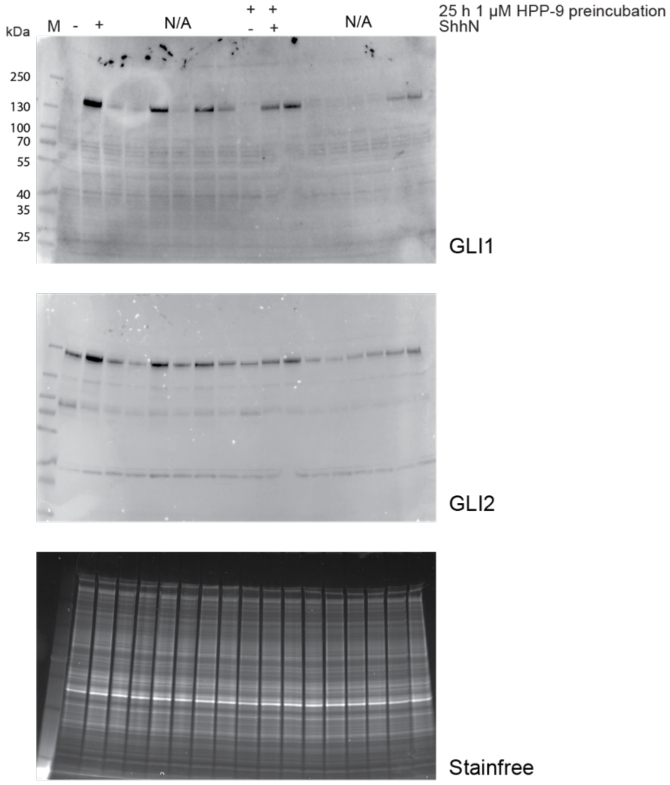
25 h 1 µM HPP-9 preincubation
ShhN
M
-
+
N/A
+
+
-
+
N/A
kDa
250
130
100
70
55
40
35
25
GLI1
GLI2
Stainfree

Supplementary Fig. 3

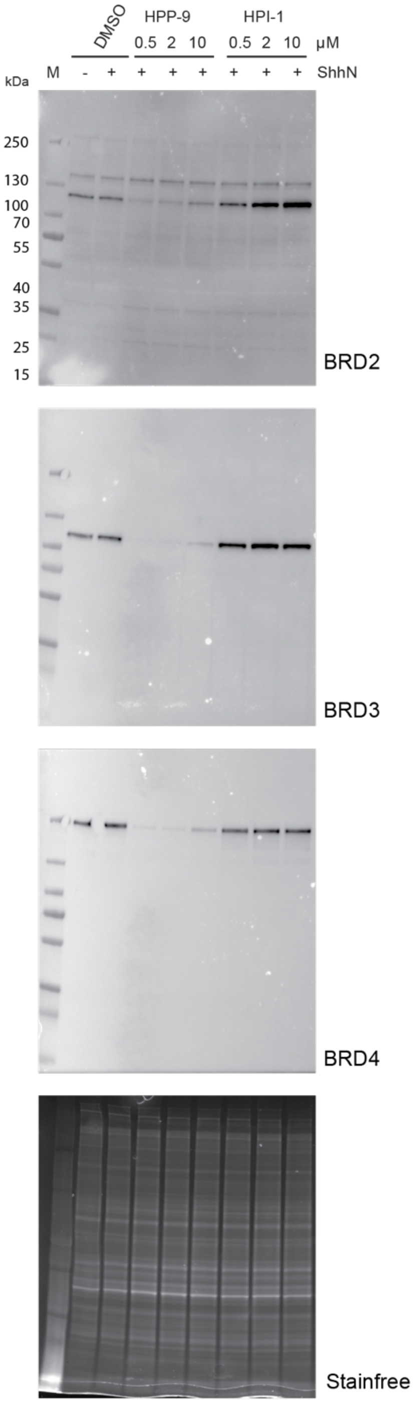

Supplementary Fig. 4

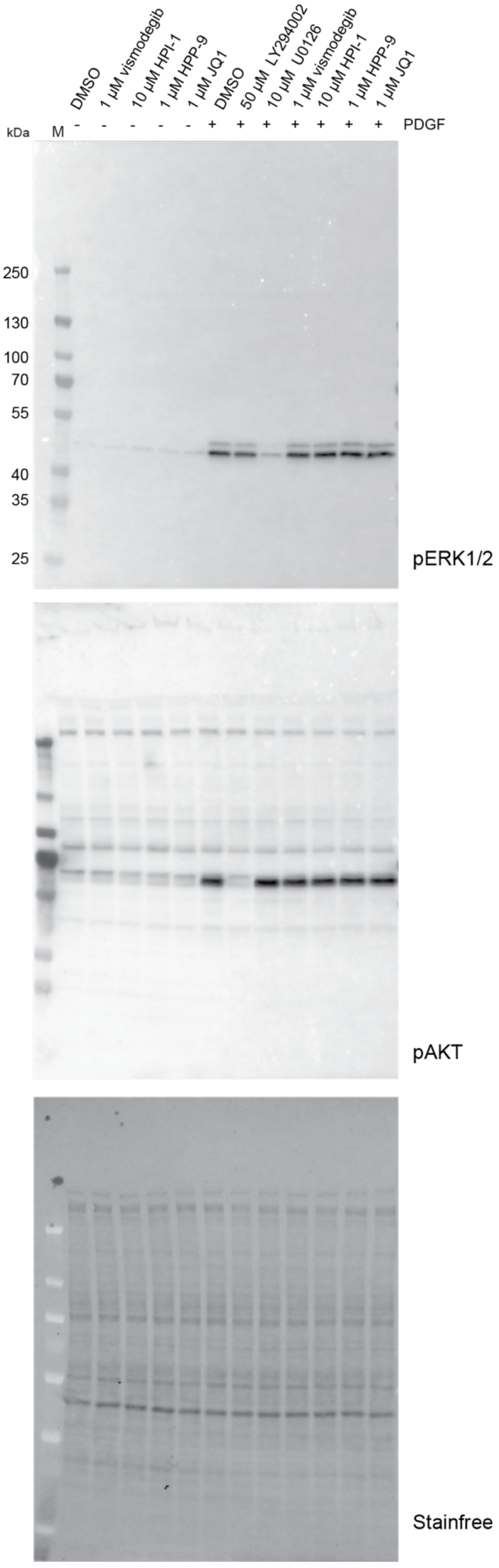

Supplementary Fig. 8

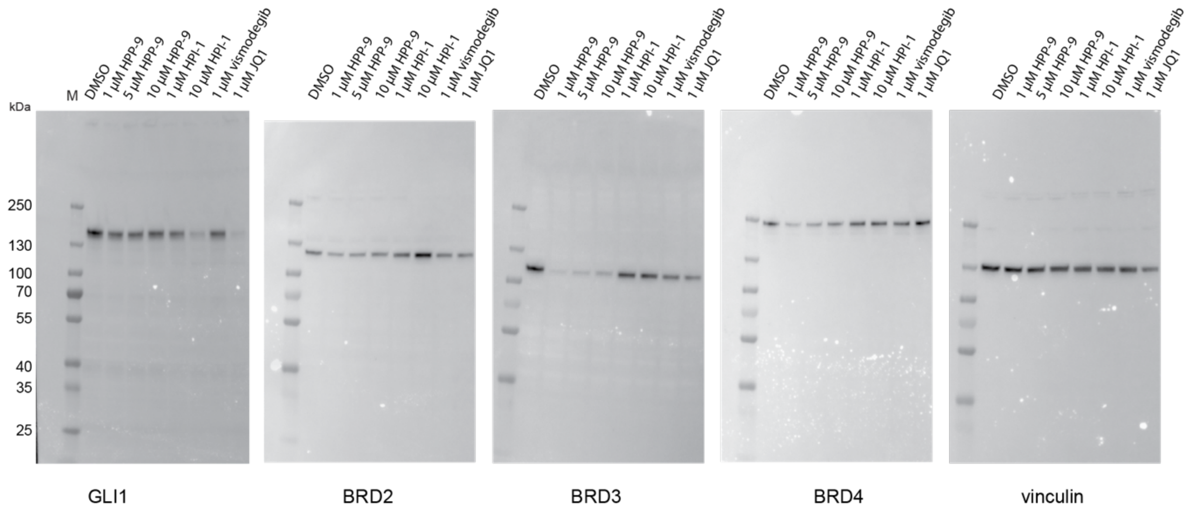

Supplementary Fig.13

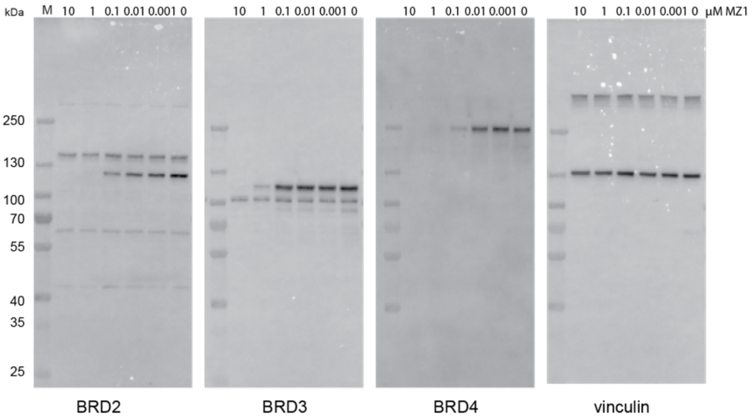

Supplementary Fig. 14

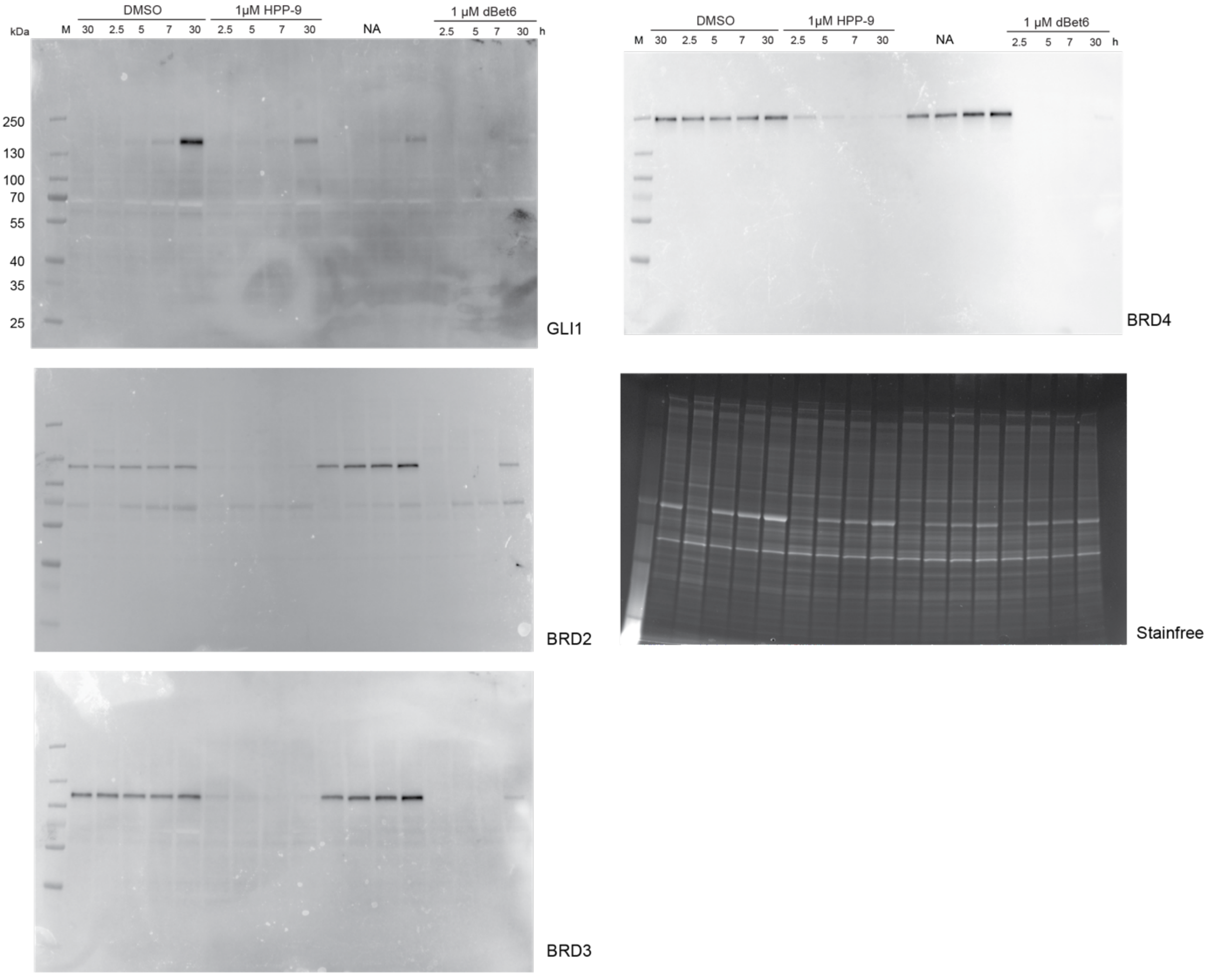

6. Supplementary References

[1] Chen, J. K., Hyman, J. M., Ocasio, C. A. Hedgehog Pathway Antagonists Methods of Use. US8119640B2. 21-23 (2009).

[2] Hyman, J. M. *et al.* Small-molecule inhibitors reveal multiple strategies for Hedgehog pathway blockade. *Proc. Natl. Acad. Sci. U. S. A.* **106,** 14132–14137 (2009).

[3] Crew, A. P. *et al.* Identification and Characterization of Von Hippel-Lindau-Recruiting Proteolysis Targeting Chimeras (PROTACs) of TANK-Binding Kinase 1. *J. Med. Chem.* **61,** 583–598 (2018).

[4] Madak, J. T., Cuthbertson, C. R., Chen, W., Showalter, H. D. & Neamati, N. Design, Synthesis, and Characterization of Brequinar Conjugates as Probes to Study DHODH Inhibition. *Chemistry.* **23,** 13875-13878 (2017).

[5] Liu, K. & Zhang, S. Design and characterization of 3-Azidothalidomide as a selective hydrogen sulfide probe. *Tetrahedron Lett.* **55,** 5566–5569 (2014).

[6] Zhou, L. *et al.* Design and synthesis of α-naphthoflavone chimera derivatives able to eliminate cytochrome P450 (CYP)1B1-mediated drug resistance via targeted CYP1B1 degradation. *Eur. J. Med. Chem.* **189,** 112028 (2020).

[7] López-Andarias, J. *et al.* Cell-Penetrating Streptavidin: A General Tool for Bifunctional Delivery with Spatiotemporal Control, Mediated by Transport Systems Such as Adaptive Benzopolysulfane Networks. *J. Am. Chem. Soc.* **142,** 4784–4792 (2020).

[8] Bonger, K. M. *et al.* Synthesis and evaluation of homo-bivalent GnRHR ligands. *Bioorg. Med. Chem.* **15,** 4841–4856 (2007).

[9] Ranjan N. *et al.* Selective Inhibition of Escherichia coli RNA and DNA Topoisomerase I by Hoechst 33258 Derived Mono- and Bisbenzimidazoles. *J. Med. Chem.* **60**, 4904-4922 (2017).

[10] Li, W. *et al.* Phthalimide conjugations for the degradation of oncogenic PI3K. *Eur. J. Med. Chem.* **151,** 237–247 (2018).
