# Supplementary material for: Targeted protein degradation reveals BET bromodomains as the cellular target of Hedgehog pathway inhibitor-1
Source: Nat Commun. 2023 Jul 1;14:3893. doi: 10.1038/s41467-023-39657-1 (PMC10314895; doi:10.1038/s41467-023-39657-1)
Supplement: Supplementary file 2 — Description of Additional Supplementary Files [file 41467_2023_39657_MOESM2_ESM.docx]

File name: Supplementary Data 1

Description: Normalized protein abundances in each sample. Protein quantification was performed using Spectronaut as described in the Methods section.

File name: Supplementary Data 2

Description: Two-group comparison of protein abundances. Fold change and differential abundance testing were described in the Methods section. P-values were calculated from unpaired t-test and Q-values were the multiple testing corrected p-values.

File name: Supplementary Data 3

Description: RNAseq data, including raw and normalized counts as well as DESeq2 results. The default settings of DESeq2 were used which calculates p values using the Wald test and then the adjusted p values that correct for multiple testing using the Benjamin-Hochberg correction.
