# Supplementary material for: Targeted protein degradation reveals BET bromodomains as the cellular target of Hedgehog pathway inhibitor-1
Source: Nat Commun. 2023 Jul 1;14:3893. doi: 10.1038/s41467-023-39657-1 (PMC10314895; doi:10.1038/s41467-023-39657-1)
Supplement: Supplementary file 6 — Reporting Summary [file 41467_2023_39657_MOESM6_ESM.pdf]

nature portfolio

Corresponding author(s): Sascha Hoogendoorn

Last updated by author(s): 2023/06/04

Reporting Summary

Nature Portfolio wishes to improve the reproducibility of the work that we publish. This form provides structure for consistency and transparency in reporting. For further information on Nature Portfolio policies, see our Editorial Policies and the Editorial Policy Checklist.

Statistics

For all statistical analyses, confirm that the following items are present in the figure legend, table legend, main text, or Methods section.

n/a	Confirmed	
☐	☒	The exact sample size (*n*) for each experimental group/condition, given as a discrete number and unit of measurement
☐	☒	A statement on whether measurements were taken from distinct samples or whether the same sample was measured repeatedly
☐	☒	The statistical test(s) used AND whether they are one- or two-sided<br>*Only common tests should be described solely by name; describe more complex techniques in the Methods section.*
☒	☐	A description of all covariates tested
☐	☒	A description of any assumptions or corrections, such as tests of normality and adjustment for multiple comparisons
☐	☒	A full description of the statistical parameters including central tendency (e.g. means) or other basic estimates (e.g. regression coefficient) AND variation (e.g. standard deviation) or associated estimates of uncertainty (e.g. confidence intervals)
☐	☒	For null hypothesis testing, the test statistic (e.g. *F*, *t*, *r*) with confidence intervals, effect sizes, degrees of freedom and *P* value noted<br>*Give P values as exact values whenever suitable.*
☒	☐	For Bayesian analysis, information on the choice of priors and Markov chain Monte Carlo settings
☒	☐	For hierarchical and complex designs, identification of the appropriate level for tests and full reporting of outcomes
☐	☒	Estimates of effect sizes (e.g. Cohen's *d*, Pearson's *r*), indicating how they were calculated

Our web collection on statistics for biologists contains articles on many of the points above.

Software and code

Policy information about availability of computer code

Data collection	no software was used to collect the data
Data analysis	Images were analysed using MetaXpress software (version 6.5.3.247, Molecular Devices, LLC). Custom Matlab (R2020b, Mathworks) scripts for cilia image analysis are those used in PMID: 29459677 and are available in Zenodo with the identifier 10.5281/zenodo.7041023. Proteomics data was analyzed using SpectroMine software (Biognosys, Switzerland). Docking studies were performed in Maestro release 2021-1 (Schrödinger, LLC, New York, NY, 2021) and visualized using ChimeraX (PMID: 32881101). Ternary complexes were generated using Rosetta software (PMID: 32483333). OMEGA 4.1.0.0 (OpenEye Scientific Software, Santa Fe, NM, USA) was used to generated low-energy conformers. RNAseq reads were processed using https://github.com/NLykoskoufis/BraunLabPipeline. Data was plotted and analyzed using GraphPad Prism 9 (GraphPad software LCC). Western blots band intensities were determined using Fiji Image J2 (version 2.3.0/1.53q, National Institute of Health)

For manuscripts utilizing custom algorithms or software that are central to the research but not yet described in published literature, software must be made available to editors and reviewers. We strongly encourage code deposition in a community repository (e.g. GitHub). See the Nature Portfolio guidelines for submitting code & software for further information.

Data

Policy information about availability of data

All manuscripts must include a data availability statement. This statement should provide the following information, where applicable:

- Accession codes, unique identifiers, or web links for publicly available datasets
- A description of any restrictions on data availability
- For clinical datasets or third party data, please ensure that the statement adheres to our policy

The raw experimental data that support the findings of this study are available in Zenodo with the identifier 10.5281/zenodo.7041023. The mass spectrometry proteomics data have been deposited to the ProteomeXchange Consortium via the PRIDE [1] partner repository with the dataset identifier PXD036539 and PXD040859. The sequencing data generated and analyzed in this study are available in the Gene Expression Omnibus repository with accession GSE228015 (https://www.ncbi.nlm.nih.gov/geo/query/acc.cgi?acc=GSE228015). Details on chemical synthesis, analytical spectra and uncropped western blots are available in the Supplementary Information. The crystal structure used for the docking study is the PDB ID 7OE8 [https://doi.org/10.2210/pdb7oe8/pdb] (C-terminal bromodomain of human BRD2). Source data are provided with this paper.

Human research participants

Policy information about studies involving human research participants and Sex and Gender in Research.

Reporting on sex and gender	N/A
Population characteristics	N/A
Recruitment	N/A
Ethics oversight	N/A

Note that full information on the approval of the study protocol must also be provided in the manuscript.

Field-specific reporting

Please select the one below that is the best fit for your research. If you are not sure, read the appropriate sections before making your selection.

[x] Life sciences [ ] Behavioural & social sciences [ ] Ecological, evolutionary & environmental sciences

For a reference copy of the document with all sections, see nature.com/documents/nr-reporting-summary-flat.pdf

Life sciences study design

All studies must disclose on these points even when the disclosure is negative.

Sample size	no sample size calculations were performed. Unless otherwise indicated, experiments were performed in triplicate (independent experiments) as standard in the field, with the indicated technical replicates per experiment. For dose-response curves, the typical number of technical replicates was commonly 3, whereas for microscopy we included more replicates, especially since effect size can be small and analysis of a large number of cells/cilia yields better quality and robuster data.
Data exclusions	individual microscopy images were excluded from analysis through manual inspection for the presence of fluorescent background 'blobs', which would interfere with the quantification. Many images were taken for each condition and replicate to ensure the validity of the data even when few images could not be used.
Replication	No attempts at replication failed. All replication experiments confirmed initial findings. Most experiments were replicated at least three times, this is further specified in the figure legends.
Randomization	Some level of randomization was included, such as position on the plate when incubating with compounds, to compensate for plate effects. Further randomization was not suitable for our study as typically conditions fall either in the treated, or in the untreated 'control' group.
Blinding	Blinding was not practical or relevant to our study, as the experimenter needs to prepare the compound solutions and add them to the wells. However, all analyses were automated, thus no bias was invoked.

Reporting for specific materials, systems and methods

We require information from authors about some types of materials, experimental systems and methods used in many studies. Here, indicate whether each material, system or method listed is relevant to your study. If you are not sure if a list item applies to your research, read the appropriate section before selecting a response.

Materials & experimental systems

n/a	Involved in the study
☐	☒ Antibodies
☐	☒ Eukaryotic cell lines
☒	☐ Palaeontology and archaeology
☒	☐ Animals and other organisms
☒	☐ Clinical data
☒	☐ Dual use research of concern

Methods

n/a	Involved in the study
☒	☐ ChIP-seq
☒	☐ Flow cytometry
☒	☐ MRI-based neuroimaging

Antibodies

Antibodies used

Antibody sources are described in the Methods section.
Goat anti-mouse Gli3 (R&D systems, AF3690), 1:200 (WB), 1:500 (IF)
Mouse anti-Gli1 (Cell signaling, 2643S, lot 12), 1:1000 (WB)
Goat anti-mouse Gli2 (R&D systems, AF3635, lot XUA0320091), 1000 (WB), 1:500 (IF)
mouse anti-ARL13B (Biolegend, cat # 857602, clone N295B/66, lot B323369), 1:3000 (IF)
Mouse anti-vinculin (Proteintech, 66305-1, clone 2B5A7, lot 10016677), 1:5000 (WB)
Mouse anti-BRD3 (Santa Cruz, sc-81202, 2088C3a, lot H2521), 1:200 (WB); 1:500 (IF)
Rabbit anti BRD4 (Bethyl laboratories, A301-985A-M, lot 8), 1:1000 (WB and IF)
Rabbit anti BRD2 (Bethyl laboratories, A302-583A-T, lot 6), 1:1000 (WB), 1:500 (IF)
mouse anti-gamma tubulin (Sigma Aldrich, GTU-88, T6557), 1:500 (IF)
Rabbit anti-Phospho-ERK1/2 (Proteintech, 28733-1-AP), 1:1000 (WB)
Mouse anti-AKT-phospho-S473 (Proteintech, 66444-1-IG, clone 1C10B8), 1:1000 (WB)
Mouse anti-p65 (Cell Signaling Technologies, #6956, clone L8F6, lot 9): 1:1000 (IF)
Mouse anti Karyopherin-beta HRP conjugated (Importin beta (IMPB), Santa Cruz, sc-137016, H7-HRP lot E1519), 1:1000 (WB)

Validation

Goat anti-mouse Gli3 (R&D systems, AF3690), 1:200; validated using Gli3 KO cells in PMID:29459677
Mouse anti-Gli1 (Cell signaling, 2643S), 1:1000; validated using Gli1 KO cells in PMID:29459677
Goat anti-mouse Gli2 (R&D systems, AF3635), 1:1000; validated in Gli2 KO cells in PMID 26193634
mouse anti-ARL13B (Biolegend, cat # 857602, clone N295B/66), 1:3000; KO validated by supplier: same antibody clone as sold by NeuroMab: https://neuromab.ucdavis.edu/datasheet/N295B_66.pdf
All other antibodies listed above (Mouse anti-vinculin; Mouse anti-BRD3; Rabbit anti-BRD4; Rabbit anti-BRD2; mouse anti-gamma tubulin; Rabbit anti-Phospho-ERK1/2; Mouse anti-AKT-phospho-S473; Mouse anti-Karyopherin-beta) are extensively used in the field for the species and applications listed and have been used in dozens to hundreds of peer-reviewed publications.

Eukaryotic cell lines

Policy information about cell lines and Sex and Gender in Research

Cell line source(s)

A detailed description of all cell lines can be found in the Methods section. In short, SHH-LIGHT2, SHH-GFP, HEK239T-EcR-ShhN cells, A549, Wnt-LIGHT and SUFU-KO-LIGHT cells were provided by James Chen (Stanford University). NIH-3T3 cells (CRL-1658), Wnt3a-producing L cells (CRL-2647), and HEK293T cells (CRL-3216) were purchased from ATCC. IMCD3-FlpIn cells were a gift from David Mick (University of Saarland). HeLa cells were a gift from Aurelien Roux (University of Geneva). MB55 and MB56 were a gift from Rosalind Segal, Harvard Medical School.

Authentication

None of the cell lines used (NIH-3T3, SHH-LIGHT2, SHH-GFP, HEK239T-EcR-ShhN cells, A549, Wnt-LIGHT, SUFU-KO-LIGHT, HEK293T, A549, Wnt3a-producing L cells, IMCD3-FlpIn, HeLa, MB55, and MB56) have been authenticated. Nearly all cell lines used were murine in origin and few tests are available for authentication of mouse cell lines.

Mycoplasma contamination

All cell lines (NIH-3T3, SHH-LIGHT2, SHH-GFP, HEK239T-EcR-ShhN cells, A549, Wnt-LIGHT, SUFU-KO-LIGHT, HEK293T, A549, Wnt3a-producing L cells, IMCD3-FlpIn, HeLa, MB55, and MB56) were confirmed negative for mycoplasma.

Commonly misidentified lines
(See ICLAC register)

None of the cell lines used (NIH-3T3, SHH-LIGHT2, SHH-GFP, HEK239T-EcR-ShhN cells, A549, Wnt-LIGHT, SUFU-KO-LIGHT, HEK293T, A549, Wnt3a-producing L cells, IMCD3-FlpIn, HeLa, MB55, and MB56) are commonly misidentified.
